# Microscale Tattooing of Hydrogels and Cells: Benzoxaborole‐Driven Microcontact Printing (µCP) on Glycosylated Surfaces

**DOI:** 10.1002/anie.202501759

**Published:** 2025-07-16

**Authors:** Nazim Pallab, Eric Sperlich, Matthias Schenderlein, Anne Krüger‐Genge, Jinyuan Li, Lukas Zeininger, Zdeněk Tošner, Mariusz Uchman, Martin Reifarth

**Affiliations:** ^1^ Institute of Chemistry University of Potsdam Karl‐Liebknecht‐Str. 24–25 14476 Potsdam Germany; ^2^ Fraunhofer Institute of Applied Polymer Research Geiselbergstr. 69 14476 Potsdam Germany; ^3^ Fraunhofer Cluster of Excellence Immune‐Mediated Diseases CIMD Frankfurt am Main Germany; ^4^ Responsive Soft Materials and Interfaces Lab, Department of Colloid Chemistry Max‐Planck Institute of Colloids and Interfaces Am Mühlenberg 1 17746 Potsdam Germany; ^5^ Faculty of Science, Department of Physical and Macromolecular Chemistry Charles University Hlavova 8 128 43 Prague 2 Czech Republic

**Keywords:** Carbohydrate‐boronic acid interactions, Functionalization of cellular interfaces, Glycopolymers, Glycoproteins, Microcontact printing

## Abstract

Microcontact printing (µCP) is a widely used technique for microscale surface patterning. In this study, we present a polymer‐supported µCP method for the patterning of (bioactive) glycosylated surfaces under hydrated conditions. Patterning is achieved by direct contact with a grooved polydimethylsiloxane (PDMS) stamp, whose surface was grafted with a dopamine‐containing polymer. The polymer brushes offer an anchor for the boronic acid derivative 6‐aminobenzo[c][1,2]oxaborol‐1(3H)‐ol (ABOB), used as an ink for surface functionalization, to introduce patterns to three different surfaces as substrates: (1) monosaccharide‐modified hydrogel surfaces possessing aldose (glucose, fucose, galactose) or ketose (fructose, sorbose) functions; (2) glycosylated surfaces of polymeric microspheres; and (3) the membranes of mammalian cells, such as human primary gastric cells and others. During µCP, ABOB patterns transferred to the target surface through the formation of carbohydrate‐ABOB complexes at fully hydrated, neutral pH conditions. Fluorescence microscopy confirmed the successful transfer of ABOB patterns to glycosylated surfaces, with clear “tattoo‐like” signatures observed on hydrogels, glycosylated particle surfaces and cellular interfaces.

## Graphical abstract

We demonstrate a polymer brush‐assisted microcontact printing (µCP) to pattern glycosylated surfaces. We used a benzoboraxole derivative as an ink for surface patterning. With this novel method, we could functionalize wafer surfaces possessing glycopolymer films, surface‐glycosylated polymer microparticles as well as cellular interfaces.

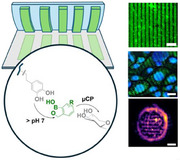

## Introduction

Microcontact printing (µCP) is a widespread surface patterning technique, which is used to add functional (sub‐) micron‐scale areas to surfaces.^[^ ^1^ ^]^ This soft lithography technique relies on a patterned elastomeric stamp to transfer reactive functional molecules, known as “ink” at a microscopic scale to a surface by direct contact.^[^ ^2^, ^3^, ^4^, ^5^ ^]^ µCP is a cost‐effective manufacturing process,^[^ ^5^ ^]^ which is straightforward to perform,^[^ ^6^ ^]^ and which is applicable on a variety of surfaces with different properties^[^ ^7^, ^8^, ^9^ ^]^ for patterning with a high spatial resolution. This feature renders µCP advantageous over other lithography techniques such as dip‐pen nanoimprint lithography technique (DPN),^[^ ^10^ ^]^ electron‐beam lithography,^[^ ^11^ ^]^ photolithography,^[^ ^12^ ^]^ etc., which often require sophisticated methodology, expensive instrumentation, and complicated experimentation strategy.^[^ ^5^ ^]^

µCP has primarily been employed on smooth metal surfaces such as gold (Au),^[^ ^13^, ^14^ ^]^ copper (Cu),^[^ ^15^ ^]^ silver (Ag),^[^ ^16^ ^]^ etc. In these examples, self‐assembled layers (SAMs) of functional molecules have been used to functionalize smooth surfaces, enabling the implementation of different surface chemistry to functionalize surfaces with reactive molecules such as amines,^[^ ^17^, ^18^ ^]^ or thiols.^[^ ^19^, ^20^ ^]^ Alternatively, macromolecules have been deployed as ink.^[^ ^21^, ^22^ ^]^ µCP, thus, finds applications across various fields, including the fabrication of sensors,^[^ ^23^, ^24^ ^]^ microelectronics,^[^ ^25^ ^]^ microfluidic devices,^[^ ^26^ ^]^ and in the studies of cell and tissue engineering.^[^ ^27^, ^28^ ^]^

In contrast to smooth surfaces, rough, and/or capillary‐active surfaces remain challenging substrates, as ink‐smearing, a diffusive distribution of the ink at the substrate surfaces, occurs, which drastically reduces the printing resolution.^[^ ^1^ ^]^ Recently, we developed a polymer brush‐assisted µCP technique, which can be used to pattern rough oxidic surfaces.^[^ ^29^, ^30^, ^31^ ^]^

Other relevant substrates, largely unchallenged by soft lithography patterning techniques, are bioactive soft surfaces. These are often only stable in a hydrated state, which has to be preserved during the printing process. Conventional µCP, however, is usually not conducted under hydrated conditions. In the context of soft surfaces, microgels represent a promising avenue toward functional materials, which show great potential in applications such as bio‐coatings,^[^ ^32^, ^33^ ^]^ sensing,^[^ ^34^ ^]^ and cell adhesion.^[^ ^35^ ^]^ Even though there are some examples, where hydrogels were patterned with biomolecules, e.g. by first freeze‐drying them prior to µCP process to be patterned by proteins to guide the alignment of cells,^[^ ^36^ ^]^ or by using methods such as microfluidic contact printing,^[^ ^37^ ^]^ these methods remain at low printing resolution. Alternative techniques, such as E‐jet printing^[^ ^38^ ^]^ or µCP using a cold polydimethylsiloxane (PDMS) stamp with condensed water as the ink,^[^ ^39^ ^]^ is used to print hydrogels directly on a substrate.

Recently, microgels with post‐modified surfaces, particularly glycosylated microgels, have gained attention for biological applications.^[^ ^40^ ^]^ For example, galactose‐functionalized polyethylene glycol acrylamide (PEGA) hydrogels have been developed to study hepatocyte cell attachment.^[^ ^41^ ^]^ Additionally, carbohydrate‐modified polyacrylamide gels, including α‐*D*‐mannopyranoside, β‐*D*‐galactopyranoside, and β‐*D*‐glucopyranoside, have been synthesized to investigate their bacteria‐capturing abilities.^[^ ^42^ ^]^ It seemed, therefore, appealing to develop a method to pattern glycosylated hydrogel surface. Having learnt lessons from the polymer brush‐assisted µCP routine developed earlier in our group, where the ink is first immobilized on the stamp surface to be transferred on the substrate, which allowed us to functionalize capillary‐active oxidic surfaces with a high local precision,^[^ ^29^, ^30^, ^31^, ^43^ ^]^ we thus aimed at adapting the principle for the precise functionalization of glycosylated surfaces.

Carbohydrates are fundamental components in living organisms, abundantly present in cells and tissues.^[^ ^44^ ^]^ As an example, the human gastrointestinal tract contains a mucus layer composed of highly glycosylated proteins, which plays a crucial role in lubricating food, facilitating cell signalling, preventing pathogen invasion, and more.^[^ ^45^ ^]^ Therefore, understanding carbohydrate‐based surfaces and their interactions with target molecules can be essential to gain insights into such processes.^[^ ^46^, ^47^ ^]^ Bioactive carbohydrates, particularly many monosaccharides, exhibit selective interactions with boronic acids.^[^ ^34^, ^48^, ^49^ ^]^ This interaction forms the basis for boronic acid‐based sensors designed, e.g. to detect glucose levels in blood samples.^[^ ^50^ ^]^ Boronic acids bind efficiently to cis‐diols in saccharides in a reversible manner, forming boronic acid–diol esters.^[^ ^51^ ^]^ This binding is an equilibrium process that is sensitive to pH, whereby at higher pH levels, the formation of boronic acid–diol complex is favored due to the transition of boronic acid to its tetrahedral boronate ion form, whereas low pH levels foster the ester cleavage.^[^ ^52^ ^]^ This distinctive property makes boronic acids highly effective as saccharide receptors,^[^ ^52^, ^53^ ^]^ e.g., for hexoses, such as d‐fructose, d‐glucose, d‐mannose, d‐galactose.^[^ ^54^ ^]^ Boronic acid–diol bond formation chemistry is also encouraging to construct bioconjugates. Biological surfaces, such as cell membranes, contain glycoproteins and glycolipids with carbohydrates at their interfaces. Therefore, carbohydrate diols can be recognized and utilized for functionalization with boronic acids.^[^ ^55^ ^]^ As an example, drug delivery was demonstrated using boronic acid‐derivatives, mediating an enhanced cellular uptake of these structures into different cell types.^[^ ^56^, ^57^ ^]^ The interaction of boronic acids with the ubiquitously present saccharides at the cellular surface was also used to modify the cell surface with a hydrophilic polymer under physiological conditions through boronic acid–diol condensation. This modification effectively blocked cell and tissue adhesion.^[^ ^58^ ^]^

Herein, we leverage the covalent interaction of surface‐bound glycosides and boronic acids in the framework of microcontact printing (µCP). Specifically, we functionalize bioactive glycosylated surfaces, such as glycosylated polymer films on a silicon wafer, polymer films on a curved surface and also membranes of cells, by transferring precise patterns of 6‐aminobenzo[c][1,2]oxaborol‐1(3H)‐ol (ABOB). Recent attempts of surface patterning of these bioactive materials involved transferring gold patterns created by nanoimprint lithography (NIL) onto alginate hydrogels and placing the patterned hydrogels to rat cells, which the authors referred to as “cell tattooing”.^[^ ^59^ ^]^ In contrast to this approach, we employ a previously established polymer‐assisted µCP method^[^ ^29^, ^30^, ^31^ ^]^ for the direct patterning of these surfaces. In our study, a patterned PDMS stamp was grafted with poly(90%*N*‐acryloyl morpholine‐*co*‐10%dopamine methacrylamide) (poly(NAM‐*co*‐DMA)) to facilitate the attachment of ABOB through catechol–boronic acid complex formation. The strong binding affinity of dopamine to boronic acids,^[^ ^60^, ^61^ ^]^ along with the enhanced ability of ABOB to form stable ABOB‐diol complexes under physiological conditions (pH 7.4 at a fully hydrated state),^[^ ^62^ ^]^ were taken into account in designing the system. Accordingly, the accurate transfer of ABOB patterns from the stamps to glycosylated hydrogels, particles and fixed cells was successfully achieved via µCP. While previous studies mainly focused on printing on glass surfaces to guide cell growth or directly printed cells on a substrate, there are not many examples where cell surfaces are patterned directly.

## Results and Discussion

**Figure 1:**
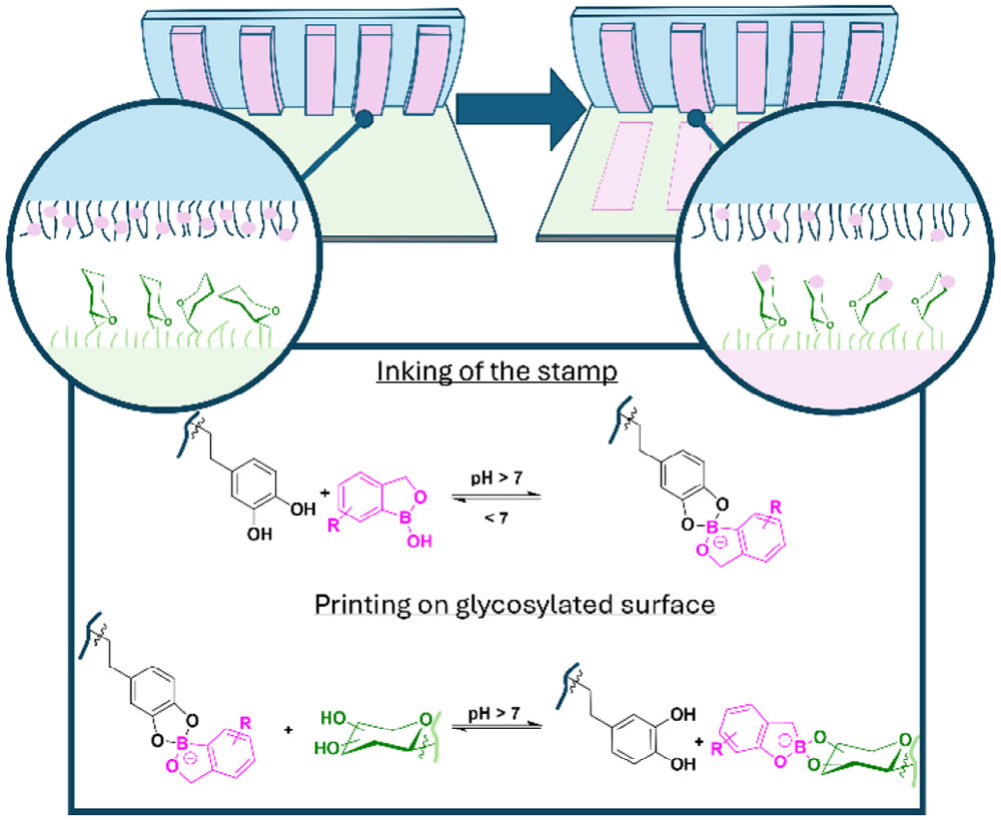
Schematic representation of the µCP process. Amino‐benzoxaborole (ABOB) conjugates with stamp‐immobilized catechol groups during the inking step. Upon printing, it forms boronic acid–sugar complexes with saccharides at pH > 7. The ABOB ink is attached to the catechol moieties of grafted polymers on a patterned PDMS stamp at pH > 7, and the ink patterns are transferred to the substrate, resulting in diol‐ABOB conjugation.

In this study, we employ a µCP method for the accurate patterning of bioactive glycosylated surfaces, specifically hexose‐modified surfaces, hydrogels, and cell surfaces, using boronic acid conjugation chemistry with catechol^[^ ^63^ ^]^ and sugar‐diols.^[^ ^49^ ^]^ More specifically, this process relies on the reversible immobilization of the ink at the stamp surface, which is achieved by adding a polymer film to the stamp surface possessing moieties for ink immobilization. During the printing process, the ink gets transferred to the substrate surface‐bound glycosides, where it will bind efficiently (Figure 1). The overall protocol of transferring the boronic acid ABOB through µCP involves, thus, three steps: (i) Grafting copolymers with moieties that are suited for ABOB immobilization onto patterned PDMS stamps, (ii) Attaching ABOB to the grafted dopamine methacrylamide units via ABOB‐catechol conjugation, and (iii) transferring ABOB from the stamp to glycosylated surfaces by forming ABOB‐diol complexes through µCP (experimental details are supported by Figures S1–S5).

The first task was the selection of an appropriate monomer to graft to the stamp surface. Previous studies have shown that dopamine‐based polymers can effectively bind boronic acids at pH levels above 7.^[^ ^64^ ^]^ This binding is facilitated by the selective interaction between the dopamine's catechol group and boronic acid, forming cyclic boronic ester complexes.^[^ ^60^, ^61^, ^65^ ^]^ To further understand this interaction and determine the optimal pH for complex formation, we analyzed a model system consisting of 4‐methylcatechol and benzoxaborole (BOB) using boron nuclear magnetic resonance (^11^B NMR) spectroscopy (Figure S6). We chose 4‐methylcatechol as its substitution pattern is similar to that of catechol in dopamine (Figure S6),^[^ ^66^ ^]^ using BOB as a model compound for the analysis. Previous studies have explored the mechanism of BOB‐catechol complexation across various pH levels via boron nuclear magnetic resonance (^11^B NMR) analysis.^[^ ^66^ ^]^ Benzoxaborole, with a pK_a_ of approximately 7.3^[^ ^67^ ^]^ can form complexes with catechol at physiological pH value under mildly basic conditions (pH > 7) as BOB transitions from sp^2^‐ to a sp^3^‐hybridized state forming a boronate ester.^[^ ^68^ ^]^ It was also demonstrated that the five‐membered ring of BOB can open due to interactions with solvent by means of ^11^B NMR (Figure S6). Our investigation of the 4‐methylcatechol‐BOB system revealed that the optimal pH for complex formation is between 7.4 and 9 with no free BOB detected in ^11^B NMR spectra, as shown in Figure S6. Conversely, ester formation is not favored in acidic environments, as merely the free BOB signal could be detected. Further information regarding the boronate ester formation with catechol and sugar are provided in the supplementary information of this study (Figure S6). In addition, to assess whether BOB can be transferred from 4‐methylcatechol to a carbohydrate as well as the optimal pH necessary for carbohydrate‐BOB conjugation, we studied the binding of BOB with *D*‐fructose by means of ^11^B NMR (Figure S6, supported by additional NMR experiments shown in Figures S7–S10). In this study, we will focus on a pH value above 7.4 during all of our experiments, which represents a physiologically relevant range and where the binding occurs most efficiently.

**Figure 2:**
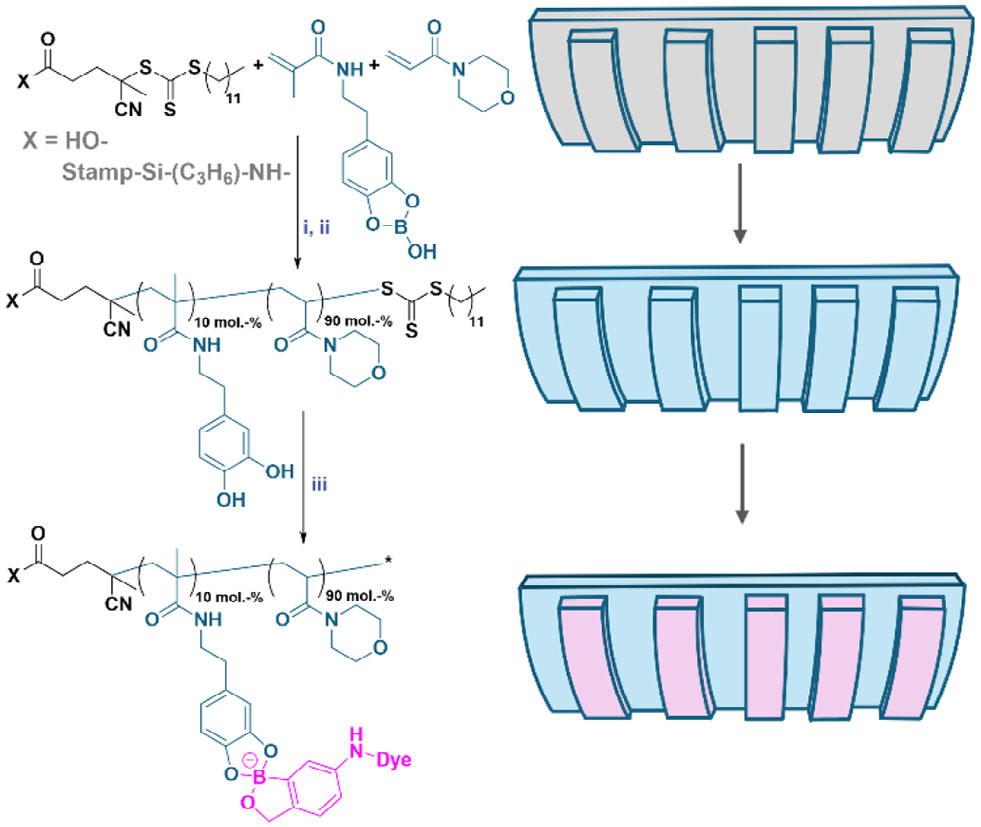
Schematic representation of the stamp grafting process. i) Grafting catechol‐containing polymers from the patterned stamp surface via RAFT process (shuttled CTA RAFT). CTA was attached to the surface at first and grafting was carried out in polymer solution in the presence of excess CTA. ii) Deprotection of catechol was done by immersing the stamp in HCl solution (pH 1). iii) The stamp was inked with ABOB (conjugated with RhITC) in a PBS solution (10 µg mL^−1^).

Having demonstrated the binding between BOB and catechols as well as BOB and sugars, dopamine‐based materials serve as an appropriate anchor for immobilization of the ink at the stamp surface. We, thus, started with the preparation of the polymer brush stamp. Poly(90%*N*‐acryloyl morpholine‐*co*‐10% dopamine methacrylamide) (poly(NAM‐*co*‐DMA)) was grafted from the surface of a PDMS stamp, exhibiting a groove relief, using the reversible addition‐fragmentation chain transfer (RAFT) polymerization technique.^[^ ^69^ ^]^ For this purpose, the stamp surface was first functionalized with 3‐aminopropyl triethoxysilane (APTES), followed by the attachment of 4‐Cyano‐4‐[[(dodecylthio)carbonothioyl] thio]pentanoic acid, which is used as the chain‐transfer agent (CTA). Surface‐initiated RAFT polymerization was conducted with the corresponding monomers using a shuttle CTA approach,^[^ ^70^ ^]^ allowing characterization of surface‐grafted polymers based on the polymers formed in solution.^[^ ^30^ ^]^ We opted for a higher feed ratio of NAM (∼90%) to DMA (∼10%) to ensure the final polymer's water solubility, aiming toward a mere aqueous‐based µCP chemistry. However, this occurs at the expense of sufficient ink immobilization, which is favored by a high DMA content. A low DMA content also prevents quick oxidation of the free catechol moieties in the copolymer. In order to avoid an interference of the free catechol groups in the radical polymerization process, and to avoid an oxidation of DMA,^[^ ^71^, ^72^ ^]^ we also protected the DMA monomer (DMA‐p) with borax prior to the polymerization step as depicted in Figure 2.^[^ ^72^ ^]^ Note, that cyclic boronic esters as shown in Figure 2 along with negatively charged hydroxide adducts of said esters are present at ambient pH values. After the grafting process, the dopamine (DMA‐p) was deprotected by washing the stamp with an acidic hydrochloric acid solution (pH 1) (Figure 2). The representative NMR spectra of the monomer and polymer as well as the SEC are shown in Figures S11–S18. In a subsequent step, ABOB, functionalized with a functional fluorescent label, was used for stamp inking.

In order to validate the concept of transferring boronic acid patterns onto (bioactive) glycosylated surfaces, we, first, fabricated various glycoside functionalized surfaces. For this purpose, we functionalized Si wafer surfaces using five selected monosaccharides, including both ketose (*D*‐fructose, *L*‐sorbose) and aldose sugars (*D*‐glucose, *D*‐galactose, *L*‐fucose), synthesized as outlined in Figure S19.

**Figure 3:**
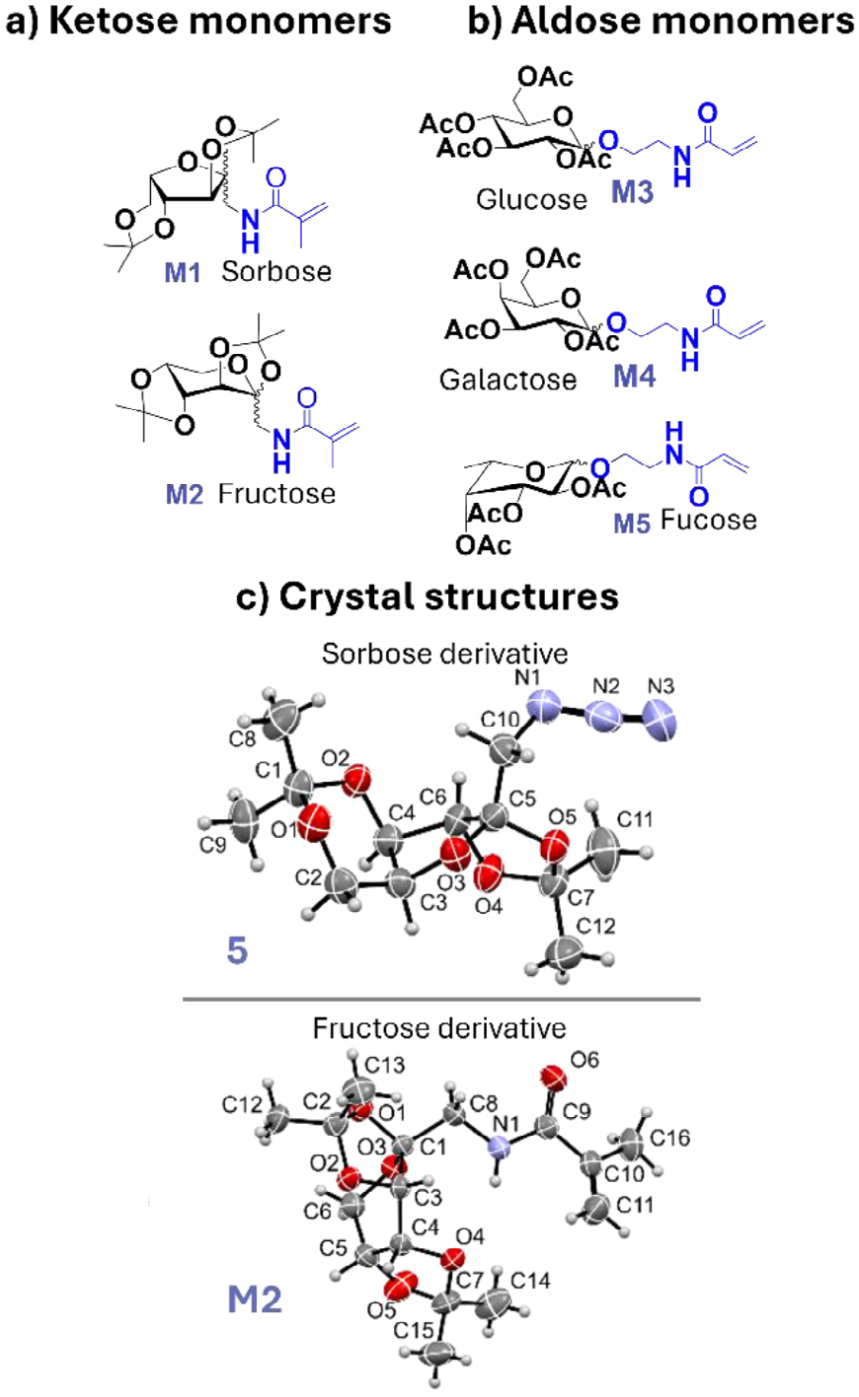
Chemical structures of saccharide monomers: a) ketose and b) aldose monomers. c) Illustration of representative crystal structures obtained for the intermediate azide 5 (see Figure S19 for all the intermediates) of Sorbose and final methacrylamide monomer M2 of fructose.

Initially, the ketose sugars (*D*‐fructose and *L*‐sorbose) were used in their protected forms containing the di‐isopropylidene acetal groups, leaving only the anomeric ‐CH_2_OH groups available for further transformation. Interestingly, fructose was present in its pyranose form, whereas sorbose formed the furanose ring in its protected form, as confirmed by X‐ray crystallography of an intermediate **5** and the final monomer **M2** (Figure 3c).  The ‐CH_2_OH groups were converted to their respective azides using triflate intermediates for subsequent reduction to amines in a *Staudinger*‐type reaction. Finally, the amines were transformed into methacrylamides (compounds **M1** and **M2** in Figure 3). Crystals were obtained at intermediate steps and for the final monomer **M2** (Figure 3c and S36–S39), including Table S1 containing relevant information).

To obtain aldose monomers, aldose sugars were first penta‐acetylated using acetic anhydride, then transformed to their acrylamide derivatives **M3, M4, M5** (Figures 3b and S19) as synthesized according to the existing literature.^[^ ^73^, ^74^ ^]^ All analyzed NMR spectra (Figures S20–S35) and mass spectra (Figures S40–S54) are included in the supporting information.

**Figure 4:**
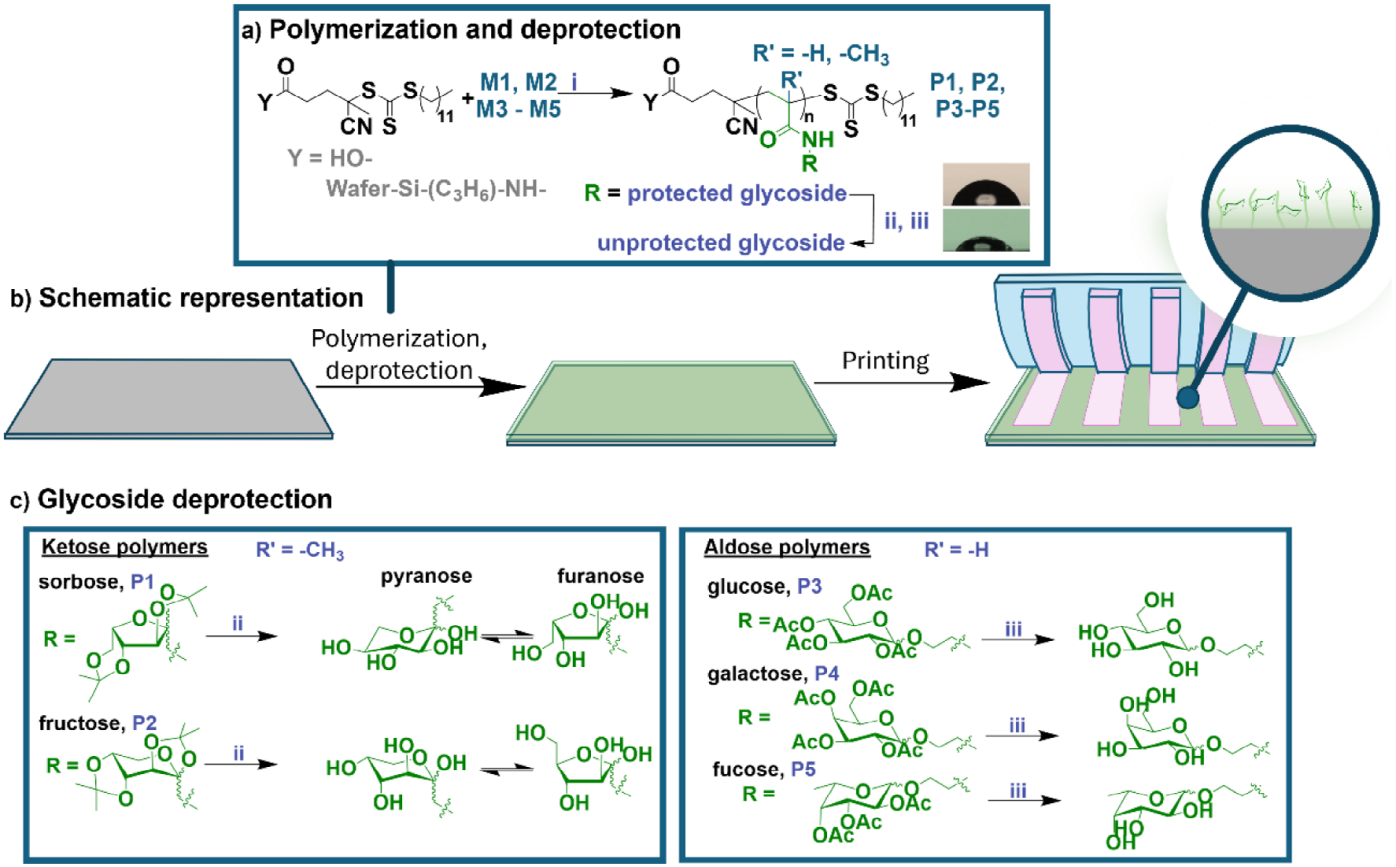
a). Illustration of wafer functionalization and deprotection strategy of saccharide functionalized surfaces. The deprotection for ketose polymer was carried out in ii) TFA/water solution, whereas iii) NaOMe solution in MeOH was used for aldose polymers. The wafer was first functionalized with chain transfer agent (CTA) and then the polymers were i) grafted from the surface and made prepared for µCP. b) Schematic representation and illustration of grafted sugar polymers from the surface. c) Illustration of the glycosidic deprotection of the grafted sugar polymers. The inset in ‘a)’ shows that water contact angle decreases after deprotection, illustrated for grafted sorbose polymer (P1), deprotected wafer on bottom; contact angle decreases from 74.85 ± 3.97 to 53.41 ± 1.08 degrees.

The synthesized monomers were then polymerized via RAFT polymerization as well as the reaction kinetics were analyzed in solution portraying that considerable conversion of both ketose methacrylamides and aldose acrylamides were achieved after 24 h. Nevertheless, a lower conversion for ketose monomers was registered than aldose monomers (ca. 70% compared to ca. > 85% after 24 h, Figure S61 **)**. Accordingly, RAFT polymerization technique was employed to prepare glycosylated Si wafer surfaces by grafting saccharide‐polymers (Figure 4a,b) using the aforementioned shuttled CTA RAFT from the surface.

^1^H NMR spectra and size‐exclusion chromatograms (*M*_n_ and Đ are shown in Table S2) of the polymers formed in solutions are presented in Figures S55–S60. Sugar deprotection was conducted subsequently to the grafting process. For ketose polymers, we used a trifluoroacetic acid (TFA)/H_2_O solution to release the isopropylidene protection groups. The deprotected ketose sugars may be present in the pyranose and furanose form, which coexist in an equilibrium (Figure 4c). Acetyl groups protecting the aldose sugars were deprotected using a methanolic sodium methoxide (NaOMe) solution. Water contact angle measurements confirmed that the deprotection was successful, with surfaces retaining the protected hydroxyl groups (‐OH) showing a hydrophobic character (contact angle ∼70°) and deprotected surfaces exhibiting a more hydrophilic nature (contact angle ∼60°, see the inset in Figure 4a). The representative images and average water contact angles are shown in detail in Figures S62 and S63. To pattern the prepared glycosylated surfaces, a catechol‐grafted stamp possessing 4 µm groove patterns was inked with ABOB. First, the functional ABOB ink was coupled with a fluorescent dye, namely Rhodamine B isothiocyanate (RhITC), in DMSO.^[^ ^75^ ^]^ The stamp was immersed in the ink solution and washed with the buffer solution to remove any excess dye. The ink was next transferred from the stamp onto the prepared sugar‐modified Si wafers through µCP, maintaining an aqueous environment buffered at a pH value of approx. 7.4. We used a small chamber, where we immersed both the substrate and the stamp.

**Figure 5:**
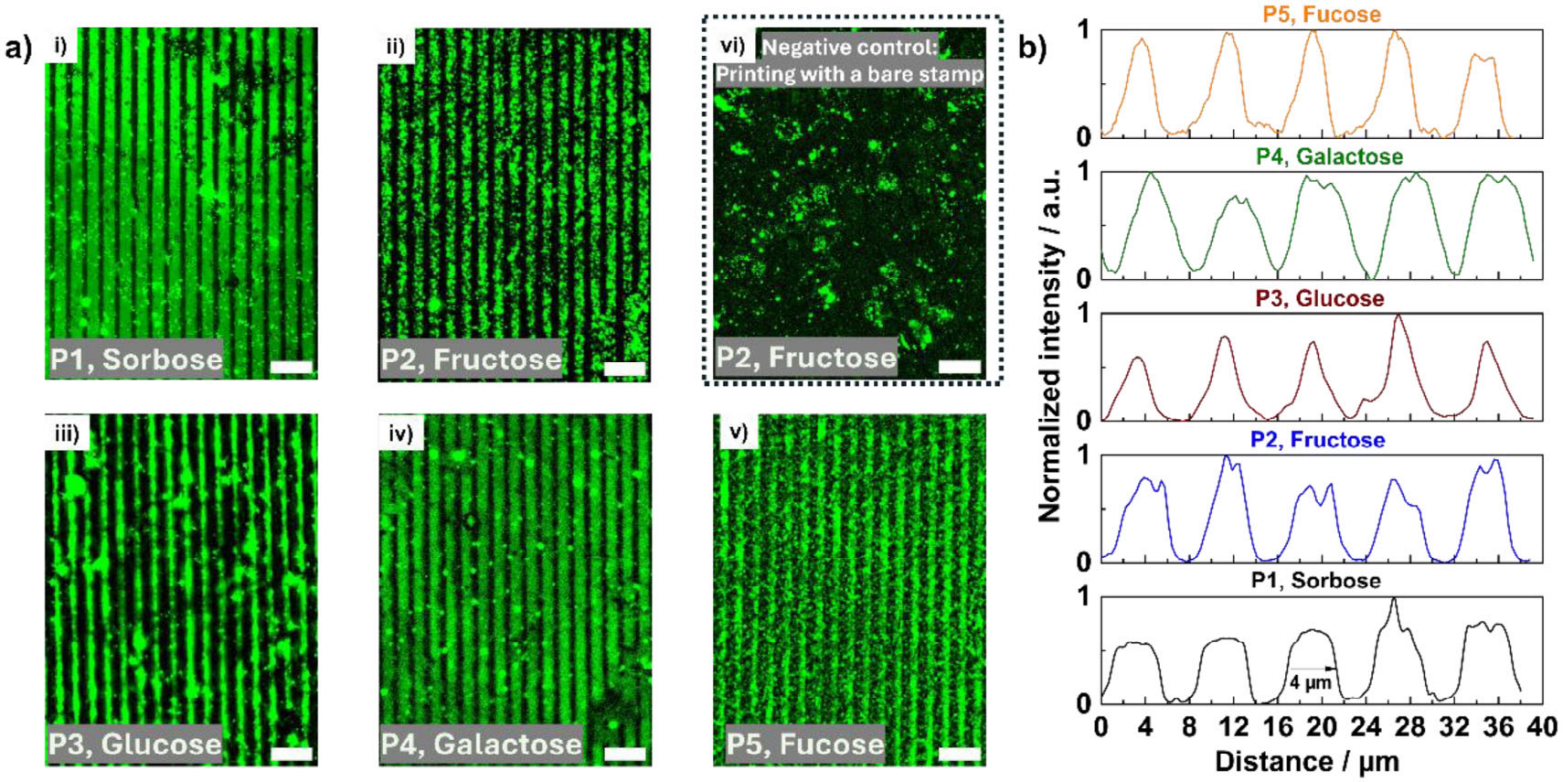
a). i)–v) shows fluorescence microscopy images of precise patterns of ABOB (RhITC conjugated) transferred onto glycosylated Si wafer surfaces. Printing time for ketose modified substrates, and fucose are 20 min, in contrast printing time for galactose and glucose are 30 min. vi) No patterns were visible on a printed fructose surface when printing was carried out with a bare stamp (without any grafted polymer, negative control). b) The intensity plots versus distance (µm) of the patterned area of respective sugar modified substrates. P1, P2, P4 show accurate patterns, in contrast, P3 and P5 are comparatively less precise than the formers. The images were processed with ImageJ. The scale bars are 20 µm. b) Intensity plots derived from the images from a) (relative intensities of the fluorescence signals).

Using fluorescence microscopy, we could verify the transfer of the ink, as stripe patterns could be observed on all glycosylated surfaces as shown in Figure 5a. To assess the optimal printing time required for uniform pattern transfer, a series of printing experiments for different printing times was conducted. While printing for 2 and 5 min shows fractional and incomplete pattern formation on ketose‐modified surfaces, higher printing time generates better coverage and accuracy of the pattern on the surface as indicated by fluorescence microscopy. In contrast, aldose‐modified surfaces showed negligible pattern formation at shorter printing times, with more defined patterns observed only after 30 min of printing (Figure S64). Notably, the sorbose‐modified surface displayed more precise and uniform patterns across the whole surface almost for all different printing times, observed under the microscope compared to other glycosylated surfaces, attributed to the higher binding constants of the former.

Additionally, in Figure 5b the normalized fluorescence intensity is plotted against distance for quantification, depicting consistent 4 µm patterns of ABOB. In general, ketose‐modified substrates along with galactose demonstrate more accurate pattern formation, in contrast to glucose and fucose modified surfaces.

To further substantiate the necessity of the polymer brush stamps, a control experiment was conducted on a fructose‐modified surface using a non‐grafted bare stamp. This stamp was inked and used to print onto the fructose surface, but the resulting fluorescence microscopy image revealed a plain surface with no visible patterns (Figure 5a‐vi), indicating that polymer‐assisted µCP is essential for the precise pattern transfer.

Being based on a reversible binding chemistry, we also determined if the stamp can be reused several times to make the printing process more efficient and sustainable. Accordingly, a single grafted stamp was used to print on sorbose modified substrates up to four times, when it was re‐inked after each printing process as indicated by fluorescence microscopy (Figure S65). As the formation of the ester is pH‐reversible, we wanted to probe whether printing patterns can be erased in an acidic environment. For this reason, a printed substrate (fructose) was also washed with pH 4 buffer solution and observed under the fluorescence microscope. As shown in Figure S66, the pattern significantly faded under these conditions.

The observed differences in pattern uniformity may depend on several factors, including the binding affinity of the respective sugars for ABOB, the molar ratio of two components in a complex, and their structural orientation during the printing process. Saccharides, particularly in the solution, can undergo mutarotation allowing them to exist in different configurations, which influences the binding affinity of the boronic acid–sugar complex. Boronic acids typically prefer to form complexes with saccharides when these sugars are in their furanose forms.^[^ ^76^ ^]^ Their binding constants depend significantly on the availability of these furanose forms (α and β) in solution. This means that higher proportion of α‐ and β‐furanose compared to the pyranose in aqueous equilibrium constructs a higher binding constant of a sugar to a specific boronic acid.^[^ ^51^ ^]^ As an example, d‐fructose exhibit a significantly higher binding constant with BOB at pH 7.5 compared to d‐glucose, suggesting that the percentage of fructofuranose in an aqueous medium is higher than glucofuranose.^[^ ^50^, ^53^, ^77^ ^]^

To gain further insight into the binding affinity of sugars to ABOB and to correlate the findings with the printing results, we investigated the binding affinities of various sugars with the model boronic acid BOB. We conducted competitive binding assays using Alizarin Red S (ARS) and the respective sugars to measure their binding constants with BOB:^[^ ^53^ ^]^

BOB+carbohydrate⇌carbohydrate·BOB.

**Figure 6:**
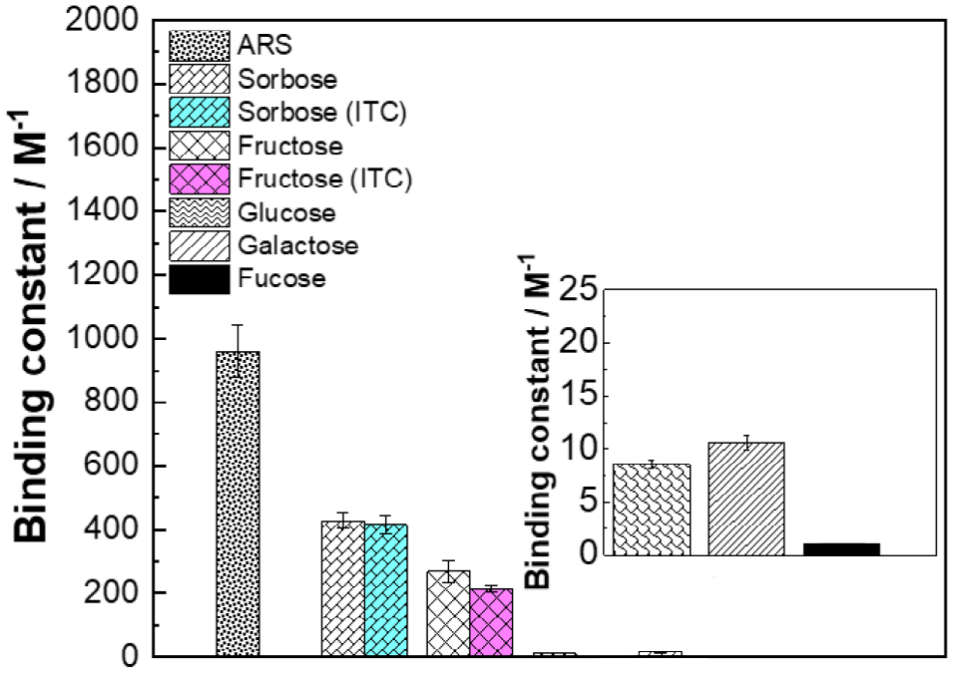
Binding constant of different monosaccharides (sugars) with benzoxaborole (BOB) measure via binding assay based on fluorescence intensity. Binding constants were derived from the graph plotted according to the calculated value based on the concentrations of ARS and sugars, as well as their fluorescence intensities. The graphs are included in Figures S67 and S68. The binding constants of fucose could not be determined in the concentration range explored (shown in black). All values are displayed as means of a triplicate of measurements including a standard deviation.

In this assay, non‐fluorescent ARS binds to boronic acid, forming a fluorescent complex that becomes non‐fluorescent as boronic acid interacts with sugar to form a boronic ester, releasing ARS. Measuring the fluorescence intensities of the solution provides access to the binding constants.^[^ ^53^ ^]^ Binding assays were conducted for various sugars, including fructose, sorbose, galactose, glucose, and fucose at a physiological pH of 7.4. It is noteworthy that during the synthesis of the monomer, the anomeric ‐CH_2_OH group was modified and subsequently attached to the surface during the grafting process. This modification may lead to conformational changes and influence the binding orientation of sugar molecules when forming bonds with boronic acids.^[^ ^78^ ^]^ The resulting binding constants are presented in Figure 6. The binding constant of ARS with BOB was found to be (960 ±  81) M⁻¹. Among the sugars tested, sorbose showed the highest binding constant (approximately (429 ±  25**) **M⁻¹), followed by fructose (around (270  ±  34) M⁻¹), aligning with previous results.^[^ ^78^ ^]^ The binding constants for glucose and galactose are ((12 ±  0.41) M⁻¹ and (15 ±  0.65) M⁻¹), respectively. The observed trend in binding constants is:

sorbose>fructose≫galactose>glucose.

For fucose, accurate determination of binding constants was not possible in the concentration range examined, pointing toward a less effective binding. Showing an enhanced binding constant, the ketose sugars sorbose and fructose deserve particular attention. Their binding affinity was further determined using isothermal titration calorimetry (ITC). The assessed values, sorbose  =  (417 ±  28) M⁻¹ and fructose  =  (215 ±  11) M⁻¹, are consistent with those obtained from the binding assays (Figure 5 and Figures S67 and S68). All these findings confirm that variations in sugar's binding affinity to boronic acids can impact printing quality, as indicated by more accurate patterns found for sugars that possess a high binding affinity with BOB.

As an additional measurement, we performed exchange rate spectroscopy (EXSY) NMR experiments to assess the binding of BOB with d‐fructose and l‐sorbose (Figure S10). The experiments were conducted to analyze the chemical exchange between BOB and its corresponding BOB‐ester with sugars, specifically fructose (FRU) and sorbose (SOR), in solution. Figures S7–S10 show the NMR spectra along with necessary explanation. We estimate the lifetime of BOB:FRU and BOB:SOR complexes to be approximately 0.7 and 7.8 s, respectively. These findings confirm that the fructose‐BOB interaction is weaker than sorbose‐BOB interaction, as weaker binding typically allows for more rapid association and dissociation dynamics, leading to faster exchange rates in NMR experiments. These results are in good accordance with the findings obtained by ITC and the competitive binding assay.

**Figure 7:**
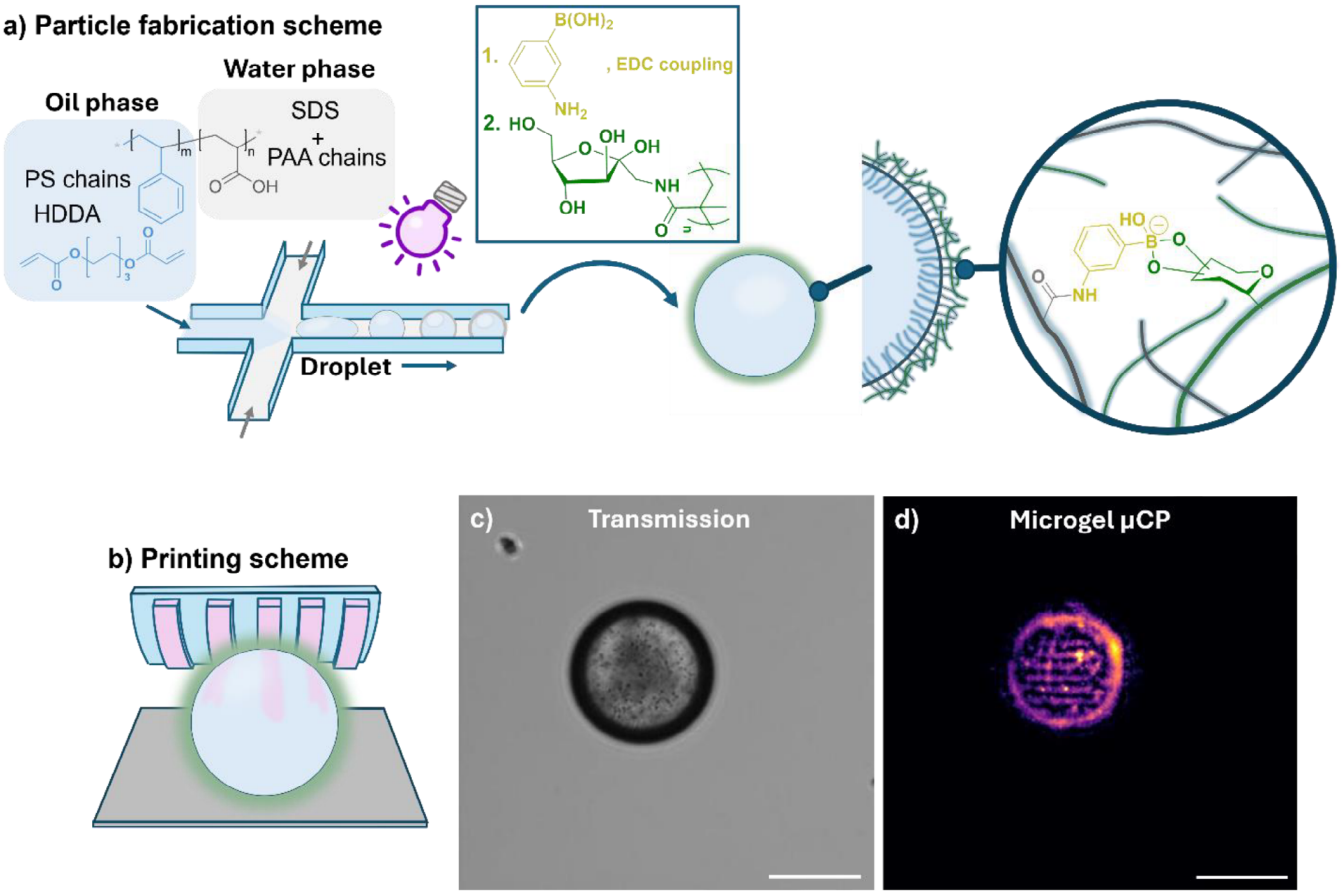
a) Generation of microgel particles by microfluidics and fabrication of fructose functionalized 1,6‐Hexanediol diacrylate (HDDA) microgel particles combined with PS‐*b*‐PAA chains as shown on top. After particle formation the particles were first UV polymerized and then functionalized with PBA. The fructose polymer P2 was acetal deprotected and the free hydroxy groups were bound to PBA groups of the microgels in Tris buffer (pH 8.5). Non‐bound ‐*OH* groups were accessed with ABOB during µCP. b) Illustration of µCP on the hydrogel after attaching them on a glass surface. The surface was functionalized that offers boronic acid anchor to attach the sugar functionalized particles from one end. c) Transmission and d) fluorescence microscopy image of the printed stripe patterns on the microgel particle. The scale bars are 100 µm.

After successfully transferring ABOB to patterned sugar surfaces, we next extended our µCP technique to more intricate systems. In order to demonstrate the suitability of the method to pattern soft materials with a curved surface, we prepared sugar‐decorated polymer particles. More specifically, we used a droplet template to prepare phenylboronic acid (PBA)‐functionalized 1,6‐hexanediol diacrylate (HDDA) micron‐sized (with an average diameter of 80 µm) particles using a microfluidic setup (Figure 7 and Figure S69a).

To this end, HDDA droplets were stabilized using poly(styrene)‐*b*‐poly(acrylic acid) (PS‐*b*‐PAA) block‐*co*‐polymer used as surface‐active macromolecules and subsequently hardened via UV‐induced free radical polymerization (Figure 7a). In a post‐modification, 3‐aminophenyl boronic acid (APBA) was attached to the hydrophilic PAA functionalities on the particle surfaces, yielding PBA‐functionalized microgel particles. The availability of PBA at the surface was assessed by attaching fluorescein isothiocyanate (FITC)‐conjugated dextran to the particles and successful binding confirmed by fluorescence microscopy (Figure S69a). To prepare the substrates, we decorated the microparticles with fructose at the particle surface. Accordingly, **P2** was synthesized and treated with a trifluoroacetic acid (TFA/water) solution for glycosidic deprotection, yielding the fructose‐containing methacrylamide. The polymer was conjugated to the PBA‐functionalized particles in tris buffer (pH 8.5) via boronic acid–diol interactions, forming sugar‐functionalized microparticles (Figure 7a). The particles were subsequently immobilized onto ABOB‐functionalized glass substrates through additional boronic acid–diol conjugation, leveraging the unbound diols in the sugar polymers. Finally, µCP was performed using an ABOB‐RhITC‐inked stamp on the sugar‐functionalized microgel particles in a buffered solution (pH 8.5, Figure 7b). Fluorescence microscopy revealed well‐defined stripe patterns, confirming the successful transfer of ABOB onto the microgel surfaces and the formation of ABOB‐diol complexes (Figure 7c,d and Figure S69b **)**.

**Figure 8:**
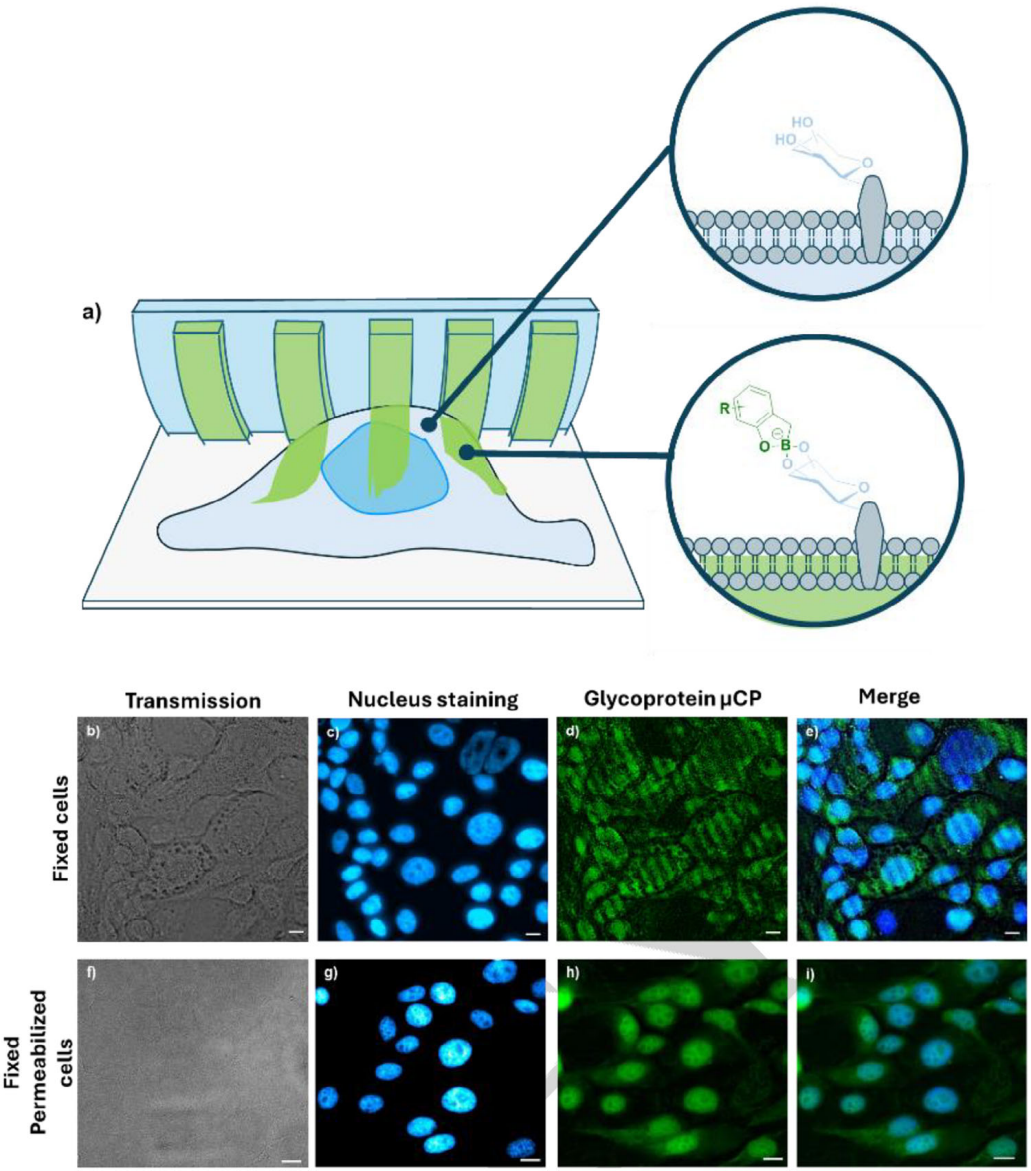
a). Illustration of µCP on fixed cells. b) – e) shows the fluorescence microscope images of printed cell surfaces. c) Transmission channel, c) DAPI stained nucleus, d) fluorescence channel, and e) combined channels are showing transferred ABOB‐FITC patterns. f) – i) shows the fluorescence microscope images of triton‐X treated cell surfaces. Transmission channel f), DAPI channel g), and FITC channel h) are combined to generate final image i). Background subtraction, color assignment via lookup table, brightness, and contrast corrections were carried out with imageJ. The scale bars are 20 µm.

As another relevant hydrogel with a glycosylated surface, we applied the method to pattern the membranes of cells confluently attached to a glass surface. Primary human gastric epithelial cells were selected for this purpose, since these are intrinsically functionalized with glycoproteins, which add carbohydrate‐functionalities to their surface,^[^ ^45^ ^]^ bearing glucose, galactose, and fucose molecules at cellular interfaces. Prior to printing, the cells were fixed using formaldehyde. To visualize the cell nuclei under a fluorescence microscope, the cells were stained with 4′,6‐Diamidin‐2‐phenylindole (DAPI) before printing. Patterning was performed with an ABOB‐FITC inked stamp in a pH 7.4 buffer in accordance with the protocol optimized for hydrogel printing. Clearly, stripe patterns appeared on the cell surfaces under the fluorescence microscope (Figure 8b–e), which can be referred to *cellular tattoos*. With ABOB, we introduced a fluorescent dye, which is used as a label for imaging. In order to confirm that the patterns are introduced as BOB‐carbohydrate complexes with cellular carbohydrates, we also performed printing on cells that had previously been treated with Triton‐X. Triton‐X, a surfactant, can solubilize cell membranes, potentially removing sugar anchors from the membrane surface and disrupting boronic acid–sugar interactions.^[^ ^79^ ^]^ Microscopy analysis revealed no patterns transferred onto these surfaces (Figure 8f–i), indicating that cellular glycans are crucial for the boronic acid‐based patterning. Additional experiments involved treating the cells with acetic anhydride to passivate the amino functionalities of membrane proteins. The results showed clear patterns on the cell membranes (Figure S70a–d), indicating that ABOB primarily interacts with the sugar moieties on the cell membrane rather than amino functionalities that could be presented from lysine moieties of membrane proteins.

Printing was also conducted using a bare inked stamp (without grafted polymer). Typically, no patterns were observed, though a few faint patterns were seen in some instances (Figure S70i‐l).

This suggests that polymer‐brush assisted ink transfer is necessary for the selective transfer of ABOB from the stamp to the sugar substrate. We also explored whether the printed patterns could be erased in an acidic environment. The coverslip with printed cells was immersed in a pH 4 buffer solution to cleave the boronic acid–diol bonds. Most patterns faded after washing (shown in Figure S70e‐h). Lastly, to further evaluate and confirm the ABOB transfer on the cell membrane of different cell types, L929 fibroblast cells were fixed on the cover slip. Printing on these cells clearly shows distinct ABOB patterns under the microscope (Figure S71a‐d). The complimentary negative control using an inked bare stamp reveals no patterns on the cell membrane (Figure S71e‐h). This demonstrates that sugar‐based biological surfaces, such as cell membranes, can be effectively patterned or “tattooed” using µCP through boronic acid–diol interactions. It should be noted that our approach is not limited to printing an ABOB adduct of a fluorescent label. We envision the attachment of more functional (bio‐)molecules to the printing patterns, such as proteins or antibodies. Using the amino functionality of ABOB as a chemical handle, we could convert its amino group into functionalities that are able to engage in click reactions, such as azides, maleic imides, alkynes, and others.

## Conclusion

In summary, our study successfully demonstrates the application of microcontact printing (µCP) leveraging pH‐dependent boronic acid‐saccharide complexation to functionalize bioactive glycosylated surfaces. By employing a polymer‐assisted µCP method with a PDMS stamp grafted with poly(90% *N*‐acryloyl morpholine‐*co*‐10%dopamine methacrylamide), we achieved precise pattern transfer of 6‐aminobenzo[c][1,2]oxaborol‐1(3H)‐ol (ABOB) onto glycosylated surfaces, including flat solid substrates, spherical polymer particles, and cell membranes. The binding affinity studies were also performed with several monosaccharides with benzoxaborole (BOB) to understand the relationship between the binding affinity and printing quality. Binding assays revealed that the sorbose has a higher binding constant than other sugars that correlates to a precise pattern transfer by forming an effective ABOB‐diol complex. Successful patterning on cell membranes further validated the method's efficacy for biocompatible applications. Given these examples, patterning of glycosylated surfaces allows for fabrication of sensitive devices for selective sensing of saccharides and can be extended to construct targeted cell‐interaction platforms. Particularly, µCP is well known for guiding cell growth,^[^ ^27^ ^]^ thus, we can direct the growth of cells on a layer of other cells, which can be used for tissue engineering. However, this study also highlights the need to explore a broader range of boronic acid and saccharide pairings, mainly, multivalent boronic acids with oligo‐ and polysaccharides within µCP domain to better understand the surface patterning behaviors and expand functional applications. Overall, this study expands the potential applications of µCP in biomaterials and cell biology, offering a robust method for surface functionalization and patterning with high precision.

## Supporting Information

The authors have cited additional references within the Supporting Information.^[^ ^80^, ^81^, ^82^, ^83^, ^84^, ^85^, ^86^, ^87^ ^]^

## Conflict of Interests

The authors declare no conflict of interest.

## Supporting information



Supporting Information

## Data Availability

The data that support the findings of this study are available in the supplementary material of this article.
