# Supplementary material for: Microscale Tattooing of Hydrogels and Cells: Benzoxaborole‐Driven Microcontact Printing (µCP) on Glycosylated Surfaces
Source: Angew Chem Int Ed Engl. 2025 Jul 16;64(35):e202501759. doi: 10.1002/anie.202501759 (PMC12377447; doi:10.1002/anie.202501759)
Supplement: Supplementary file 1 — Supporting Information [file ANIE-64-e202501759-s001.pdf]

Supporting Information

Microscale Tattooing of Hydrogels and Cells: Benzoxaborole-Driven Microcontact Printing (µCP) on Glycosylated Surfaces

Nazim Pallab,[ab] Eric Sperlich,[a] Matthias Schenderlein,[b] Anne Krüger-Genge,[bc] Jinyuan Li,[d] Lukas Zeininger,[d] Zdenek Tošner,[e] Mariusz Uchman,[e] and Martin Reifarth.[ab]*

[a]Institute of Chemistry, University of Potsdam, Karl- Liebknecht- Straße 24-25, 14476 Potsdam, Germany

[b]Fraunhofer Institute for Applied Polymer Research, Geiselbergstraße 69, 14476 Potsdam, Germany

[c]Fraunhofer Cluster of Excellence Immune-Mediated Diseases CIMD, Frankfurt am Main, Germany

[d]Max-Planck Institute of Colloids and Interfaces, Am Mühlenberg 1, 14476 Potsdam, Germany

[e]Faculty of Science, Department of Physical and Macromolecular Chemistry, Charles University , Hlavova 8, 128 43, Prague 2, Czech Republic

**** To whom correspondence should be addressed:***

martin.reifarth@uni-potsdam.de

Table of Contents:

1. Experimental Details 2
1.1 Instrumentation 2
2. Materials and Methods 4
2.1 Monomer preparation for grafting from the stamp surface 4
2.2 Fabrication of stamp for grafting 5
2.3 Syntheses of monomers with monosaccharides (sugar monomers) 6
2.3.1 Ketose monomers (sorbose derivatives): 6
2.3.2 Ketose monomers (fructose derivatives) 8
2.3.2 Aldose monomers 9
2.4 Binding constant determination 10
2.5 Ink preparation: 11
2.6 RAFT polymerization of sugar monomers: 12
2.7 Preparation of sugar-substrates: 12
2.8 Preparation of microgel particles and cells 12
3. Figures and Tables 15
3.1 Boronic acid and sugar interaction: 15
3.2 Monomers for grafting from the stamp: 20
3.3 Sugar monomer syntheses and characterization 24
3.3.1 Synthesis of sugar monomers 24
3.3.2 Ketose monomers (*L*-sorbose derivatives): 25
3.3.3 Ketose monomers (*D*-fructose derivatives): 26
3.3.4 Aldose monomers (intermediates and final monomers): 27
3.3.5 Crystallographic Data: 33
3.3.6 Crystal structures of Intermediate compounds of ketose monomers: 34
3.3.7 Mass Spectra of the synthesized intermediates and final ketose and aldose monomers: 37
3.4 ^{1}H NMR spectra of representative sugar polymers in solution: 50
3.4.1 Ketose polymer (fructose): 50
3.4.2 Aldose polymers: 51
3.4.3 Kinetic study of saccharide polymerization in solution: 53
3.4.4 Water contact angle measurements (sugar modified surfaces): 54
3.5 Printing on Sugar substrates 56
3.5.1 Printing on Sugar Substrate (printing for different duration): 56
3.5.2 Printing on Sugar Substrate (multiple printing): 57
3.5.3 Printing on Sugar Substrate (negative control, pH 4 buffer wash): 57
3.6 Binding assay: 58
3.6.1 competitive binding assay (BOB-ARS and BOB-sugar complexes): 58
3.6.2 Binding assay (BOB-fructose and BOB-sorbose complexes): 59
3.7 Printing on microgel particles: 60
3.8 Printing on cell membrane: 61
4. References 62

1. Experimental Details

1.1 Instrumentation

NMR Spectroscopy. ^{1}H (400 MHz), ^{19}F (377 MHz), ^{11}B (128 MHz), and ^{13}C (101 MHz) NMR spectra were recorded on a Bruker spectrometer in DMSO-d_6, $CDCl_3$, and D_2O. The spectra were calibrated on the residual solvent peak (2.50 ppm for DMSO (d_6), 7.2 for $CDCl_3$ and 4.7 for D_2O for ^{1}H NMR). Exchange NMR spectroscopy (EXSY) was recorded on a Bruker Avance III (600 MHz) instrument in D_2O PBS buffer (pH 7.45, 0.1 M). MestReNova (12.0) was used for data evaluation.

Size exclusion Chromatography (SEC). SEC measurements were performed with devices obtained from Agilent Technologies (PSS, Mainz, Germany). Reaction solutions of sugar-based polymers were measured in THF, and the device was equipped with polystyrene (PS), standard calibration at 40 °C with a 300 × 8 mm^2 stationary PSS SDV linear M column. Measurements in an aqueous solution containing 0.3 vol% formic acid and 0.1M NaCl were carried out with a flow rate of 1 mL min^{-1}, calibrated with polyvinylpyrrolidone (PVP) standards at 40°C, using a PSS NOVEMA Max column.

Mass spectra analysis. ESI mass spectra were recorded using an ESI-Q-TOFmicro (Quadrupol-Time of Flight), Micromass Waters Inc., UK, equipped with an ESI source in positive ion modus. Mass spectra were measured in acetonitrile.

Contact Angle measurements (CA). Water contact angle measurements were conducted using a DataPhysics instrument. For CA determination, drop shapes were analyzed through the integrated SCA20 software (version 5.0.41).

X-ray structure analysis. The crystal structures were determined by single crystal structure analysis. Suitable single crystals were selected using a Leica M205C light microscope and separated with oil. X-ray crystal structure analyses were performed on a Stadivari diffractometer (Stoe) with monochromated Mo-*K*α radiation (λ = 0.71073 Å). The data correction was performed using the program X-Area (software package for collecting single-crystal data on STOE area-detector diffractometers, for image processing, for the correction and scaling of reflection intensities and for outlier rejection, STOE & Cie GmbH, Darmstadt). The structures were solved by direct methods and refined against F^2 on all data by full-matrix least-squares using the SHELX suite of programs.[1,2] All non-hydrogen atoms were refined anisotropically; the hydrogen atoms were placed on calculated positions. **Table S1** was created using FinalCif (Kratzert, D. FinalCif V123). The crystal structures were visualized with Mercury.[3] The data (**DIF-N$_3$**: CCCD 2393133; **DIFAm**: CCCD 2393131; **DISOTf**: CCCD 2393174; **DIS-N$_3$**: CCCD 2393305) can be obtained free of charge from the Cambridge Crystallographic Data Centre, http://www.ccdc.cam.ac.uk.[4]

Competitive Binding Assay. Measurements were carried out on a TECAN infinite 200 microplate readers with a standard Greiner 96-well plate. The samples were excited at 468 nm and the emission was measured at 572 nm. Fluorescence measurements were acquired with software: i-control 1.9.

Isothermal titration calorimetry (ITC). Isothermal titration calorimetry (ITC) measurements were conducted at 25°C using a MicroCal PEAQ-ITC calorimeter from Malvern Panalytical.

Microfluidic device. Monodispersed droplets were generated by using the X-junction microfluidic chip (100 μm) purchased from Dolomite, with microfluidic pump from Fluigent. Milli-Q water with 1wt% SDS was employed as continuous phase, and HDDA with 0.5wt% PS-*b*-PAA was dispersed phase. The droplets were collected and polymerized under 375nm UV light and washed by isopropanol and Milli-Q water three times.

Microcontact Printing (μCP). Printing was conducted using a ZumoLab-microcontact printer (Zumo-MCP, KS series; 0.2 kN, 2 mV V-1, SN: #68180, #68181) from ZUMOLab GmbH (Wesseling, Germany). This printer features a precision module (PSK-090-NN-1, length: 340 mm) with a maximum stroke of 150 mm, supplied by Bosch Rexroth. The system is equipped with two force sensors from GTM Testing and

Measuring Technology GmbH. The drive configuration, designated as 99 (01) KGT 16x5/T5/C2, includes a 5 mm pitch ball screw designed to eliminate backlash and ensure increased preload. This setup adheres to precision class T5 standards, achieving an accuracy of 23 μm per 300 mm. It is powered by a Rexroth servo motor (MS2N04-B0BTN-CMSH1-NNNNE-NN), which includes a holding brake and is streamlined by a single cable connection. Control and power management are handled by Rexroth IndraDrive drive controllers and HCS01 power units (COMPACT INVERTER HCS01.1E-W0013-A-02-B-ET-EC-EP-NN-NN-FW). A computer-aided design (CAD) drawing of the device is shown in previous publication.[5]

Fluorescence Microscopy. Microscopy analysis was performed on the instrument from Leica Microsystems (DMi8). A dry objective of HCX PL FLUOTAR 20x/0.50 and 40x/0.80 were used. Images were analyzed on ImageJ (https://imagej.org) v1.54.

analyzed

Image analysis. Images of patterned saccharide modified substrates were first subtracted (rolling ball radius = 20 pixels) and brightness was corrected. A cropped part of the images was then selected (412 × 532 pixels, imaged at 20x magnification) for all sugar except sorbose and fucose (412 ×1064 pixels) as they were imaged with 40x magnification. to create **Figure 5**. The normalized intensity plots were prepared by selecting a selected area of the images on imageJ and the corresponding intensity data were collected by using plot profile function. The obtained intensity data were normalized and plotted against the distance. Image sizes of 397 × 335 pixels and 476 × 414 pixels were used in **Figure S64** and **S65** respectively.

The cell image channels (transmission and fluorescence) were first split and cropped. Positive control (316.33 × 307.35 microns; cropped = 954 × 1071 pixels) and Triton-X treated negative control ($183.03 \times 166.19\ microns$; $1141 \times 1036\ pixels$) images were then background-subtracted *via* rolling ball method (rolling ball radius of 50 pixels and 20 pixels, respectively).

Printing images of patterned Ac_2O treated cell membrane (original = 2048 × 2048, cropped = 1251 × 1029 pixels) were initially recorded as Z-stack and max. intensity projections were obtained on image J.

These images, along with fluorescence images of pH 4 washed printed substrate (original = 2048 × 2048, cropped = 1225 × 1007 pixels) and the negative control (printed with bare stamp without grafted polymer; 2048 × 2048 pixels) were background subtracted (rolling ball radius of 80, 20, and 50 respectively).

Image sizes of (370 × 318 pixels) were used in **Figure S71**. The Z-stack of microgel images (2048 × 2048 pixels, cropped) were first split into transmission and fluorescence channels, and min. intensity projections were obtained. Brightness and contrast were subsequently corrected.

For **Figure 7 c,** a gamma correction (value = 2) was applied, and background subtraction was performed (rolling ball radius = 10 pixels). The MPL inferno heat map was selected from the lookup table on image J. **Figure S69 b** was first background-subtracted (rolling ball radius = 20 pixels) and stitched. To mention, all images are brightness- and contrast-corrected. All separate microscopy channels for cell images are displayed in grey scale, except for the merged images of all channels.

Confocal Microscopy. Confocal microscopy analyses were performed on the instrument from Leica SP8 Confocal Microscopy (Leica Microsystems Inc., Heidelberg, Germany). Confocal microscopy was carried out at 40x magnification with FITC-Dextran attached particles placed on concave microscope slides covered with a cover slip. Dye excitation was accomplished using a 488 nm (FITC) helium/neon (He–Ne) laser.

2. Materials and Methods

Materials. The commercially available standard kit of SYLGARD 184 from Dow Corning was used for the PDMS preparation. 2,3:4,5-Di-*O*-isopropylidene-*β*-*D*-fructopyranose (> 98%), Trifluoromethanesulfonic anhydride, methylcatechol, trifluoromethanesulfonic anhydride, 4-Cyano-4-[[(dodecylthio)carbonothioyl]thio]pentanoic acid, 3-aminopropyl(triethoxy)silane (APTES, > 99.8%) were purchased from TCI Europe. AIBN was purchased from Sigma Aldrich. *L*-(-)-sorbose (≥ 98%), *D*-(+)-galactose (≥ 98%), *D*-(+)-glucose (≥ 99.5%, anhydrous, sum of enantiomers), Alizarin Red S were purchased from Carl Roth. *L*-fucose was purchased from Biosynth. Boron trifluoride etherate ($BF_3.Et_2O$), chlorotrimethylsilane, *N*-acryloyl morpholine (NAM), triethylamine, methacrylic anhydride, triphenylphosphine, trifluoroacetic acid, pyridine, potassium monobasic and dibasic phosphates, benzoxaborole (BOB), succinic anhydride, 4-Dimethylaminopyridine (DMAP, 99%) were purchased from Sigma Aldrich. Dopamine hydrochloride (DA·HCl, 99%), sodium tetraborate decahydrate ($Na_2B_4O_7 \cdot 10H_2O$, Borax, +99%), sodium azide (NaN_3) (> 99%), and sodium methoxide (NaOMe, 98%) were bought from Alfa Aesar. *N*,*N'*-Dicyclohexylcarbodiimide (DCC) was purchased from Roth (Germany). 6-aminobenzo[c][1,2]oxaborol-1(3H)-ol (ABOB) were purchased from BLDPharma (98%). Poly(styrene)-*b*-poly(acrylic acid) (PS-*b*-PAA) (PS:PAA 3000:5000, Đ ≤ 1.1) was purchased from Sigma Aldrich. Fluorescently labelled dextran (40k) was purchased from Sigma Aldrich. The inhibitor from NAM was removed by passing through activated alumina from Merck (neutral, Brockmann I). *N*, *N*-dimethylformamide (DMF) and ethyl acetate were obtained from VWR. Hexane and dichloromethane were purchased from Th. Geyer. Plasma treatment was carried out using PlasmaFlecto10. Gastric epithelial cells and epithelial cell medium were obtained from Innoprot (Derio, Spain). 4',6-diamidino-2-phenylindole (DAPI) was obtained from Roth (Karlsruhe, Germany). Poly-*L*-lysine was obtained from ScienCell (Carlsbad, USA), $PBS^{+/+}$ buffer for cell treatment was purchased from Biochrom GmbH (Berlin, Germany). $PBS^{-/-}$ were obtained from Sigma Aldrich (St. Louis, USA). Formaldehyde for cell fixation was from Carl Roth (Roth, Germany). Triton X-100 solution was bought from VWR (Darmstadt, Germany). Culture and staining of mouse fibroblasts L929 cells were purchased from Leibnitz Institute DSMZ (Braunschweig, Germany). Minimum essential medium (MEM) was bought from Gibco (Schwerte, Deutschland). Fetal bovine serum was obtained from PAN (Aidenbach, Germany).

2.1 Monomer preparation for grafting from the stamp surface

Synthesis of dopamine methacrylamide (DMA). The synthesis was performed following the literature procedure.[6] Sodium tetraborate ($Na_2B_4O_7$, 4 g, 10.48 mmol)) and Na_2CO_3 (1.6 g, 15.1 mmol) were dissolved in 40 mL water and deoxygenated with nitrogen gas. Dopamine.HCl (2 g, 10 mmol) was added. Methacrylic anhydride (1.86 g, 12.56 mmol) was dissolved in 10 mL THF and added dropwise at 0 °C. The mixture was stirred for 24 hours. The pH of the reaction solution was brought down to < 2 with 1M HCl solution and extracted with ethyl acetate (100 mL) and dried over $MgSO_4$. The solvent was reduced to approx. 30 mL and precipitated in cold hexane (400 mL). The mixture was kept in the freezer for precipitation. The precipitation was filtered and precipitated again in 400 mL hexane. The collected grey precipitate was dried under *vacuo*. The yield was 74%.

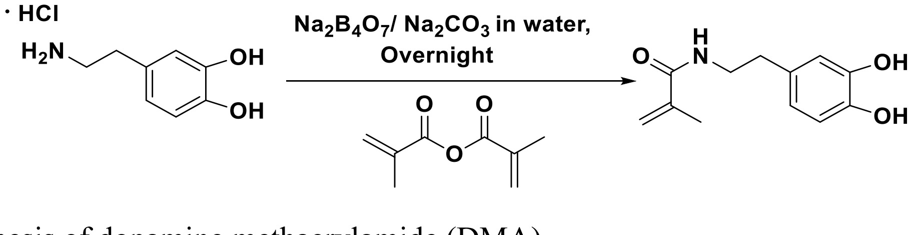

Figure S1. Synthesis of dopamine methacrylamide (DMA).

^1H NMR (400 MHz, DMSO-d_6) δ 7.92 (q, J = 4.8, 4.0 Hz, 1H), 6.65 – 6.56 (m, 2H), 6.43 (dd, J = 7.9, 2.1 Hz, 1H), 5.61 (t, J = 1.3 Hz, 1H), 5.30 (p, J = 1.6 Hz, 1H), 3.23 (ddd, J = 9.1, 7.6, 5.9 Hz, 2H), 2.55 (dd, J = 8.7, 6.5 Hz, 2H), 1.84 (q, J = 1.7, 1.2 Hz, 3H). ^{13}C NMR (101 MHz, DMSO-d_6) δ 167.31, 145.05, 143.50, 140.09, 130.29, 119.20, 118.80, 115.97, 115.47, 40.96, 34.61, 18.66.

Hydroxy group protected dopamine methacrylamide. The reaction was carried out according to the literature.[7] The protection of DMA was performed by dissolving 300 mg (1.35 mmol) of DMA in a mixture of 2 mL ethanol and 4 mL Milli-Q water. This solution was then added to 10 mL of a degassed aqueous solution containing 1.10 g (2.88 mmol) of borax, which is twice the molar equivalent of the catechol moieties. The reaction mixture was stirred continuously under nitrogen for one hour. Following this, the product was freeze-dried and used without any further purification.

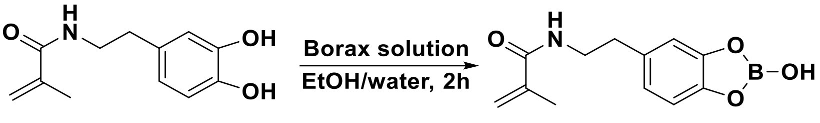

Figure S2. Catechol protection of dopamine methacrylamide (DMA-p).

^1H NMR (400 MHz, D_2O) δ 6.64 – 6.50 (m, 3H), 5.58 (s, 1H), 5.39 (d, J = 2.0 Hz, 1H), 3.45 (t, J = 6.8 Hz, 2H), 2.72 (t, J = 6.8 Hz, 2H), 1.87 (s, 3H).^{11}B NMR (128 MHz, D_2O) δ 12.59 (s), 7.85 (s).

2.2 Fabrication of stamp for grafting

PDMS stamp preparation and CTA attachment. SYLGARD 184 PDMS prepolymer and curing agent (10:1 ratio) was poured onto a Si master (fixed in between 2 glass plates) with 4 µm stripe patterns. The mixture was cured to obtain a patterned PDMS sheet. The PDMS sheet was washed with DCM using a Soxhlet extractor for approimately 8 h and dried under vacuum at 40 °C for 1 h. The sheet was cut into a 1 cm^2 piece to be used as a stamp. The stamp was plasma treated to activate the surface (100 W, 60s, 100 air). The plasma-treated stamp was kept in a PFA chamber along with 2 vials containing 300 µL APTES and 300 µL NH_4OH solution (30% aqueous solution of NH_3) and heated at 70 °C for 2 h for APTES functionalization *via* CVD. A solution of 4-Cyano-4-[[(dodecylthio)carbonothioyl]thio]pentanoic acid (CTA, 10 mg, 0.0248 mmol), DCC (10 mg, 0.048 mmol), and TEA (10 µL, 0.00726 mg, 0.072 mmol) was prepared in 1 mL DMF. APTES functionalized stamp was covered with this solution overnight for CTA attachment to the surface. The stamp was then washed with Milli-Q water, rinsed with EtOH, and dried with soft air stream.

Surface grafted poly(90%*N*-acryloylmorpholine-*co*-10%dopamine methacrylamide). The polymerization technique was followed by previous literature procedure.[7] The inhibitor was removed from NAM by passing it through alumina. Freeze-dried DMA-p (containing *ca.* 10 mg of DMA-p in the mixture, 0.05 mmol) from the previous step was dissolved in Milli-Q water (0.933 mL). For a targeted 10% of the total composition (approximately), a rough calculation of DMA's quantity in the protected DMA mixture was done. NAM (64 mg, 0.45 mmol) was added to it. An initiator-to-CTA ratio of 5 to 1 was applied. 4-Cyano-4-[[(dodecylthio)carbonothioyl]thio]pentanoic acid (0.4 mg, 0.0010 mmol) and AIBN (0.821 mg, 0.0050 mmol) were taken from their respective stock solution in DMF and added to monomer solution. A final 1 mL solution was prepared. The stamp was immersed in the solution and degassed with N_2 for 30 min. Polymerization reaction was carried out at 70 °C for 18 h. After the reaction the stamp was washed with Milli-Q water. To deprotect the catechol hydroxy functionality the stamp was stirred for 1 h in HCl solution (1M) prior to inking and printing. Polymers were also formed in the solution which was analyzed via ^1H NMR. Polymer formed in the solution was also purified after borax deprotection with HCl solution (pH 1) via dialysis using a 3.5 kDa dialysis tube (Spectra/Por, Spectrum Laboratories, Inc). ^1H NMR and SEC data were measured afterwards.

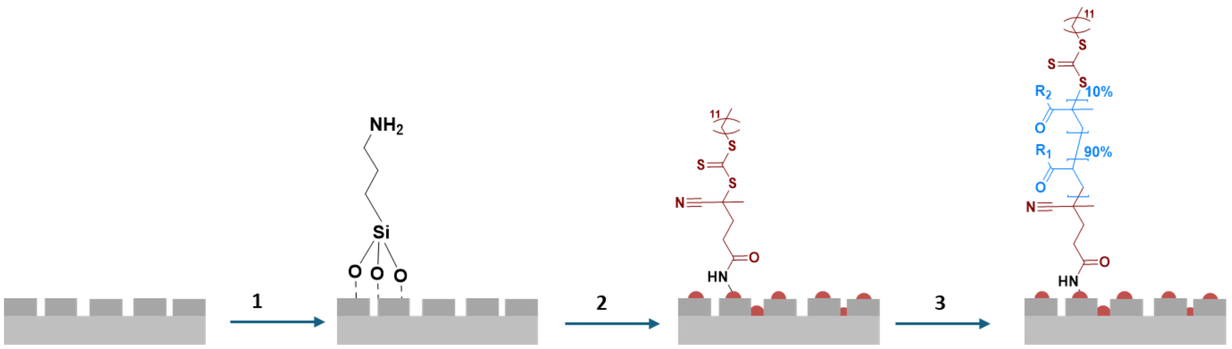

Figure S3. Polymer grafting from the PDMS stamp surface. 1: APTES CVD, 2: CTA attachment, 3: Grafting from using RAFT polymerization.

2.3 Syntheses of monomers with monosaccharides (sugar monomers)

2.3.1 Ketose monomers (sorbose derivatives):

Synthesis of 2,3:4,6-Di-*O*-isopropylidene-*L*-sorbose (DIS, **1**). The synthesis was done according to literature.[8] Iodine (0.7 g, 2.765 mmol) was added to a dry acetone solution (500 mL) of *L*-sorbose (3 g, 16.68 mmol) and stirred at room temperature overnight. The reaction was monitored *via* TLC. 50 mL excess saturated aqueous $Na_2S_2O_3$ was added dropwise and stirred for 20 min. The acetone was evaporated, and the aqueous layer was extracted with DCM (3x 50 mL). The organic phases were combined and dried over Na_2SO4. The solvent was removed to obtain a crude residue. The residue was purified via flash chromatography (Hexane/EtOAc 1:1). A colorless oil was obtained (**1**) with a yield of (3.2 g, 74 %).

^{1}H NMR (400 MHz, $CDCl_3$) δ: 4.47 (s, 1H), 4.32 (d, J = 2.2 Hz, 1H), 4.09 (q, J = 2.2 Hz, 1H), 4.07 (dd, J = 3.6, 2.0 Hz, 2H), 3.87 (d, J = 11.9 Hz, 1H), 3.79 (d, J = 11.8 Hz, 1H), 1.50 (s, 3H), 1.43 (s, 3H), 1.36 (s, 6H). ^{13}C NMR (101 MHz, chloroform-d) δ 114.29, 111.98, 97.57, 84.96, 73.29, 72.25, 63.66, 60.28, 28.94, 27.34, 26.49, 18.58. MS (ESI-positive): calculated for $C_{12}H_{20}O_6H$ $[M + H]^+$, 261.1372; found 261.1326; calculated for $C_{12}H_{20}O_6Na$ $[M + Na]^+$, 283.1192; found 282.2758, 283.2852; calculated for ($2\times C_6H_{12}O_6$), 360.1267; found 359.1006, 360.1135.

Synthesis of 2,3:4,6-Di-*O*-isopropylidene-*L*-sorbose trifluoromethanesulfonate (DIS-OTf, **3**). A similar approach to the literature was taken to synthesize the product **3**.[9] DIS (**1,** 1 eq, 3 g, 11.51 mmol) was dissolved in 50 mL DCM. The solution was cooled down to -10 °C using a NaCl/ice bath under N_2 flow. Pyridine (6 eq, 5.6 mL, 69 mmol) was added to the solution. Trifluoromethanesulfonic anhydride (3 eq, 5.66 mL, 34.5 mmol) was added dropwise to the solution. The yellow solution was stirred for 4h at -10 °C. The reaction was monitored via TLC. The solution was then washed with water and extracted with DCM (4x, 30 mL). The product was purified with column chromatography (4:1, Hex:EtOAc). Yield = 3.80 g (10.04 mmol, 87%). TLC plates were stained with 0.1% napthoresorcinol solution and 1% H_2SO_4 solution in EtOH, followed by drying with a heat gun.

^{1}H NMR (400 MHz, $CDCl_3$) δ: 4.47 (s, 1H), 4.32 (d, J = 2.2 Hz, 1H), 4.09 (q, J = 2.2 Hz, 1H), 4.07 (dd, J = 3.6, 2.0 Hz, 2H), 3.87 (d, J = 11.9 Hz, 1H), 3.79 (d, J = 11.8 Hz, 1H), 1.50 (s, 3H), 1.43 (s, 3H), 1.36 (s, 6H). ^{19}F NMR (377 MHz, $CDCl_3$) δ -74.45 (s). ^{13}C NMR (101 MHz, Chloroform-*d*) δ 113.67, 110.97, 97.58, 85.74, 84.15, 80.23, 76.86, 73.27, 73.21, 72.79, 72.39, 61.43, 60.14, 30.94, 28.84, 27.48, 27.35, 26.22, 26.19, 18.56. MS (ESI-positve): calculated for $C_{13}H_{19}F_3O_8SH$ [M], 392.0752, found 392.9154; calculated for $C_{13}H_{19}F_3O_8SH$ $[M + H]^+$, 393.0831 ; found 393.0669; 392.9154, found 490.8934, calculated for ($2\times C_6H_{12}O_6$), 360.1267; found 359.1075

Synthesis of 2,3:4,6-Di-*O*-isopropylidene-*L*-sorbose azide (DIS-N_3, **5**). Literature procedure was followed.[9] 3.5 g (8.462 mmol, 1 eq.) **3** was dissolved in 180 mL DMF. NaN_3 (13.33 mmol, 1.3 eq) was added to the solution and the reaction was heated at 55 °C overnight. The reaction cooled down to room

temperature and DMF was removed. The crude product was dissolved in EtOAc and washed with water (3x). The organic phase was then washed with brine (2x) and dried over $MgSO_4$. The yellow oil solidified under vacuum drying. The solid was redissolved in DCM and the solvent slowly evaporated in the fume hood through a needle. Yield = 87% (2.1 g, 7.36 mmol).

^{1}H NMR (400 MHz, $CDCl_3$) δ 4.41 (s, 1H), 4.32 (d, *J* = 2.4 Hz, 1H), 4.12 (q, *J* = 2.0 Hz, 1H), 4.08 – 3.96 (m, 2H), 3.71 (d, *J* = 13.1 Hz, 1H), 3.40 (d, *J* = 13.2 Hz, 1H), 1.52 (s, 3H), 1.46 (s, 3H), 1.42 (s, 3H), 1.36 (s, 3H). ^{13}C NMR (101 MHz, Chloroform-*d*) δ 114.07, 112.90, 97.40, 84.55, 73.04, 72.72, 60.30, 53.37, 28.90, 27.59, 26.17, 18.63. MS (ESI-positive): calculated for $C_{12}H_{19}N_3O_5H$ $[M + H]^+$, 286.1403; 286.1404; found 282.2785; found 294.9385; calculated for $C_{13}H_{19}F_3O_8S$, 392.0753; calculated for $C_{16}H_{25}N_4O_5K$ $[M + 2CH_3CN + K]^+$, 392.1462 ; found 392.9106

Synthesis of 2,3:4,6-Di-*O*-isopropylidene-*L*-sorbose amine (DIS-NH_2, **7**). Synthetic procedure to reduce azide to amine was taken from the literature and applied here.[10] DIS-N_3 (**5**, 1.9 g, 6.66 mmol, 1 eq.) and triphenylphosphine (1.97 g, 7.34 mmol, 1.1 eq.) were dissolved in 15 mL DMF together in a round bottom flask. The solution was left stirred overnight at room temperature under nitrogen. Then, Milli-Q water (10 mL) and additional DMF (8 mL) were added to the solution and the reaction was carried out for another 2h at 90 °C. With the addition of water precipitation of a white solid was seen which dissolved slowly at higher temperature. The reaction was cooled down afterwards at room temperature and the solvent was removed. The yellow oil was redissolved in EtOAc and washed with water (3x) and then with brine. The organic phase was collected, and the solvent was evaporated. TLC shows 3 spots that refer to triphenylphosphine oxide, unreacted triphenylphosphine and the product in the middle. Column chromatography was performed (Hex/EtOAc/Et_3N 30:10:1). TLC plates were stained with 0.1% napthoresorcinol solution and 1% H_2SO_4 solution in EtOH, followed by drying with a heat gun. Note, that the by-product triphenylphosphine oxide could not be removed entirely during column chromatography, as monitored by ^{1}H NMR (signals at 7.69 to 7.42 ppm). The exact percentage could not be calculated as it could not be purified entirely.

^{1}H NMR (400 MHz, $CDCl_3$) δ 7.69 – 7.62 (m, 6H), 7.56 – 7.42 (m, 9H), 4.35 – 4.25 (m, 2H), 4.14 – 3.98 (m, 3H), 3.06 (d, *J* = 5.5 Hz, 2H), 1.49 (s, 3H), 1.43 – 1.39 (m, 3H), 1.36 (dd, *J* = 10.2, 4.1 Hz, 6H). ^{13}C NMR (101 MHz, Chloroform-*d*) δ 133.01, 131.95 (t), 128.46 (d), 115.32, 111,38, 97. 39, 85.34, 73.44, 72.12, 60.41, 47.10, 28.95, 27.41, 26.67, 18.68. MS (ESI-positive): calculated for $C_{12}H_{21}NO_5Na$ $[M + Na]^+$, 282.1317; found 282.2838; calculated for $C_{12}H_{21}NO_5NH_4$ $[M + NH_4]^+$, 277.1763; found 279.0993; found 294.9385; calculated for $C_{13}H_{19}F_3O_8S$, 392.0753; $C_{16}H_{25}N_4O_5K$ [**DIS-N$_3$** + $2CH_3CN$ + K]$^+$, 392.1462; found 392.9105

Synthesis of 2,3:4,6-Di-*O*-isopropylidene-*L*-sorbose methacrylamide (DISMAm, **9, M1**). DIS-NH_2 (**7**) (3.8 g unpurified mixture) was dissolved in DCM (20 mL). Triethylamine (1.3 mL) was added to the solution. Methacrylic anhydride (MMA, 1.4 mL) was then added dropwise to the solution at 0 °C. The reaction was stirred at room temperature for 20 h. The reaction mixture was washed with water (2 x). The organic phase was collected and purified by column chromatography (Hex/EtOAc 1:1). The yield was 1 g. Exact yield percentage could not be calculated as the starting amine could not be purified.

^{1}H NMR (400 MHz, DMSO-*d$_6$*) δ 5.69 (t, *J* = 1.2 Hz, 1H), 5.33 (p, *J* = 1.6 Hz, 1H), 4.48 (s, 1H), 4.26 (d, *J* = 2.3 Hz, 1H), 4.03 – 4.00 (m, 1H), 3.96 (q, *J* = 2.0 Hz, 1H), 3.83 (d, *J* = 13.4 Hz, 1H), 3.65 (dd, *J* = 14.0, 7.0 Hz, 1H), 3.46 (dd, *J* = 14.1, 5.6 Hz, 1H), 1.84 (q, *J* = 2.0, 1.6 Hz, 3H), 1.38 (d, *J* = 1.3 Hz, 6H), 1.24 (d, *J* = 1.7 Hz, 6H). ^{13}C NMR (101 MHz, Chloroform-*d*) δ 169.21, 140.50, 119.19, 113.29, 111.41, 97.67, 85.33, 73.90, 71.82, 60.11, 44.11, 28.80, 27.01, 26.21, 18.75, 18.53, 18.11. MS (ESI): calculated for $C_{16}H_{25}NO_6H$ $[M + H]^+$, 328.1772; found 328.1748; calculated for $C_{18}H_{28}N_2O_6Na$ [M + CH_3CN +

Na]$^+$, 391.1845; calculated for $C_{13}H_{19}F_3O_8S$, 392.0753; found 392.9109; also found 424.1831; and 482.2104.

2.3.2 Ketose monomers (fructose derivatives)

Synthesis of 2,3:4,5-Di-*O*-isopropylidene-*β*-*D*-fructopyranose trifluoromethanesulfonate (DIF-OTf, **4**). Literature procedure was followed in analogy to the synthesis of **4.**[9] Commercially obtained 2,3:4,5-Di-O-isopropylidene-β-D-fructopyranose (DIF, **2**) was used. The synthesis protocol was the same as **3**. The product was purified with column chromatography (4:1, Hex:EtOAc). The obtained product **4** had a Yield of 3.88 g (10.26 mmol, 89%). TLC plates were stained with 0.1% napthoresorcinol solution and 1% H_2SO_4 solution in EtOH, followed by drying with heat gun.

^{1}H NMR (400 MHz, $CDCl_3$) δ 4.64 (dd, *J* = 7.9, 2.6 Hz, 1H), 4.52 (d, *J* = 10.5 Hz, 1H), 4.40 (d, *J* = 10.5 Hz, 1H), 4.34 – 4.22 (m, 2H), 3.92 (dd, *J* = 13.0, 1.9 Hz, 1H), 3.79 (dd, *J* = 13.0, 0.8 Hz, 1H), 1.47 (s, 3H), 1.40 (s, 3H), 1.35 (s, 3H). ^{19}F NMR (377 MHz, $CDCl_3$) δ -74.39. ^{13}C NMR (101 MHz, Chloroform-*d*) δ 146.65, 109.83, 109.30, 109.07, 108.53, 77.72, 76.36, 70.98, 69.70, 65.51, 61.62, 25.76, 25.74, 25.34, 25.03, 23.94. MS (ESI-positive): calculated for $C_{13}H_{19}F_3O_8SH$ [M + H]$^+$ 393.0831; found 393.0822; found 392.0752; found 392.9154; also found 490.8916; and 491.0569.

Synthesis of 2,3:4,5-Di-*O*-isopropylidene-*β*-*D*-fructopyranose azide (DIF-N_3, **6**). DIF-OTf (**4,** 3.5 g, 8.462 mmol) was dissolved in DMF and heated at 55 °C overnight. Similar literature procedure[9] was used to synthesize **6** with a yield of 88% (2.13 g).

^{1}H NMR (400 MHz, $CDCl_3$) δ 4.60 (dd, *J* = 7.9, 2.7 Hz, 1H), 4.30 – 4.19 (m, 2H), 3.91 (dd, *J* = 13.0, 1.9 Hz, 1H), 3.76 (dd, *J* = 13.0, 0.8 Hz, 1H), 3.58 (d, *J* = 13.0 Hz, 1H), 3.27 (d, *J* = 13.0 Hz, 1H), 1.55 (s, 3H), 1.47 (d, *J* = 8.0 Hz, 6H), 1.34 (s, 3H). ^{13}C NMR (101 MHz, Chloroform-*d*) δ 109.18, 109.09, 102.65, 76.52, 76.84, 70.76, 70.06, 61.51, 55.52, 26.58, 25.83, 24.77, 24.00. MS (ESI-positive): calculated for $C_{12}H_{19}N_3O_5H$ [M + H]$^+$, 286.1403; found 286.1410; found 282.2805; found 294.9385; calculated for $C_{13}H_{19}F_3O_8S$, 392.0753; found 384.1139; calculated for $C_{16}H_{25}N_4O_5K$ [M + 2CH_3CN + K]$^+$, 392.1462; found 392.9108.

Synthesis of 2,3:4,5-Di-*O*-isopropylidene-*β*-*D*-fructopyranose amine (DIF-NH_2, **8**). Similar synthetic procedure as **7** was followed.[10] Initially DIF-N_3 (**6**, 1.95 g, 6.84 mmol, 1 eq.) and triphenylphosphine (2.02 g, 7.52 mmol, 1.1 eq.) were dissolved in 15 mL DMF together in a round bottom flask. The crude product was purified by column chromatography (Hex/EtOAc/Et_3N 30:10:1). A yellow oil with a yield of 77% (1.37 g, 5.267 mmol). TLC plates were stained with 0.1% napthoresorcinol solution and 1% H_2SO_4 solution in EtOH, followed by drying with a heat gun.

^{1}H NMR (400 MHz, $CDCl_3$) δ 4.57 (dd, *J* = 7.9, 2.4 Hz, 1H), 4.26 – 4.20 (m, 1H), 4.18 (d, *J* = 2.4 Hz, 1H), 3.91 – 3.83 (m, 1H), 3.76 (d, *J* = 13.0 Hz, 1H), 2.99 – 2.84 (m, 2H), 1.52 (d, *J* = 5.6 Hz, 3H), 1.47 (s, 3H), 1.37 (s, 3H), 1.34 (s, 3H). ^{13}C NMR (101 MHz, Chloroform-*d*) δ 108.84, 107.77, 103.86, 77.04, 76.73, 71.94, 70.81, 70.24, 60.98, 49.88, 26.43, 25.78, 25.34, 23.91. MS (ESI-positive): calculated for $C_{12}H_{21}NO_5H$ [M + H]$^+$, 260.1498; found 260.1485, calculated for $C_{12}H_{21}NO_5Na$ [M + Na]$^+$, 282.1317; found 282.2812; calculated for $C_{12}H_{21}NO_5NH_4$ [M + NH_4]$^+$, 277.1763; found 279.0973; calculated for $C_{14}H_{24}N_2O_5$ [M + CH_3CN + H]$^+$, 301.1438; found 302.1670; calculated for $C_{13}H_{19}F_3O_8S$, 392.0753; and $C_{16}H_{25}N_4O_5K$ [**DIS-N_3** + 2CH_3CN + K]$^+$, 392.1462; found 392.9109

Synthesis of 2,3:4,5-Di-*O*-isopropylidene-*β*-*D*-fructopyranose methacrylamide (DIFMAm, **10, M2**) DIF-NH_2 (**8**) (1.3 g, 4.99 mmol) was dissolved in DCM (10 mL). Triethylamine (1.1 eq, 0.765 mL) was added to the solution. Methacrylic anhydride (MMA, 1.2 eq, 0.9 mL) was then added dropwise to the solution at 0 °C. The reaction was stirred at room temperature for 20 h. The reaction mixture was washed

with water (2 x). The organic phase was collected and purified by column chromatography (Hex/EtOAc 4:1). The yield was 1.2 g (73.5%).

^{1}H NMR (400 MHz, $CDCl_3$) δ 5.66 (q, *J* = 2.1, 1.6 Hz, 1H), 5.32 (p, *J* = 1.5 Hz, 1H), 4.57 (dt, *J* = 7.8, 2.8 Hz, 1H), 4.28 (d, *J* = 2.6 Hz, 1H), 4.21 (dd, *J* = 7.8, 2.0 Hz, 1H), 3.91 – 3.85 (m, 1H), 3.78 – 3.71 (m, 2H), 3.58 (dd, *J* = 13.9, 5.3 Hz, 1H), 1.98 – 1.95 (m, 3H), 1.53 (d, *J* = 1.4 Hz, 3H), 1.48 (s, 3H), 1.39 (s, 3H), 1.34 (d, *J* = 3.3 Hz, 3H). ^{13}C NMR (101 MHz, Chloroform-*d*) δ 168.91, 140.43, 119.19, 109.06, 108.47, 102.69, 71.90, 70.89, 70.82, 70.30, 61.62, 46.86, 26.28, 25.98, 23.96, 18.79. MS (ESI-positive): calculated for $C_{16}H_{25}NO_6H$ $[M + H]^+$, 328.1772; found 328.1751; calculated for $C_{18}H_{28}N_2O_6Na$ $[M + CH_3CN + Na]^+$, 391.1845; calculated for $C_{13}H_{19}F_3O_8S$, 392.0753; found 392.9089, also found 424.1886; 482.2327; and 490.886.

2.3.2 Aldose monomers

Synthesis of 1,2,3,4,6-Penta-*O*-acetyl-*β-D*-glucose (11), 1,2,3,4,6-Penta-*O*-acetyl-*β-D*-galactose (12), and 1,2,3,4-tetra-*O*-acetyl-*α-L*-fucose (13). The syntheses were performed following a literature procedure to obtain the products.[11] For example, *L*-fucose (5 g, 30.4 mmol) were weighed. Pyridine (0.5 M) was added. Acetic anhydride (20 mL, 211 mmol, 6.94 eq.) was added dropwise over 20 min. The reaction was stirred for 3 d at room temperature. The mixture was then evaporated with toluene and a column chromatography of the crude mixture was performed to purify the product (Hex/EtOAc, 1:1).

^{1}H NMR (400 MHz, Chloroform-d, **11**) δ 6.32 (d, J = 3.7 Hz, 1H), 5.46 (t, J = 9.9 Hz, 1H), 5.19 – 5.04 (m, 2H), 4.32 – 4.20 (m, 1H), 4.14 – 3.98 (m, 2H), 2.17 (d, J = 1.0 Hz, 3H), 2.08 (d, J = 1.1 Hz, 3H), 2.05 – 2.00 (m, 9H). ^{13}C NMR (101 MHz, Chloroform-d, **11**) δ 170.44, 170.03, 169.46, 169.20, 168.56, 88.87, 69.62, 68.99, 67.68, 61.25, 21.79 – 19.11 (m). MS (ESI-positive, **11**): calculated for $C_{16}H_{22}O_{11}H$ $[M + H]^+$, 391.1240; found 392.9154; calculated for $C_{14}H_{20}O_9$ (FucoseOAc, **13**), 332.1107; found 332.1114, 331.0984.

^{1}H NMR (400 MHz, Chloroform-d, **12**) δ 6.36 (d, J = 2.1 Hz, 1H), 5.48 (d, J = 1.6 Hz, 1H), 5.32 (d, J = 2.1 Hz, 2H), 4.33 (t, J = 6.9 Hz, 1H), 4.11 – 4.00 (m, 2H), 2.14 (d, J = 1.4 Hz, 6H), 2.02 (s, 3H), 1.99 (d, J = 6.6 Hz, 6H). ^{13}C NMR (101 MHz, Chloroform-d, **12**) δ 170.35 (d, J = 2.5 Hz), 170.13 (d, J = 1.6 Hz), 169.87, 168.92, 89.71, 68.75, 67.38 (d, J = 6.0 Hz), 66.43, 61.24, 21.77 – 19.53 (m). MS (ESI-positive, **12**): calculated for $C_{16}H_{22}O_{11}H$ $[M + H]^+$, 391.1240; found 392.9154; calculated for $C_{14}H_{20}O_9$ (FucoseOAc, **13**), 332.1107; found 331.0809; found 332.0981.

^{1}H NMR (400 MHz, Chloroform-d, **13**) δ 6.34 – 6.28 (m, 1H), 5.35 – 5.26 (m, 3H), 4.25 (q, J = 6.5 Hz, 1H), 2.18 – 2.11 (m, 6H), 1.98 (td, J = 4.7, 1.6 Hz, 6H). ^{13}C NMR (101 MHz, Chloroform-d, **13**) δ 170.54, 170.19, 169.95, 169.15, 89.95, 70.58, 67.87 (d, J = 9.5 Hz), 67.28, 66.47, 21.14 – 20.46 (m), 15.92. MS (ESI-positive, **13**): calculated for $C_{14}H_{20}O_9K$ $[M + K]^+$, 371.0744; found 371.0723; calculated for $C_{18}H_{26}N_2O_9Na$ $[M + 2CH_3CN + Na]^+$, 437.1536; $C_{18}H_{26}N_2O_9Na$ $[M + 2CH_3CN + NH_4]^+$, 432.1982; found 431.0906.

Synthesis of 2,3,4,6-Tetra-*O*-acetyl-*β-D*-glucosylethyl acrylamide (GlucosEAm, **14, M3**) was prepared according to a literature protocol.[12] Accordingly, 1,2,3,4,6-Penta-*O*-acetyl-*β-D*-glucose (1.1 g, 3 mmol) was dissolved in DCM (20 mL), and *N*-(2-hydroxyethyl) acrylamide (0.668 g, 5.8 mmol) was added dropwise under a nitrogen atmosphere. The solution was cooled in an ice bath, followed by the slow addition of boron trifluoride diethyl etherate ($BF_3{\cdot}Et_2O$, 2.62 mL, 21.2 mmol) over 10 min. The reaction was kept in the ice bath for 2 h before allowing it to warm to room temperature. The conversion was checked *via* TLC (hexane/EtOAc 1:3). The reaction was run for 3 days. The reaction mixture was poured into ice water and extracted twice. The aqueous layer was extracted once again with DCM. The combined organic layers were washed (2x) with saturated $NaHCO_3$ solution and with brine (1x). The organic layer was then dried over Na_2SO_4 and concentrated under vacuum. The crude product (yellow oil) was purified by silica gel

column chromatography (hexane/EtOAc 1:3). The final product was pale yellow gum (yield = 0.25 g, 19%). TLC plates were stained with 0.1% napthoresorcinol solution and 1% H_2SO_4 solution in EtOH, followed by drying with a heat gun.

^{1}H NMR (400 MHz, $CDCl_3$) δ 6.30 (d, J = 16.8 Hz, 1H), 6.11 (ddd, J = 16.7, 10.1, 5.7 Hz, 2H), 5.66 (t, J = 9.1 Hz, 1H), 5.21 (t, J = 9.5 Hz, 1H), 5.07 (t, J = 9.5 Hz, 1H), 4.99 (t, J = 8.6 Hz, 1H), 4.51 (d, J = 7.9 Hz, 1H), 4.27 – 4.07 (m, 2H), 3.92 – 3.46 (m, 5H), 2.14 – 1.99 (m, 12H). ^{13}C NMR (101 MHz, Chloroform-d) δ 171.70 – 168.61 (m), 165.70 (d, J = 12.5 Hz), 130.61 (d, J = 11.4 Hz), 126.72 (d, J = 17.6 Hz), 100.96, 72.74 – 70.94 (m), 68.72 (d, J = 96.0 Hz), 63.16, 61.81, 39.01 (d, J = 44.9 Hz), 21.20 – 20.12 (m). MS (ESI-positive): calculated for $C_{19}H_{27}NO_{11}H$ $[M + H]^+$, 446.1662; found 446.1675; calculated for $C_{21}H_{30}N_2O_{11}Na$ $[M + CH_3CN + Na]^+$, 509.1747; calculated for $[M + CH_3CN + NH_4]^+$, 504.2193; found 506.5334.

Synthesis of 2,3,4,6-Tetra-*O*-acetyl-*β*-*D*-galactosylethyl acrylamide (GalactosEAm, **15, M4**). Identical literature procedure was followed.[12] The reaction was carried exactly like **14**. The final product was pale yellow gum after column chromatography with Hexane/EtOAc (1:3) (yield = 0.67 g, 50%). The analytical data also resemble the literature.[11]

^{1}H NMR (400 MHz, $CDCl_3$) δ 6.33 – 6.26 (m, 1H), 6.10 (ddd, *J* = 17.0, 10.3, 3.2 Hz, 2H), 5.66 (ddd, *J* = 10.2, 2.7, 1.5 Hz, 1H), 5.39 (dd, *J* = 3.5, 1.2 Hz, 1H), 5.18 (dd, *J* = 10.5, 7.9 Hz, 1H), 5.05 – 4.97 (m, 1H), 4.47 (d, *J* = 7.9 Hz, 1H), 4.16 – 4.08 (m, 2H), 3.97 – 3.84 (m, 2H), 3.74 (ddd, *J* = 10.2, 7.2, 3.3 Hz, 1H), 3.64 – 3.45 (m, 2H), 2.17 – 1.96 (m, 12H). ^{13}C NMR (101 MHz, Chloroform-d) δ 171.16 – 168.37 (m), 165.49, 130.63 (d, J = 14.1 Hz), 126.76 (d, J = 14.4 Hz), 101.46, 70.77 (d, J = 18.5 Hz), 69.02 (d, J = 15.3 Hz), 63.21, 61.39, 39.11 (d, J = 14.1 Hz), 21.07 – 19.82 (m). MS (ESI-positive): calculated for $C_{19}H_{27}NO_{11}H$ $[M + H]^+$, 446.1662; found 446.1660; calculated for $C_{21}H_{30}N_2O_{11}Na$ $[M + CH_3CN + Na]^+$, 509.1747; found 506.5309; calculated for $[M + CH_3CN + NH_4]^+$, 504.2193; found 506.5309; also found 588.8776.

Synthesis of 2,3,4-Tri-*O*-acetyl-*α*-*L*-fucosylethyl acrylamide (FucosEAm, **16, M5**). 1,2,3,4-tetra-*O*-acetyl-*α*-*L*-fucose (1.24 g, 3.73 mmol) was dissolved in DCM (20 mL), and *N*-(2-hydroxyethyl) acrylamide (0.753 g, 6.54 mmol) was added dropwise under a nitrogen atmosphere. The dropwise addition of $BF_3.Et_2O$ (2.94 mL, 23.79 mmol) was carried out at 0 °C (ice bath) for 10 min. Identical protocol was followed like **14** and **15**.[12] The crude product (yellow oil) was purified by silica gel column chromatography (hexane/EtOAc 1:3). The final product was pale yellow gum (yield = 0.66 g, 46%). TLC plates were stained with 0.1% napthoresorcinol solution and 1% H_2SO_4 solution in EtOH, followed by drying with a heat gun.

^{1}H NMR (400 MHz, $CDCl_3$) δ 6.29 (dq, *J* = 17.1, 1.6 Hz, 1H), 6.10 (ddt, *J* = 17.0, 10.3, 2.4 Hz, 2H), 5.69 – 5.60 (m, 1H), 5.35 – 5.22 (m, 1H), 5.17 – 5.09 (m, 1H), 5.06 – 4.99 (m, 1H), 4.47 (d, *J* = 7.9 Hz, 1H), 3.85 – 3.46 (m, 5H), 2.17 (d, *J* = 5.7 Hz, 3H), 2.06 – 1.98 (m, 9H). ^{13}C NMR (101 MHz, Chloroform-d) δ 171.11 – 169.29 (m), 165.57 (d, J = 16.3 Hz), 131.07 – 130.16 (m), 126.55, 101.32, 71.06 (d, J = 8.8 Hz), 69.33 – 68.74 (m), 68.22 – 67.27 (m), 64.71, 63.20, 39.72 – 38.47 (m), 21.49 – 19.75 (m), 15.98 (d, J = 23.2 Hz). MS (ESI-positive): calculated for $C_{17}H_{25}NO_9H$ $[M + H]^+$, 388.1601; found 388.1607.

2.4 Binding constant determination

Competitive binding assay measurements. Binding assays were performed according to the procedure described before.[13] BOB-ARS binding constant (K_{ars}) was measured first to determine the binding constants of BOB and other monosaccharides. Stock solutions of ARS ($9.0 \cdot 10^{-5}$ M), phosphate buffer (1.0 M pH 7.4), and BOB ($2.0 \cdot 10^{-2}$ M) were prepared in distilled water. Two solutions were prepared simultaneously. From the stock

solutions the first solution was prepared in distilled water containing BOB ($2.0 \cdot 10^{-3}$ M), ARS ($9.0 \cdot 10^{-6}$ M), and phosphate buffer (0.1 M). Another solution was also prepared containing only ARS ($9.0 \cdot 10^{-6}$ M), and phosphate buffer (0.1 M). The combined ARS and phosphate buffer solution was used as a fluorescence blank as the intensity was measured. 200 µL of the first solution was taken and fluorescence intensity was measured as first aliquot. The volume (200 µL) was kept the same as several aliquots' fluorescence intensities were measured with a constant ARS concentration and a decreasing BOB concentration. The measurements were conducted as a triplicate.

Fructose, sorbose, glucose, galactose, fucose were used. Stock solutions of ARS ($9.0 \cdot 10^{-5}$ M), phosphate buffer (1.0 M pH 7.4), and BOB ($2.0 \cdot 10^{-2}$ M) were prepared in distilled water. To determine their association constants with BOB three solutions were prepared from the stock solutions in distilled water. The first solution contained BOB ($2.0 \cdot 10^{-3}$ M), ARS ($9.0 \cdot 10^{-6}$ M), and phosphate buffer (0.1 M). The second solution (5 mL) contained BOB ($2.0 \cdot 10^{-3}$ M), ARS ($9.0 \cdot 10^{-6}$ M), and phosphate buffer (0.1 M), and monosaccharide (with varying diol concentrations). The concentrations of fructose and sorbose were 0.1 M, and 0.05 M respectively in the second solution (5 mL). For galactose, glucose, and fucose a concentration of 1 M was used in the second solution (5 mL). The third solution was prepared containing only ARS ($9.0 \cdot 10^{-6}$ M) and phosphate buffer (0.1 M) which was used as a fluorescence blank. 200 µL of the second solution was taken and fluorescence intensity was measured as first aliquot. The volume (200 µL) was kept unchanged as several aliquots' fluorescence intensities were measured with constant ARS and BOB concentrations with decreasing monosaccharide concentrations. The measurements were conducted as a triplicate and represented as a mean ± standard deviation.

Binding constant determination by isothermal titration calorimetry. Solutions of 10 mM BOB and 100 mM fructose and sorbose were prepared in a pH 7.4 0.1 M phosphate buffer. Sugar solutions were titrated into BOB solution to measure the molar ratio of complex formation at 298 K. Heat peaks were recorded over time on a MicroCal PEAQ-ITC calorimeter (Malvern Panalytical). Control measurements were conducted with buffer solutions to correlate the raw heat peaks' data with the measured experiments. The isotherms were analyzed with the built-in MicroCal PEAQ-ITC analysis software using the fitting routine for a single set of identical sites. The measurements were conducted as a triplicate and represented as a mean ± standard deviation.

2.5 Ink preparation:

Ink (ABOB) attachment to Rhodamine isothiocyanate (RhITC) and Fluorescein isothiocyanate (FITC). ABOB attachments to fluorescence dyes were performed according to the literature.[14] A solution of ABOB (20 mg, 0.134 mmol) was prepared in 2 mL dry DMSO. Rhodamine B isothiocyanate (RhITC, 67 mg, 0.125 mmol) were dissolved in 5 mL dry DMSO and stirred in a round bottom flask. The solution was degassed under N_2 for 5 min. ABOB solution was added to the RhITC solution under nitrogen atmosphere. The solution was stirred for 24 h at room temperature. DMSO was removed with lyophilization mixing with water (2 parts) for 48 h. ABOB-RhTTC conjugate was then used without any further purification to prepare a stock solution in DMSO (1mg mL^{-1}). From the stock solution, other solutions in buffer (2 µg mL^{-1}) were prepared which was used to ink the grafted PDMS stamps. ABOB-FITC conjugate was prepared similarly. In this case DMSO was not removed via lyophilization rather the solution was used directly without further purification to prepare dye solution (2 µg mL^{-1}) to ink the PDMS stamps. The ABOB-dye conjugates were used without further purification.

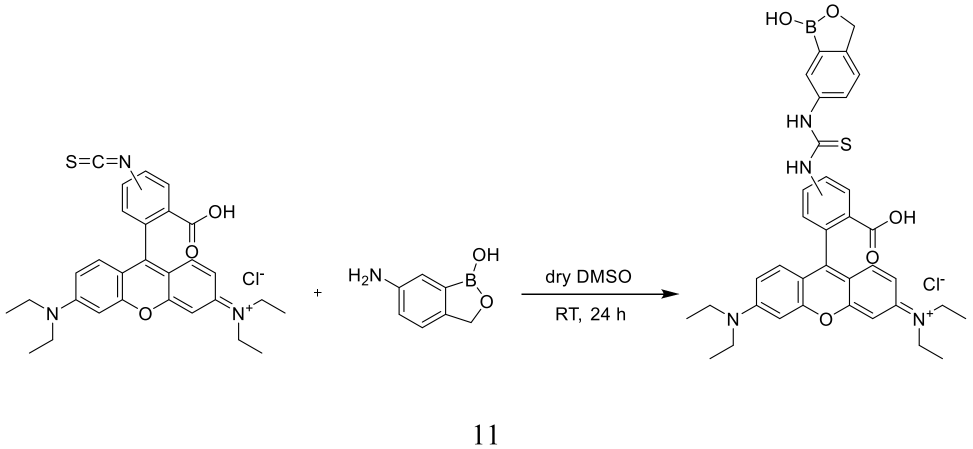

Figure S4. ABOB conjugation with RhITC.

2.6 RAFT polymerization of sugar monomers:

Kinetic study of the sugar monomer polymerization reaction in solution. 4-Cyano-4-[[(dodecylthio)carbonothioyl]thio]pentanoic acid (0.5 mg, 0.0012 mmol) and AIBN (0.203 mg, 0.0012 mmol) were taken from their respective stock solutions in DMF (maintaining a 1:1 CTA/initiator ratio) and added to the corresponding monomer solutions. A final solution (1.5 mL) with a concentration of 0.33 mol L^{-1} was prepared using DIFMAm (**M1**, 162 mg, 0.495 mmol), DISMAm (**M2**, 162 mg, 0.495 mmol), GlucosEAm (**M3**, 220 mg, 0.495 mmol), GalactosEAm (**M4**, 220 mg, 0.495 mmol), and FucosEAm (**M5**, 192 mg, 0.495 mmol) in DMF. The solutions were purged with nitrogen for 30 min before initiating the polymerization at 70 °C for 48 hours. Aliquots were collected at 0, 1, 2, 4, 20, 24, and 48 hours for analysis by ^{1}H NMR and SEC (using THF as the eluent and PS standard for calibration).

2.7 Preparation of sugar-substrates:

Grafting sugar polymer from Si wafer surfaces. Si wafers (1 cm^2) were washed with EtOH and dried with air stream. Wafers were plasma treated (100 W, 60s, 100 air). APTES functionalization and CTA attachment to the surface were done in the same way as described before for PDMS stamp (shuttle CTA approach meaning excess CTA in the solution). 4-Cyano-4-[[(dodecylthio)carbonothioyl]thio]pentanoic acid (0.2 mg, 0.0005 mmol) and AIBN (0.411 mg, 0.0025 mmol) were taken from their respective stock solutions in DMF (CTA/initiator 1:5) and added to respective monomer solutions. A final 0.5 mL solution prepared with DIFMAm (**9**, **M1** 82 mg, 0.25 mmol), DISMAm (**10**, **M2** 82 mg, 0.25 mmol), GlucosEAm (**14**, **M3** 89 mg, 0.2 mmol) GalactosEAm (**15**, **M4**, 111 mg, 0.25 mmol), FucosEAm (**16**, **M5**, 97 mg, 0.25 mmol). CTA attached wafers were immersed in the solutions in vials. The solutions were purged with N_2 for 30 min and the polymerization reaction was carried out at 70 °C for 24 h. The wafers were then rinsed with DMF and Milli-Q water multiple times. The resulting grafted polymers are P**1**, P**2**, P**3**, P**4**, and P**5**. P**1** – **2** and P**3** – **5** were formed in solution as well which were first characterized via ^{1}H NMR indicating broadening of the backbone peaks, followed by SEC (THF as eluent, PS standard, **Table S2**).

Hydroxy group (-OH) **deprotection of the grafted-polymers**. For the acetal deprotection of PDIFMAm and PDISMAm (P**1**, P**2**), the wafers were immersed in TFA/H_2O (9:1) solution for 5 h. The procedure was taken from literature.[15] After deprotection, the wafers were washed with water thoroughly and dried at 60 °C under vacuum for 20 min. PGlucosEAm, PGalactosEAm, and PFucosEAm (P**3**, P**4**, and P**5**) were deprotected by immersing them in a sodium methoxide (NaOMe) solution for 24 h. The solution was prepared in dry methanol in such a way that the pH value would be around 9 as measured with pH indicator strips. The wafers were then washed with methanol, rinsed with ethanol, and dried under the air stream.

Determination of water contact angle (CA). After deprotection of the polymer grafted wafers water contact angles were measured. 2 µL water drops were injected with the syringe of the contact angle measurement device (as mentioned in the instrument section) without any disruption. Contact angle was measured as an average of drops at different spots of the wafers. Errors were measured as standard deviation.

2.8 Preparation of microgel particles and cells

Microgel preparation. The microgel particles were prepared in the microfluidic channel. Pure 1,6-hexanediol diacrylate (HDDA) was used as disperse phase, where 1wt% SDS solution was used in the aqueous phase. Poly(styrene)-*b*-poly(acrylic acid) (PS-*b*-PAA) were added to the HDDA in the microfluidic channel and the

particles were generated with PS-*b*-PAA chains on the microgel surface. The droplets were collected and then polymerized under UV light (375 nm) for 3 min. In the presence of an ice bath 3-amino phenyl boronic acid (APBA, 1.043 mg, 0.007 mmol) was added in water. After 15 min, 1-hydroxybenzotriazole hydrate (HOBT, 0.152 mg, 0.0011 mmol) and 4-(dimethylamino)pyridin (DMAP, 0.137 mg, 0.0011 mmol) were added to it. After 5 min, 1-ethyl-3-(3-dimethylaminopropyl)carbodiimide (EDC, 0.871 mg, 0.0056 mmol) was introduced and stirred for 30 min. The particles were then added to the solution and stirred vigorously for at least 24 h. Subsequently, the particles were washed with water and dried at room temperature.

Around 200 particles were transferred in 1 mL PBS solution, and 20 mg FITC-Dextran 40k was added. The mixture was stirred for 12 h. After that the particles were washed thoroughly with water to remove any unattached Mil-Dextran before observation under a confocal microscope.

To functionalize the particles with P2 for µCP, P2 was synthesized in DMF as described earlier and treated with TFA/water (9/1) to deprotect the hydroxy groups. The polymer solution was then dried to remove any DMF and TFA. 10 mg of the resulting P2 was dissolved in 0.5 mL Milli-Q water. Dried microgel particles were dispersed to the P2 solution maintaining a pH 8.5 for 30 min in Tris buffer. The particles were then washed several times with the buffer solution to remove any excess sugar and TFA.

Cell preparation. Primary gastric epithelial cells (Innoprot, Derio, Spain) were cultivated in epithelial cell medium under static conditions in a standard humidified incubator at 37°C with 5% CO_2 supplemented with penicillin/streptomycin, epithelial cell growth supplements and fetal bovine serum (Innoprot, Derio, Spain). For cell experiments gastric cells were seeded in passage 6 on glass slides coated with poly-L-lysine (2 µg cm^{-2}, ScienCell, Carlsbad, USA) for 24h. The cell culture medium was aspirated, cells were washed with PBS ($PBS^{+/+}$) for 3 min, followed by 10 min incubation with ice cold 4% formaldehyde for cell fixation. Subsequently, cells were washed 3 times with PBS without magnesium and calcium ($PBS^{-/-}$). For permeabilization, cells were incubated with 0.25% Triton X-100 solution (VWR, Darmstadt, Germany) for 10 min, followed by washing with $PBS^{-/-}$). Thereafter, cell nuclei were stained using 4',6-diamidino-2-phenylindole (DAPI). Additionally, L929 cells were cultivated in MEM (Gibco, Schwerte, Deutschland) under static conditions in a standard humidified incubator at 37°C with 5% CO_2 supplemented with 10% fetal bovine serum (PAN, Aidenbach, Germany). Then, the cells were seeded confluently in passage 10 on glass sides for 24h. For cell printing procedure, the cell culture medium was aspirated, and cells were washed with PBS ($PBS^{+/+}$) for 3 min, followed by 10 min incubation with ice cold 4% formaldehyde for cell fixation. Subsequently, cells were washed 3 times with PBS without magnesium and calcium ($PBS^{-/-}$). Thereafter cell nuclei were stained using DAPI.

µCP on sugar-modified surfaces. PDMS stamps were suspended to HCl solution (1 M) to remove the boronic acid protection groups from dopamine. To print on sugar-modified Si wafers, the stamp was washed with Milli-Q water and immersed in 10 µg mL^{-1} ABOB-RhITC (in pH 7.4, 0.1 M phosphate buffer) solution for 30 min. The stamp was washed with the same buffer afterwards. Printing was carried out in a custom-made chamber in presence of the buffer solution (*ca.* pH 8, 0.1 M phosphate buffer). The stamp was put on top of the wafer and the lid was closed. Using Zumolab microcontact printer 1 N force was applied for 20 min. Printing with a bare stamp was performed in a similar manner. To remove patterns from fructose surface, the printed substrate was washed with pH 4 phosphate buffer (0.1 M) solution in a glass vial by shaking for 2 h.

µCP on sugar-modified surfaces for different durations. Above mentioned printing protocol was employed to print on sorbose, fructose, glucose, galactose, and fucose modified surfaces. Each sugar modified surface was printed with the inked stamp at 1 N force for 2, 5, 10, 20, and 30 min in a small chamber in the presence of an aqueous buffer solution (pH *ca.* 8).

Multiple printing with a single stamp. Printing was carried out with a single stamp 4 times on the sorbose modified surfaces at 1 N for 20 min. The stamp was washed with Milli-Q water thoroughly each time after printing and re-inked to print on a fresh surface.

Printing on microspheres. First fructose functionalized microgel particles were attached onto a glass surface. For this purpose, *ca.* 1 × 1 cm^2 glass substrates were plasma treated (100 W, 60 s, 100 air). Glass substrates

were then APTES-functionalized via chemical vapor deposition (CVD) at 70 °C. The plasma treated substrates were kept in a PFA chamber with APTES and aq. solution of NH_4OH for 2 h. A solution of succinic anhydride (SAA, 5 mg, 50 µmol), TEA (5%, 50 µL) were dissolved in 1 mL THF. The glass substrates were immersed for 3 h in the solution and washed with THF and EtOH afterwards. The substrates were dried under soft air stream and immersed in a solution of ABOB (5 mg, 0.033 mmol), DCC (8 mg, 0.038 mmol), TEA (5 mg, 0.05 mmol), DMAP (0.2 mg, 0.0016 mmol) in 1 mL DMF for 24 h. The substrates were washed with EtOH and dried. The particles in Tris buffer (pH 8.5) were suspended onto the glass substrate and kept overnight. Subsequently, a poly(NAM-*co*-DMA-p) stamp was first treated with pH 1 HCl solution for 20 min and washed with Milli-Q water to remove the borax protection of the catechol. The attached microgel particles on glass were then put in a small printing chamber and printed with an inked stamp (ABOB-RhITC, 10 µg in 1 mL Tris buffer, pH 8.5, 30 min). A printing force of 1.3 N was used for 20 min.

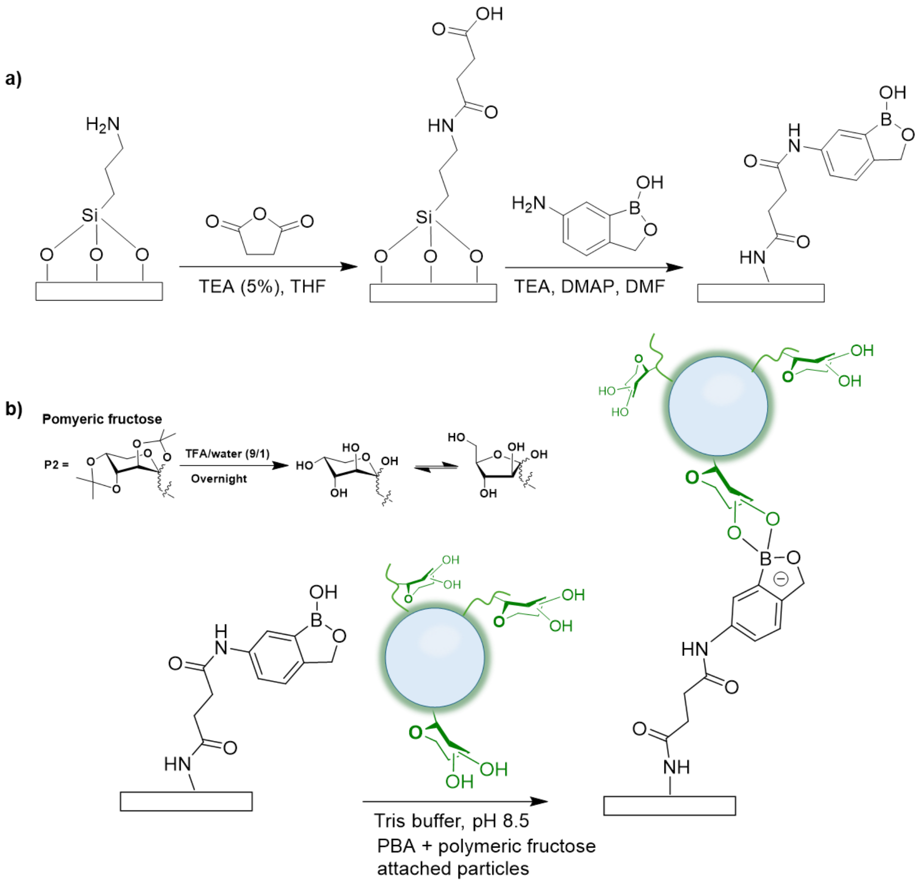

Figure S5. a) ABOB attachment to glass surface for microgel attachment *via* ABOB-diol conjugation, b) sugar attached (micro)particles fixation to ABOB-functionalized glass surface.

Printing on cell membrane. Printing on human gastric cells was done using inked PDMS stamps with ABOB-FITC solution (pH 7.4 phosphate buffer, 0.1 M). The inking and printing procedure was similar to that for sugar-modified wafers. (Positive control experiment was performed with a grafted stamp. The stamp's catechol contained polymer was first deprotected in pH 1 HCl solution and washed with Milli-Q water. The stamp is then inked with FITC-ABOB conjugated dye (10 µg mL^{-1}) for 30 min. Cells fixed on the glass coverslip were kept in a small printing chamber (*ca.* 2 cm^2) and immersed with the pH 7.4 buffer solution. The stamp was put on top of the cell surface and printing was carried out for 20 min with 1 N force from top. The negative control experiment (**Figure 7**) was done following a similar method except for Triton-X treated cell membranes. Cells were also treated with acetic anhydride in acetonitrile for 3 hours, washed with same buffer, and printed as described. Another negative control experiment was performed with a bare stamp (non-grafted stamp). The bare stamp was inked with ABOB-FITC and printed on the cell membranes. Additionally, Printed cells (positive control) were washed with pH 4 phosphate buffer (0.1 M) overnight to remove patterns. In another experiment, cultured L929 cell membrane was patterned in a similar way. Positive control was carried out with a grafted stamp, whereas

negative control was performed using a non-grafted bare stamp. The stamps were inked with ABOB-FITC for 30 min. After washing with a buffer solution (pH 7.4), printing was carried out with 1N force for 20 min.

3. Figures and Tables

3.1 Boronic acid and sugar interaction:

Benzoxaborole (BOB) complex formation with 4-methylcatechol and fructose:

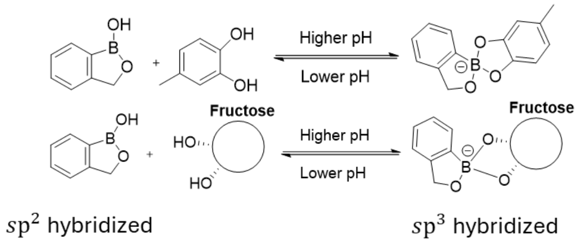

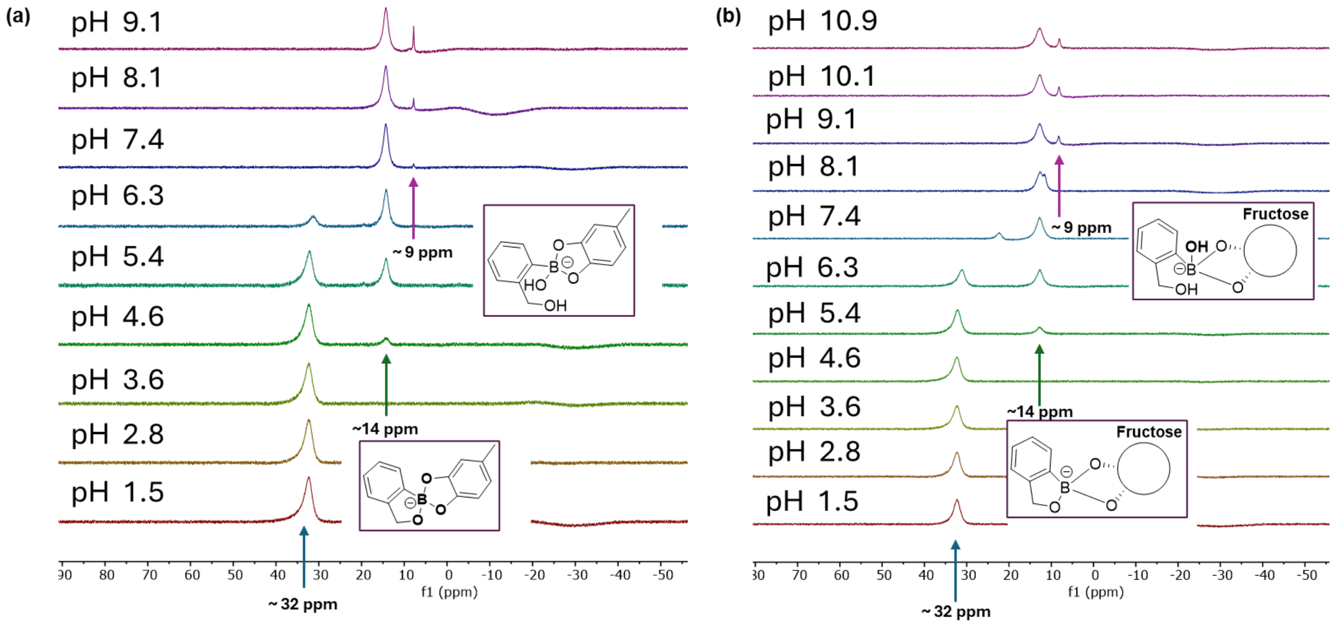

Figure S6. Benzoxaborole ester formation at high pH in aqueous medium by transforming from sp^2 hybridized state to sp^3 hybridized state. ^{11}B NMR Spectra of a) BOB- 4-methylcatechol and b) BOB- fructose mixtures at different pH values to determine the complex formation. Complete complexation or boronate ester formation at pH > 7 was seen in both cases as indicated by the peak at ~ ppm 14. Free boronic acids are indicated by the signals at ~ppm 32, whereas peaks at ~ppm 9 represent the open five-membered ring of BOB.

^{1}H NMR of complex formation between BOB and fructose or sorbose

The experiments were performed in a 0.1 M buffer at pH 7.45, with both BOB and sugars at a concentration of 10 mM for 1:1 BOB-to-sugar ratio. In all ^{1}H NMR spectra of BOB mixtures with sugar, two forms

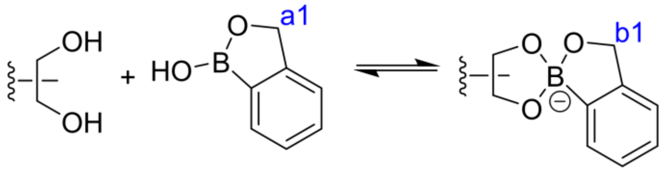

of BOB are observed. The peaks corresponding to the CH_2 group of the five-membered ring are indicated as a1 and b1, for benzoxaborole and its ester, respectively.

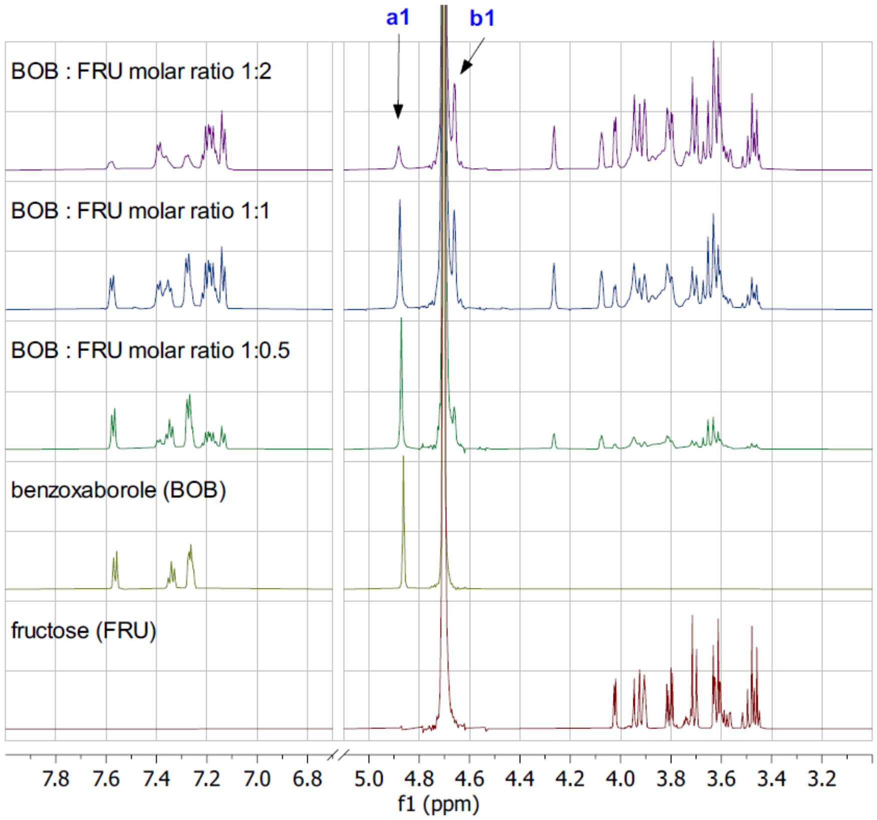

Figure S7. Benzoxaborole ester formation in aqueous medium with *D*-fructose at different ratios of BOB-to-sugar.

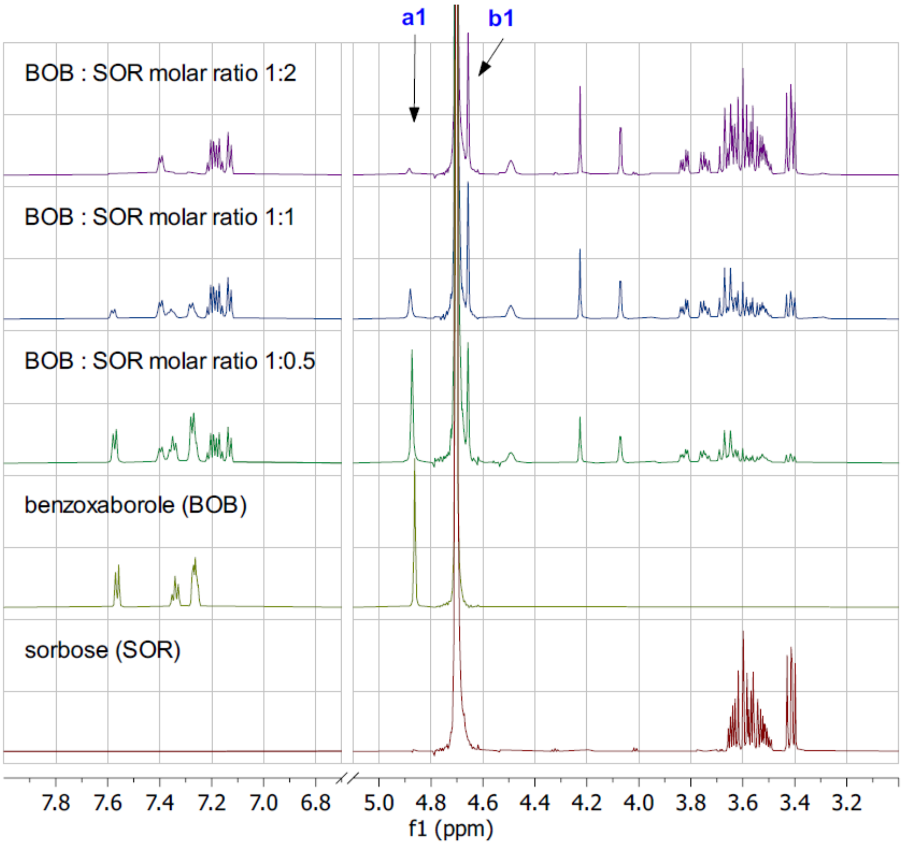

Figure S8. Benzoxaborole ester formation in aqueous medium with *L*-sorbose at different ratios of BOB-to-sugar.

For the purpose of discussing NMR data, the complex formation reaction

$$\text{BOB} + \text{carbohydrate} \rightleftharpoons \text{carbohydrate} \cdot \text{BOB},$$

is abbreviated as,

$$\text{A} + \text{S} \rightleftharpoons \text{B}$$

Where A and B are the two forms of BOB, free benzoxaborole and benzoxaborole ester, respectively, and S represents either fructose or sorbose. The association constant is defined as

$$K_a = \frac{[B]}{[A][S]} = \frac{k_1}{k_{-1}}$$

Where $[\cdot]$ denotes molar concentrations, k_1 and k_{-1} are forward and backward reaction rate constants. The concentrations $[A]$ and $[B]$ can be derived from integral intensities of ^{1}H peaks a1 and b1, respectively.

The ratio,

$$r = \frac{[B]}{[A]}$$

is simply the ratio of the b1 and a1 peak integrals. Using the known total concentrations of BOB and sugar, denoted as $[A]_0$ and $[S]_0$, respectively, we can calculate the association constant as follows.

$$[A]_0 = [A] + [B]$$

$$[S]_0 = [S] + [B]$$

$$[A] = [A]_0 - r[A] \;\rightarrow\; [A] = [A]_0 \frac{1}{1+r}$$

$$[S] = [S]_0 - r[A] \;\rightarrow\; [S] = [S]_0 - \frac{r}{1+r}[A]_0$$

$$K_a = \frac{r}{[S]} = \frac{r}{[S]_0 - \frac{r}{1+r}[A]_0}$$

The a1, b1 peak integrals were determined using spectral deconvolution (line fitting) tool of MestreNova software. The fits are presented in **Figure S9**. Three peaks were fitted to account for the strong residual water signal. The association constants were determined independently for spectra of 1:0.5, 1:1, and 1:2 molar ratios of BOB and sugar. In case of fructose, K_a was within the range 130 - 160 M^{-1} (mean value 145 M^{-1}), for sorbose it was 470 - 640 M^{-1} (mean value 580 M^{-1}). These values are in good agreement to the values determined by the fluorescence binding affinity assay.

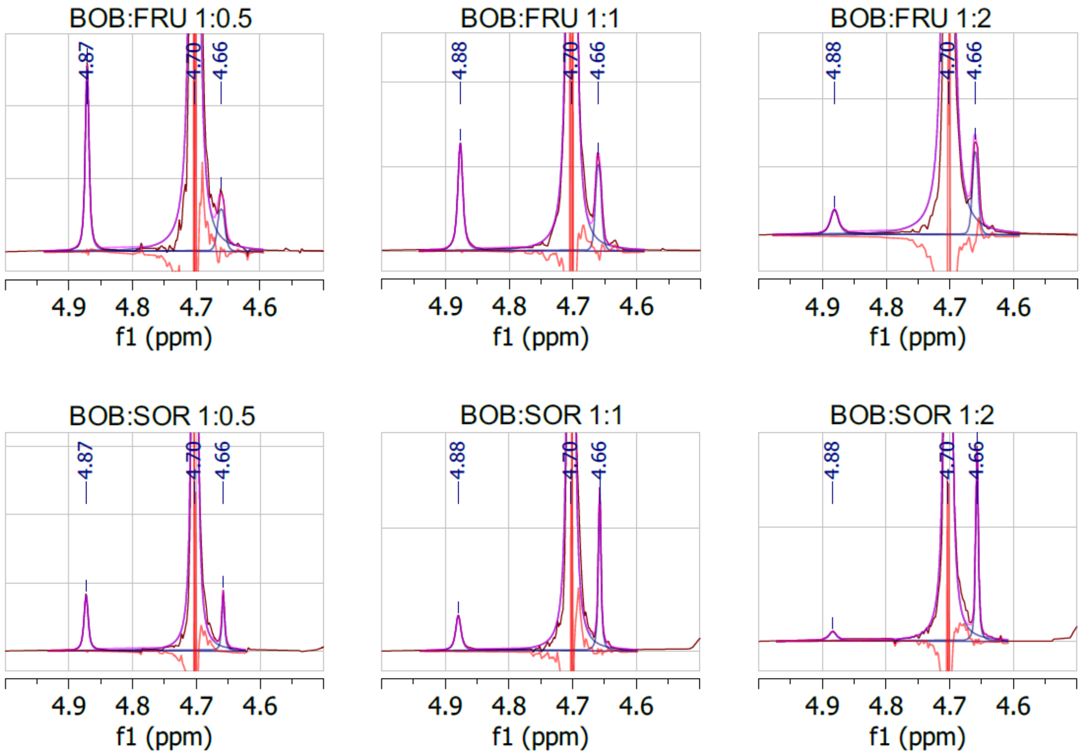

Figure S9. Spectral deconvolution of ^{1}H NMR to obtain integral intensities for peaks a1 and b1. During the fitting process, special attention was paid to closely represent the shoulder of the water resopHce towards the b1 peak at 4.66 ppm.

The static picture of the complexation process is complemented with estimation of exchange dynamics. For samples with BOB-to-sugar molar ratio 1:1, we measured selective EXSY where the signal at 4.88 ppm (a1) was selectively excited. The intensity of the peak at 4.66 ppm (b1) was observed as a function of mixing time, going from 3 ms up to 1 s in 22 steps. Peak integrals were normalized according to the intensity of the excited peak, assuming zero mixing time. Data were evaluated using initial buildup analysis, fitting the first 15 points using a quadratic function and taking its derivative at time zero as the rate constant, denoted as k_{FB}. The results are presented in **Figure S10**. The exchange rate for BOB:fructose was determined to be 1.21 s^{-1}, whereas for BOB:sorbose it was 0.24 s^{-1}. Note, however, that these values do not correspond to the forward reaction rate k_1 directly. The appropriate relation is

$$k_{FB} = [A]k_1$$

and thus the k_{FB} values depend on sample concentrations. On the other hand, the backward reaction rate k_{-1} could be estimated directly, when the peak b1 is excited and the exchange of magnetization towards the peak a1 is monitored (decomposition is a single molecule process). Unfortunately, such experiment was impossible due to the presence of the strong water signal in close vicinity to b1. Nevertheless, k_{-1} rate can be derived from k_{FB} and the association constant.

$$k_1 = \frac{k_{FB}}{[A]} = k_{FB}\frac{1+r}{[A]_0}$$

$$k_{-1} = \frac{k_1}{K_a}$$

For the complex with fructose, k_{-1} is about 1.5 s^{-1}, and in case of sorbose it is about 0.13 s^{-1}. The inverse value of k_{-1} can be interpreted as a complex lifetime, yielding 0.7s and 7.8s for BOB:FRU and BOB:SOR, respectively. These findings confirm that the fructose-BOB interaction is weaker than the

sorbose-BOB interaction, as weaker binding typically allows for more rapid association and dissociation dynamics, leading to faster exchange rates in NMR experiments.

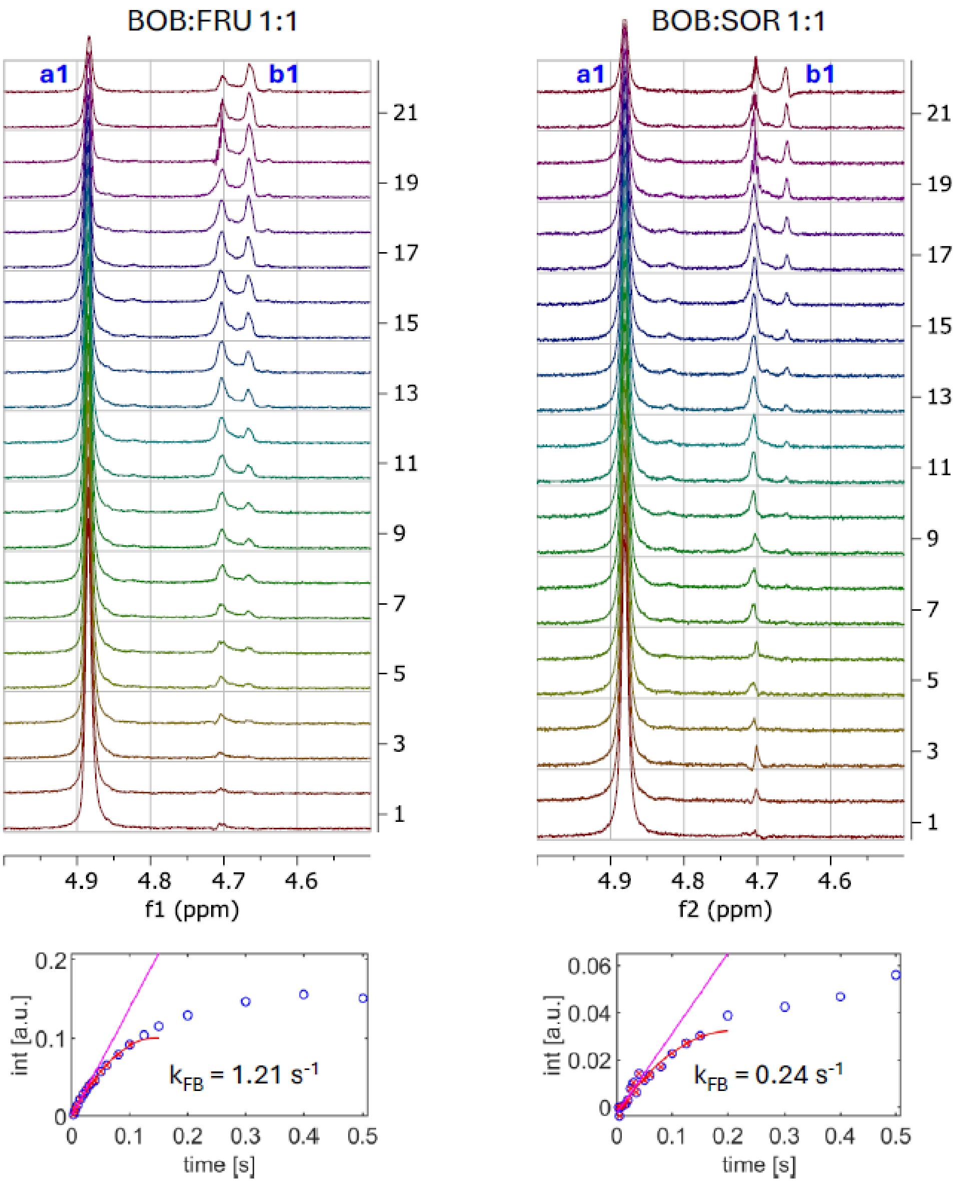

Figure S10. Benzoxaborole ester formation in aqueous medium with *D*-fructose (left) and *L*-sorbose (right) at 1:1 ratio of BOB-to-sugar. The bottom panels represent results of the initial buildup analysis to determine the exchange rate.

3.2 Monomers for grafting from the stamp:

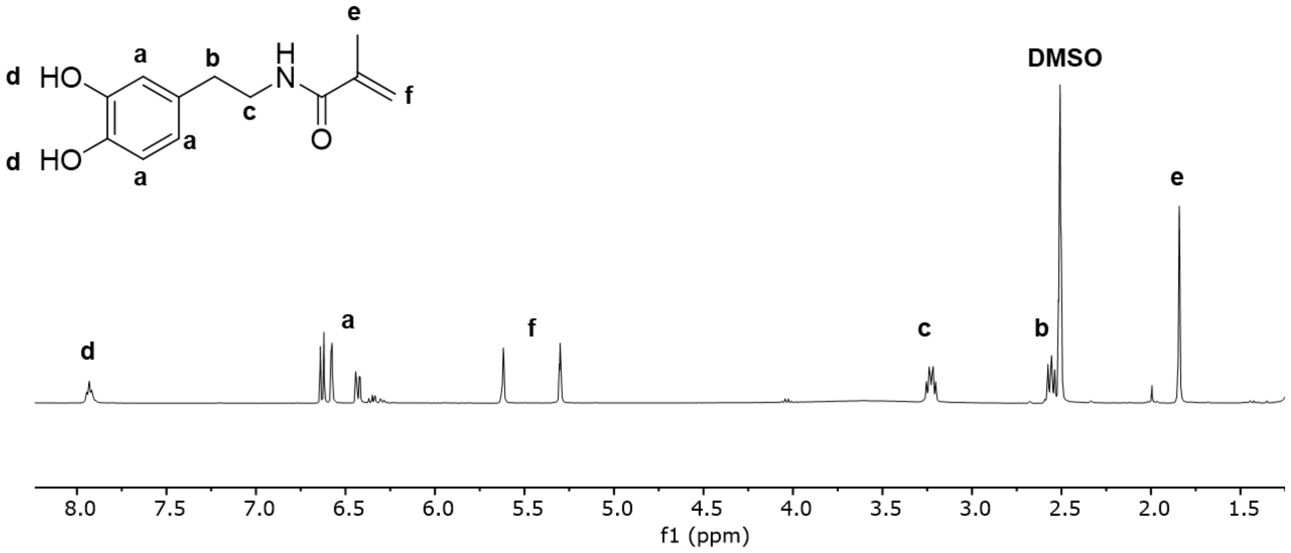

Figure S11. ^{1}H NMR (in DMSO, d_6) of dopamine methacrylamide (DMA).

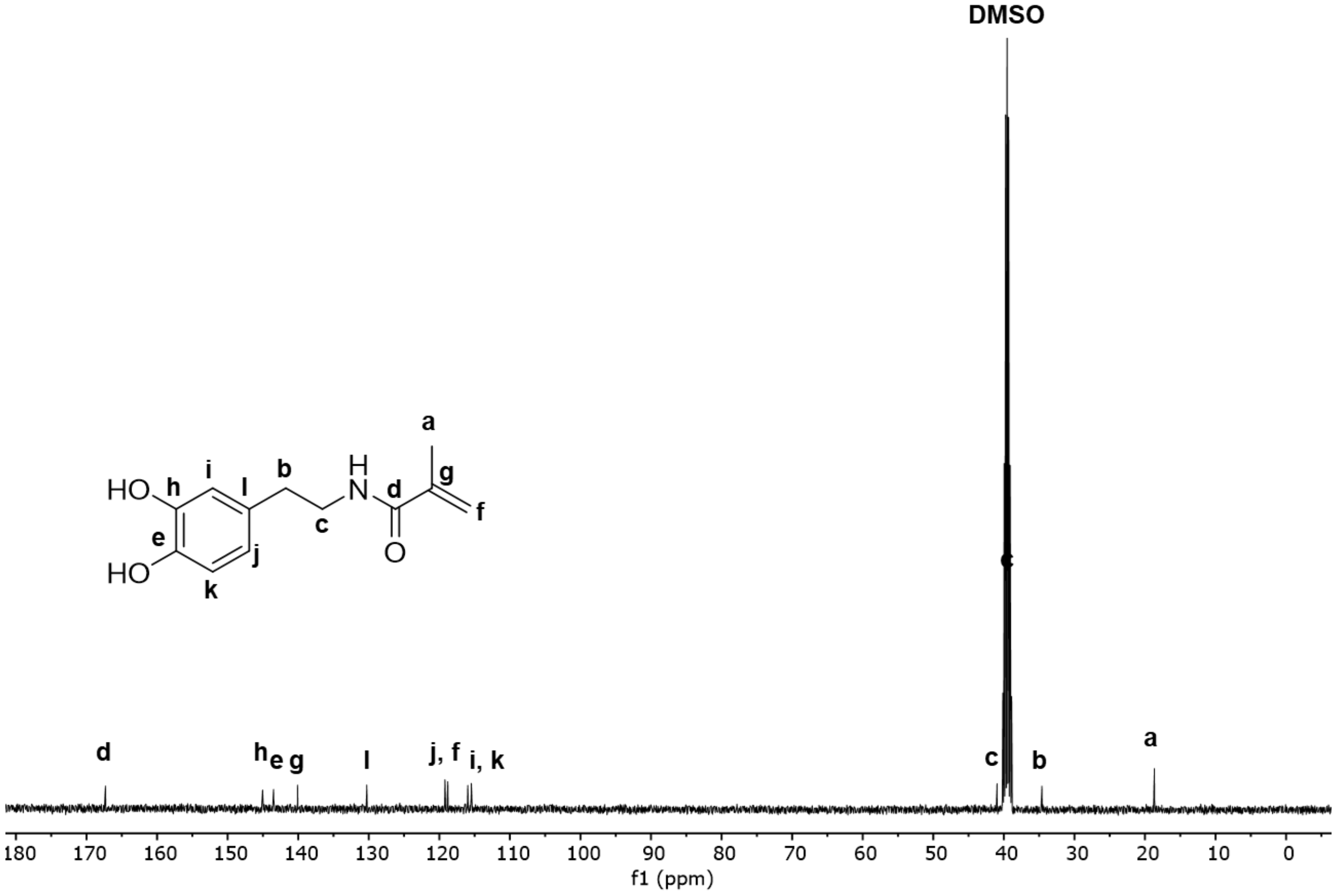

Figure S12. ^{13}C NMR (in DMSO, d_6) of dopamine methacrylamide (DMA).

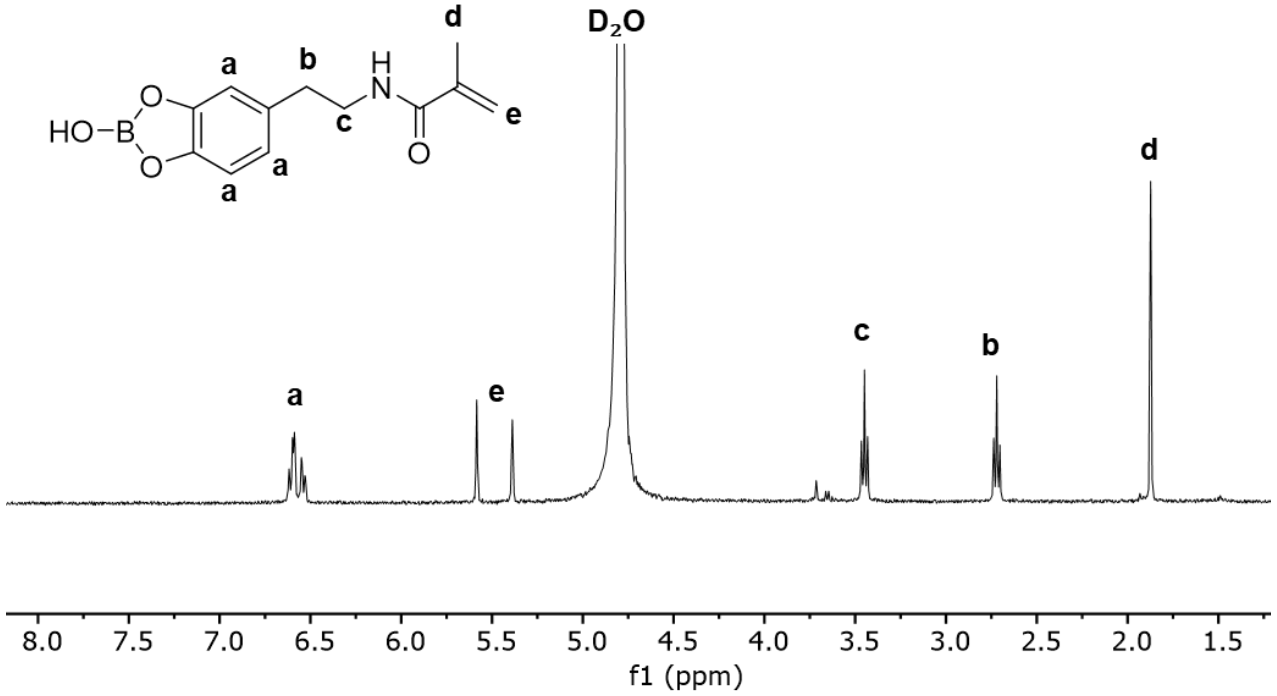

Figure S13. ^{1}H NMR (in D_2O) of borax-protected DMA-p.

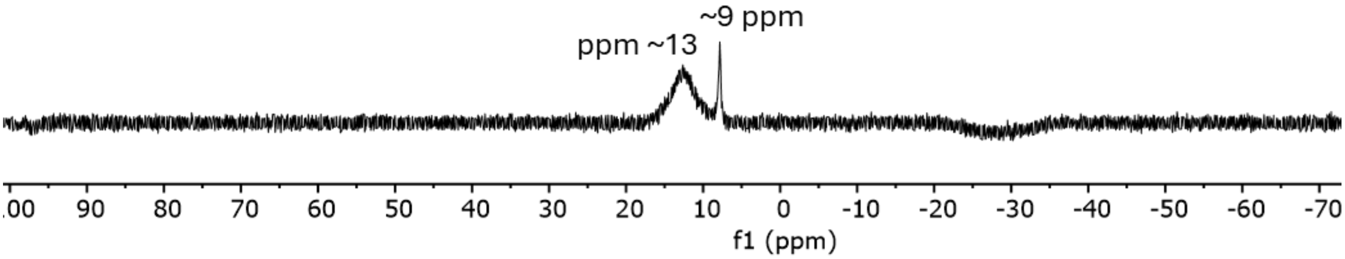

Figure S14. ^{11}B NMR (D_2O) of borax-protected DMA-p.

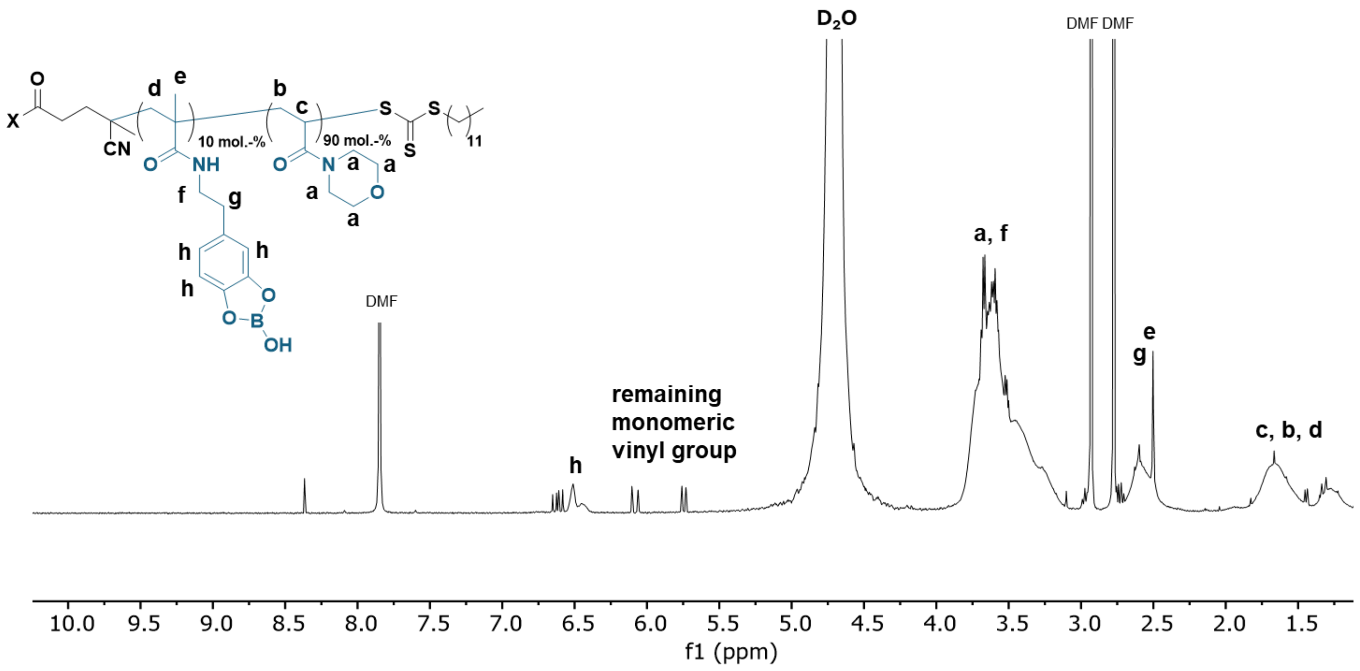

Figure S15. ^{1}H NMR (in D_2O) of poly(90%NAM-*co*-10%DMA-p) formed in grafting solution. DMF was used as a part of the solvent during polymerization.

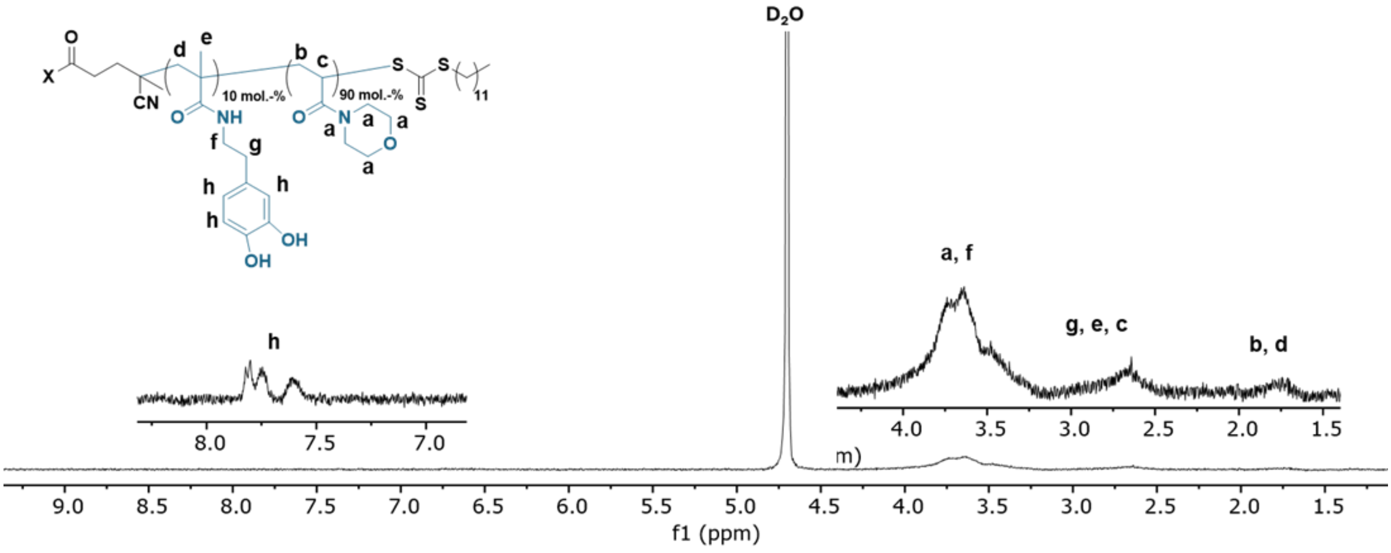

Figure S16. ^{1}H NMR (in D_2O) of poly(90%NAM-*co*-10%DMA) after purification via dialysis (3.5 kDa MWCO). Zoomed in areas show the appropriate signals.

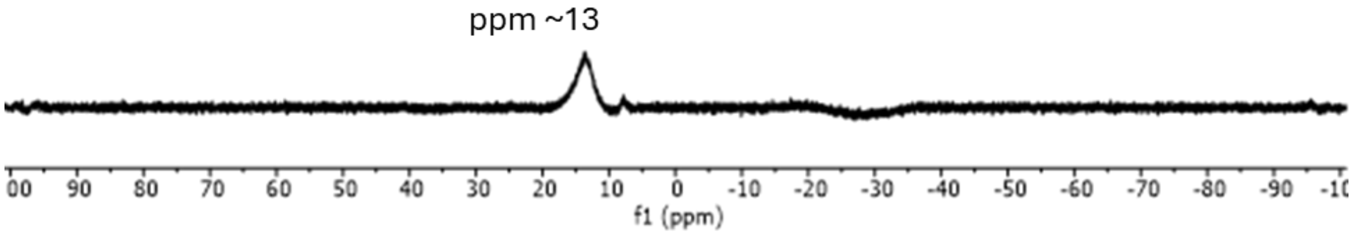

Figure S17. ^{11}B NMR (in D_2O) of borax-protected polymer resembling the protected monomer.

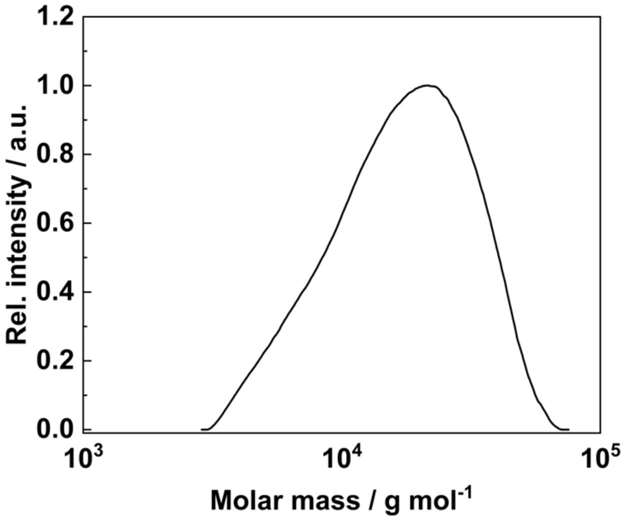

Figure S18. Representative SEC curve of poly(NAM-*co*-DMA) polymers. The solution-formed polymers *via* shuttle CTA RAFT polymerization provide an idea about the grafted polymer onto the PDMS stamp surface. SEC was measured in aqueous eluent (0.1M NaCl and 0.3% formic acid, PVP standard. M_n = 13.9 Kg mol^{-1}, Đ = 1.44).

3.3 Sugar monomer syntheses and characterization

3.3.1 Synthesis of sugar monomers

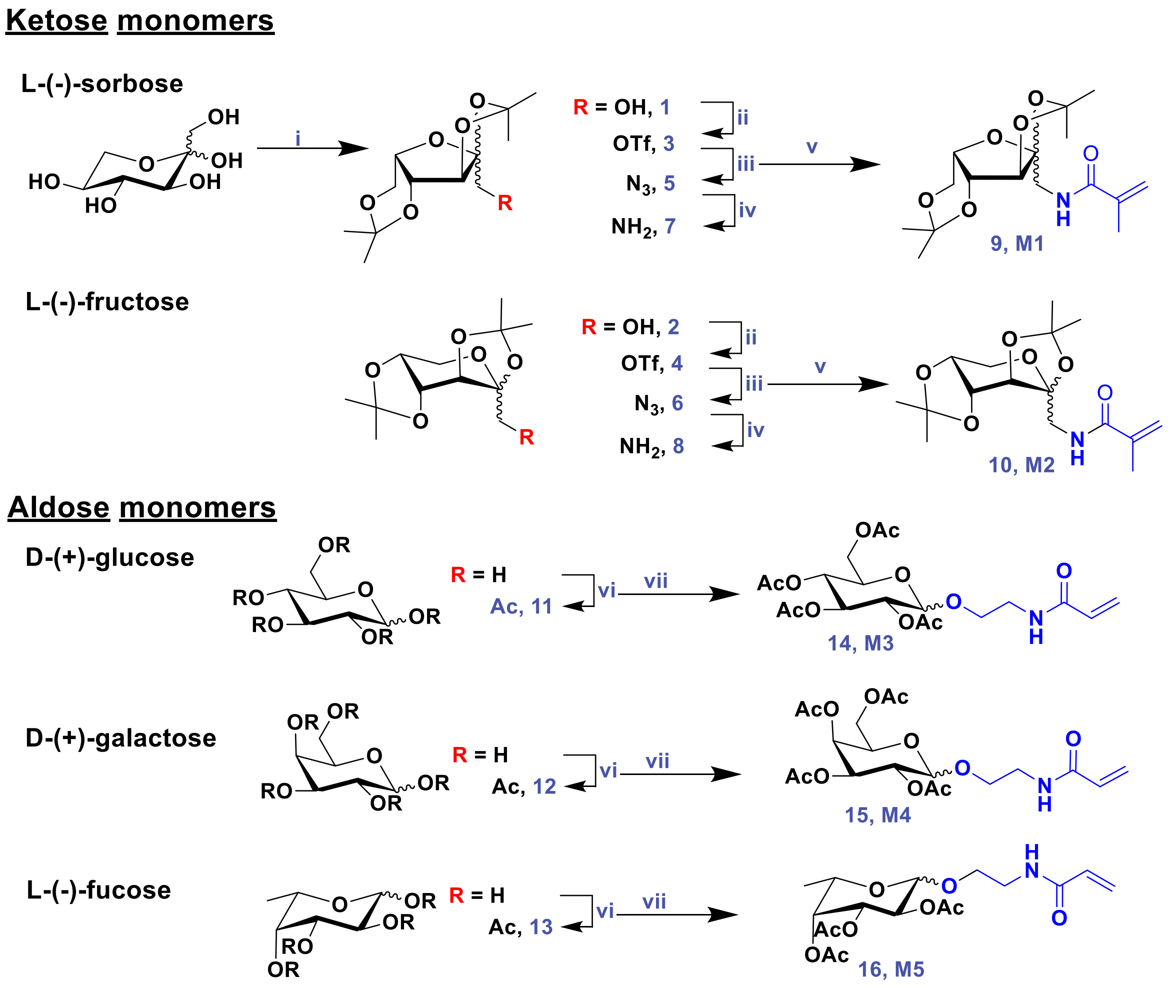

Figure S19. Preparation of saccharide-monomers along with their deprotection strategies. Ketose monomers (sorbose and fructose) were synthesized in a 4-step process to convert them into corresponding methacrylamides (**M1** and **M2**). Reaction condition and procedure is described in the experimental section. Aldose monomers (glucose, galactose, and fucose) were synthesized from their pentaacetylated forms to their acrylamides (M3, M4 and **M5)** respectively. i) I_2, acetone (RT, 18 h), yield: **1** = 74%), ii) Tf_2O, pyridine (-10 °C, 4 h), (yield: **3** = 87%, **4** = 89%) iii) NaN_3 (DMF, 55 °C, overnight), (yield: **5** = 87%, **6** = 88%), iv) Triphenyl phosphine (a. DMF, RT, overnight; b. DMF, H_2O, 110 °C, 2 h), (yield: **7** = exact % cannot be determined as Ph_3O cannot be separated, **8** = 77%), v) Methacrylic anhydride (RT, 24 h), (yield: **9, M1** = exact % cannot be determined as **7** was not pure, **10, M2** = 73.5%), vi) acetic anhydride, pyridine (RT, 3 d) vii) BF_3. Et_2O, (*N*-hydroxyethyl) acrylamide (DCM, RT, 3 d), (yield: **14, M3** = 19%, **15, M4** = 50%, **16, M5** = 46%).

3.3.2 Ketose monomers (*L*-sorbose derivatives):

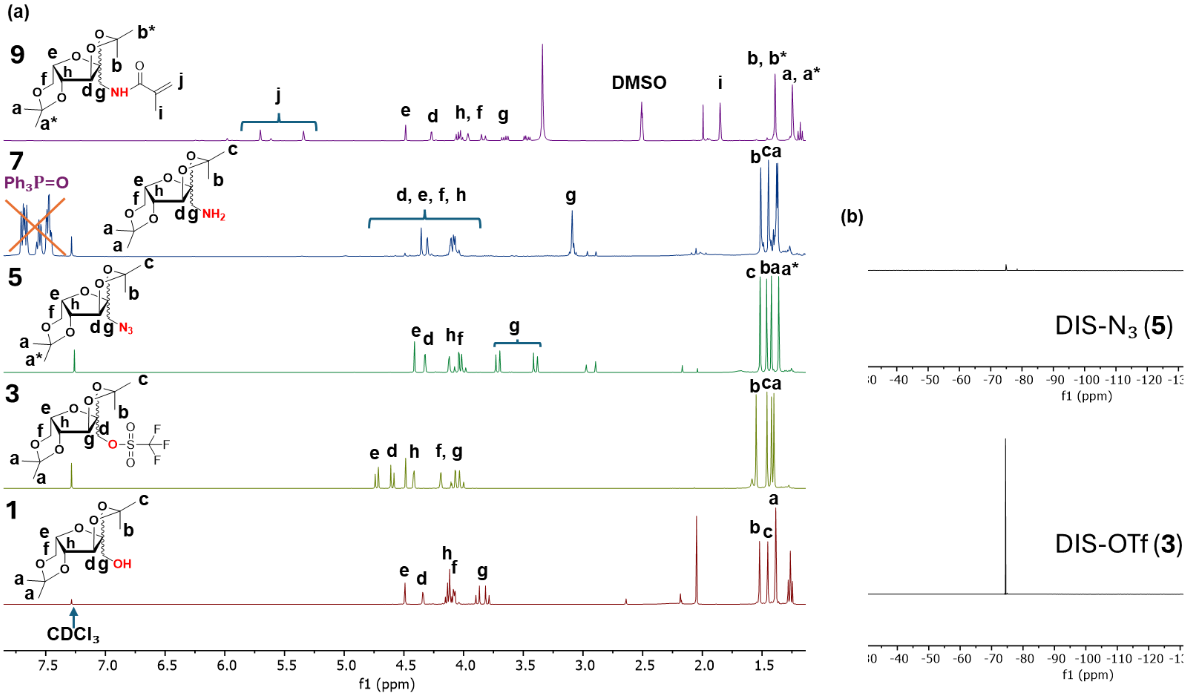

Figure S20. ^{1}H NMR of intermediate sorbose compounds and the final monomer **9** (**M1**). $CDCl_3$ was used as NMR solvent, except **9** that was dissolved in DMSO (d_6) for NMR analysis. At 3.3 ppm, the water peak is seen (for **9**). **b)** ^{19}F NMR (performed in $CDCl_3$) of **3** and **5** to compare the presence and absence of fluorine peak between the compounds which indicates the successful conversion of azide derivative from the former triflate derivative of 2,3:4,6-Di-*O*-isopropylidene-*L*-sorbose.

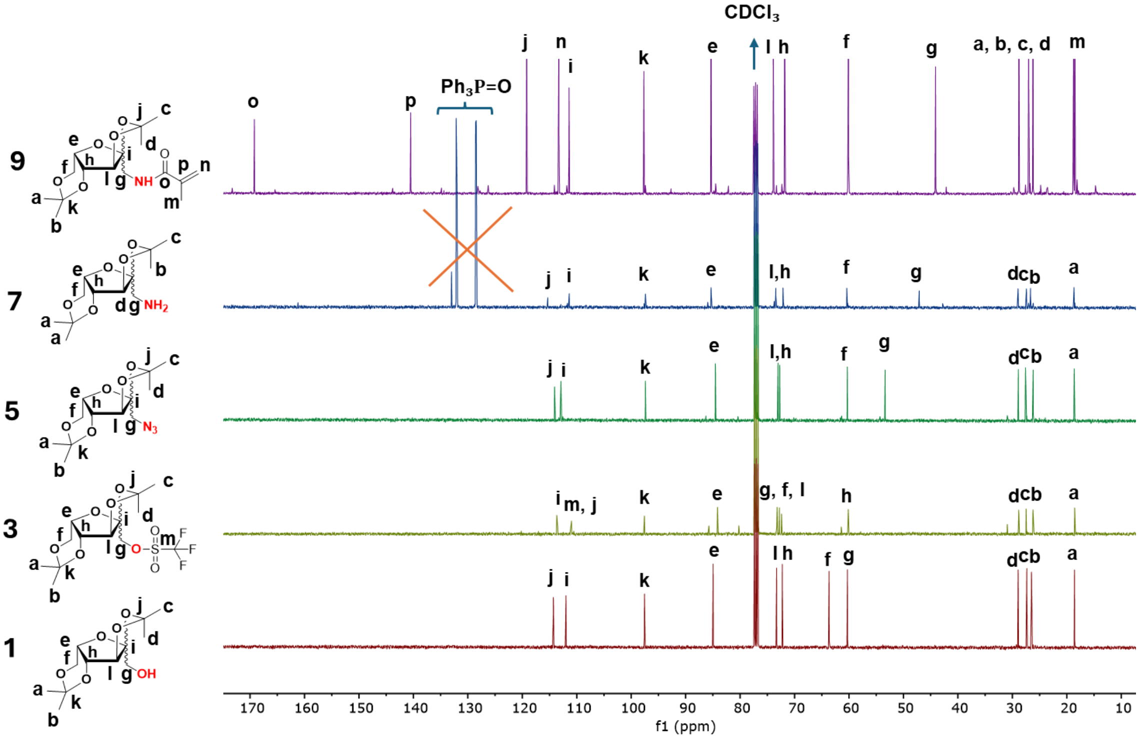

Figure S21. ^{13}C NMR of intermediate sorbose compounds and the final monomer **9** (**M1**. $CDCl_3$ was used as NMR solvent.

3.3.3 Ketose monomers (*D*-fructose derivatives):

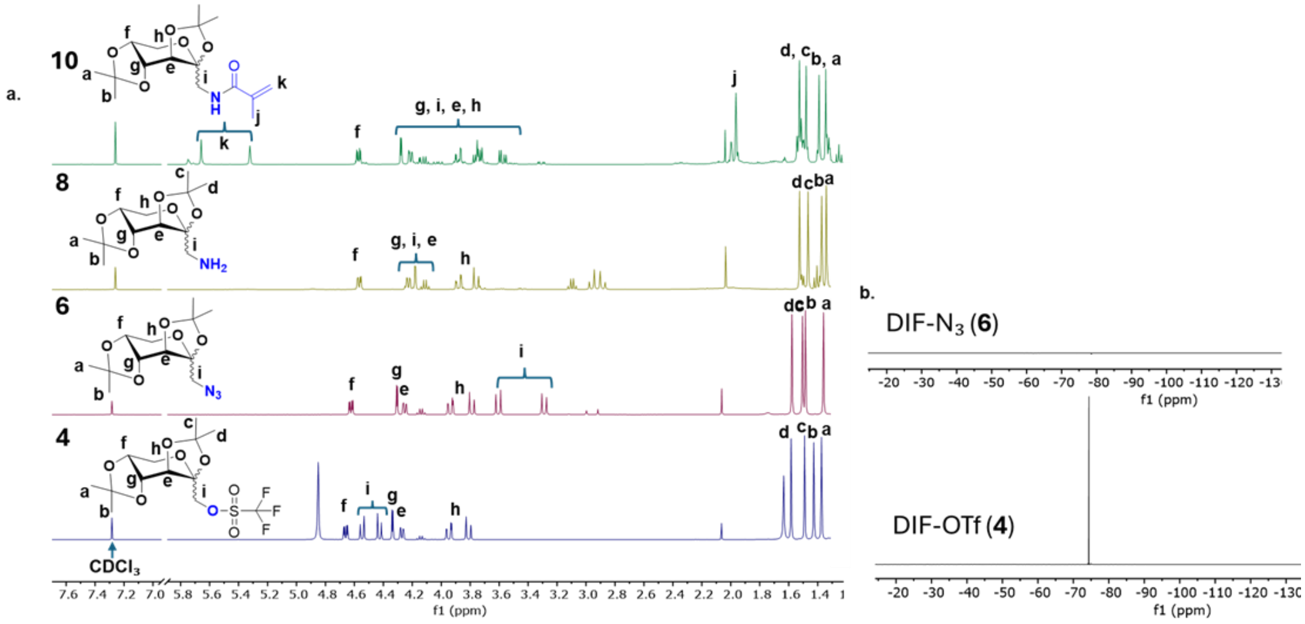

Figure S22. a) ^{1}H NMR of fructose derived compound including the final monomer **10** (**M2**). $CDCl_3$ was used as NMR solvent. **b)** ^{19}F NMR (performed in $CDCl_3$) of **4** and **6** to compare the presence and absence of fluorine peak between the compounds.

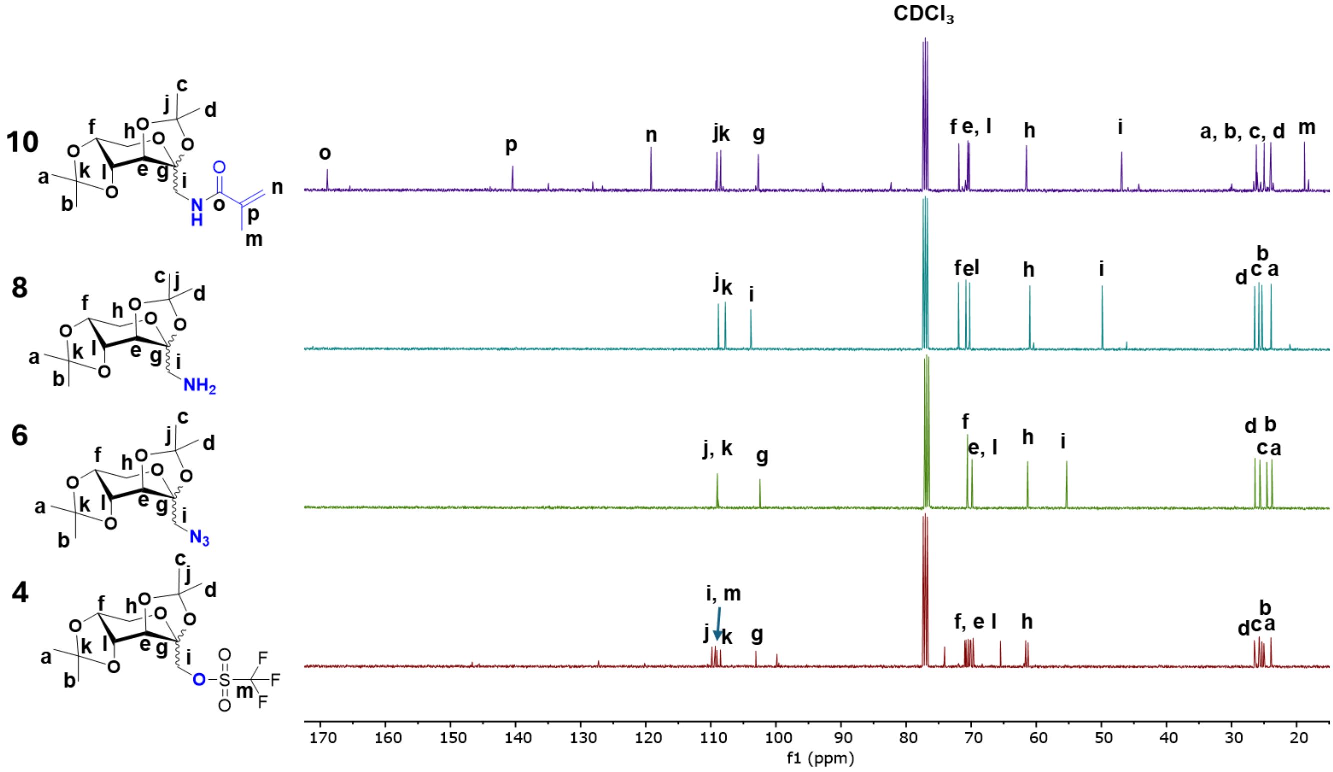

Figure S23. ^{13}C NMR of fructose derived compounds with final monomer **10** (**M2**). $CDCl_3$ was used as NMR solvent.

3.3.4 Aldose monomers (intermediates and final monomers):

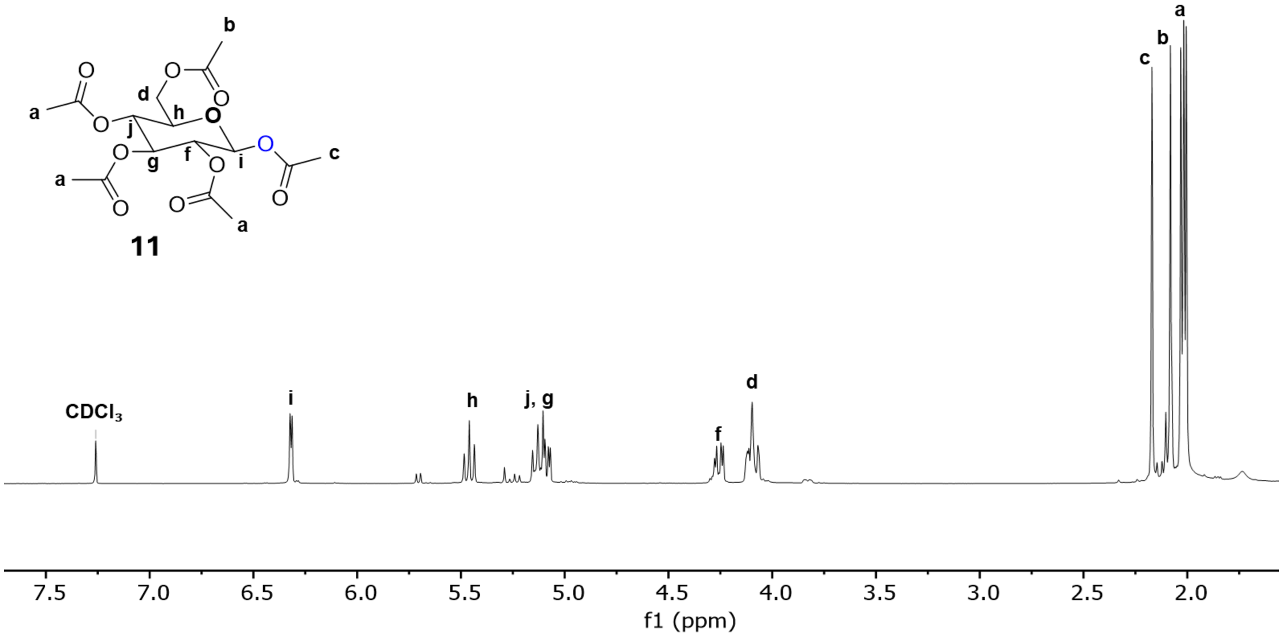

Figure S24. ^{1}H NMR of pentaacetyl-*β*-*D*-glucose (**11**). $CDCl_3$ was used as NMR solvent.

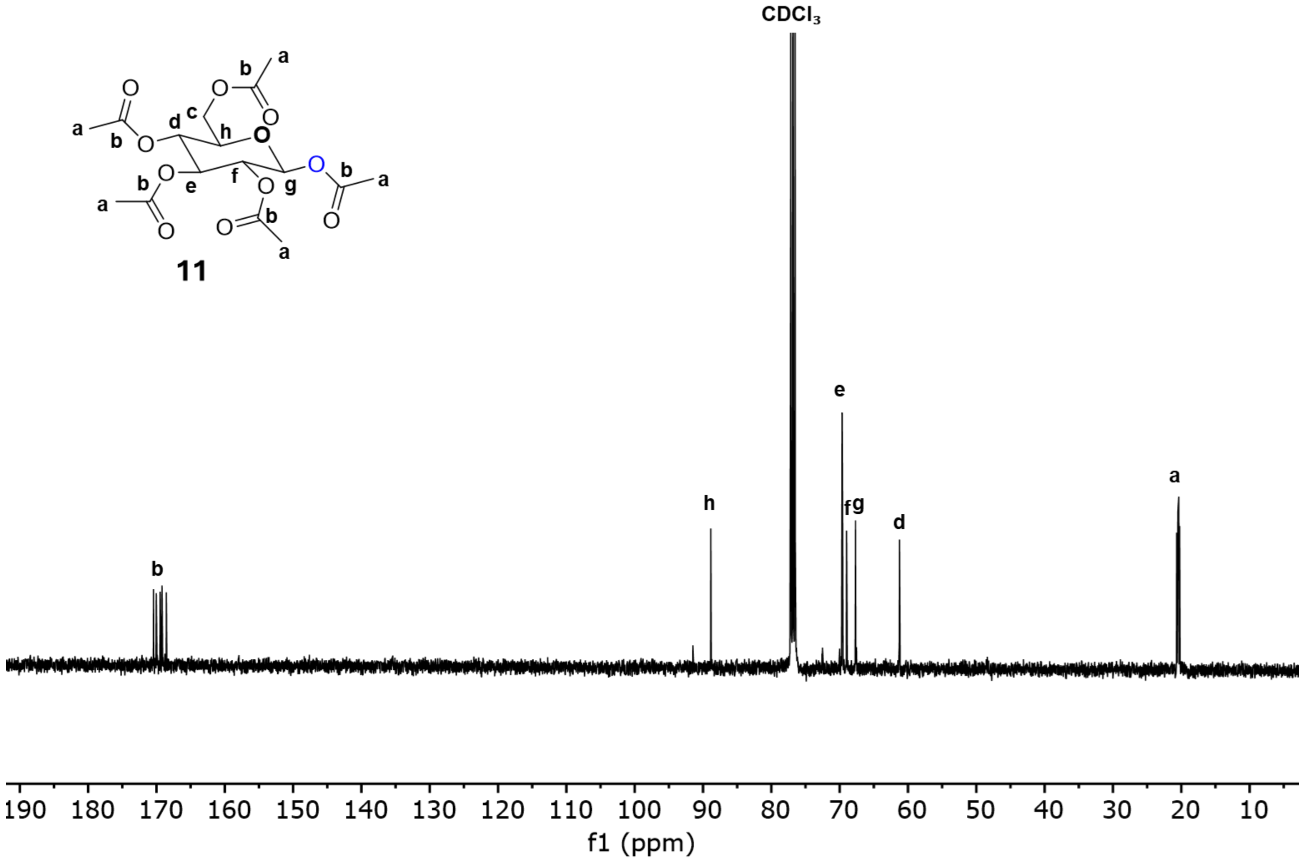

Figure S25. ^{13}C NMR of pentaacetyl-*β*-*D*-glucose (**11**). $CDCl_3$ was used as NMR solvent.

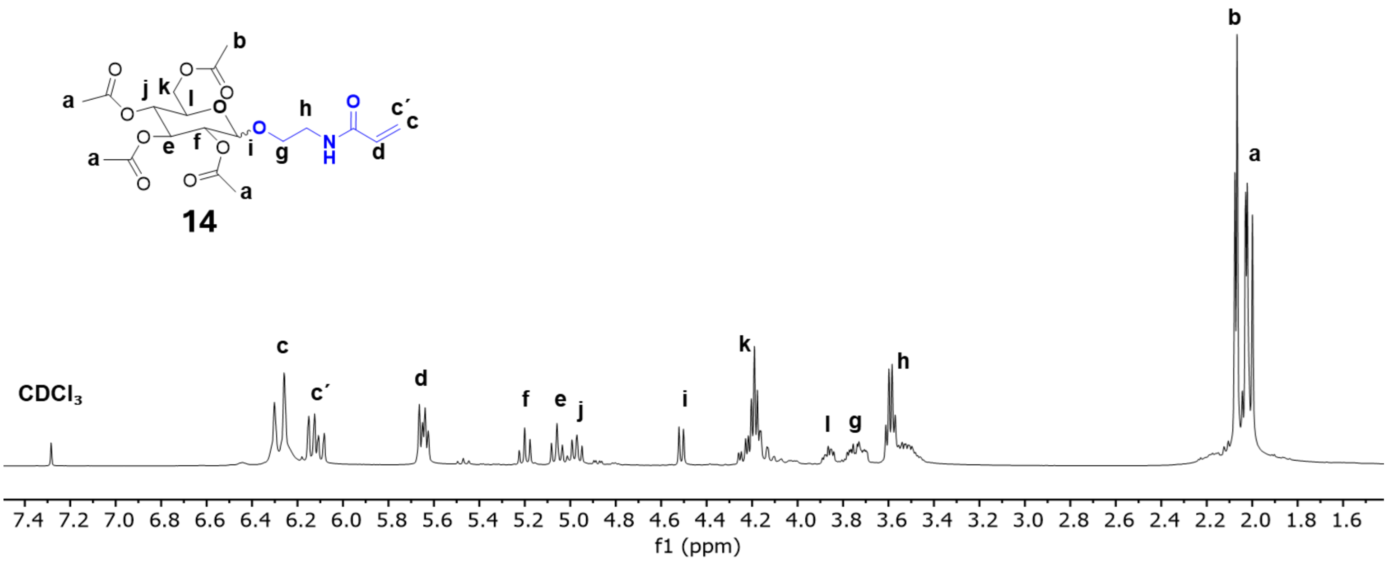

Figure S26. ^{1}H NMR of GlucosEAm (**14, M3**). $CDCl_3$ was used as NMR solvent.

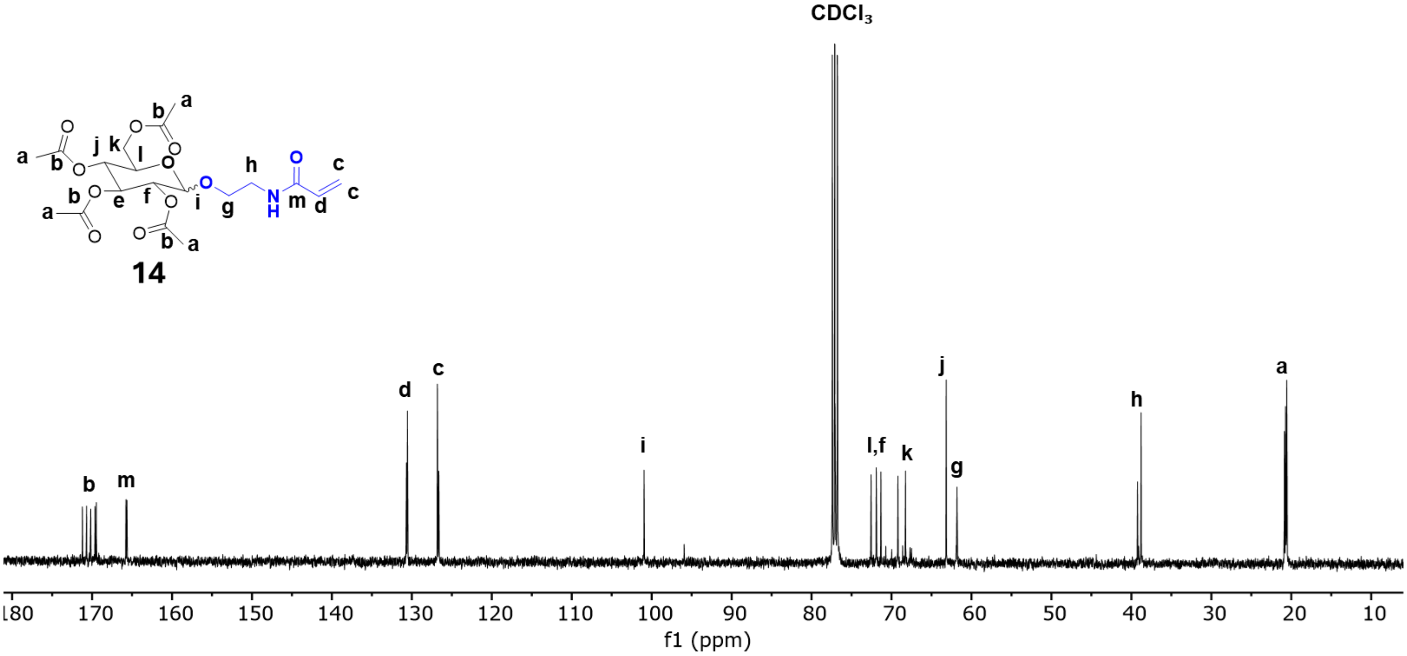

Figure S27. ^{13}C NMR of GlucosEAm (**14, M3**). $CDCl_3$ was used as NMR solvent.

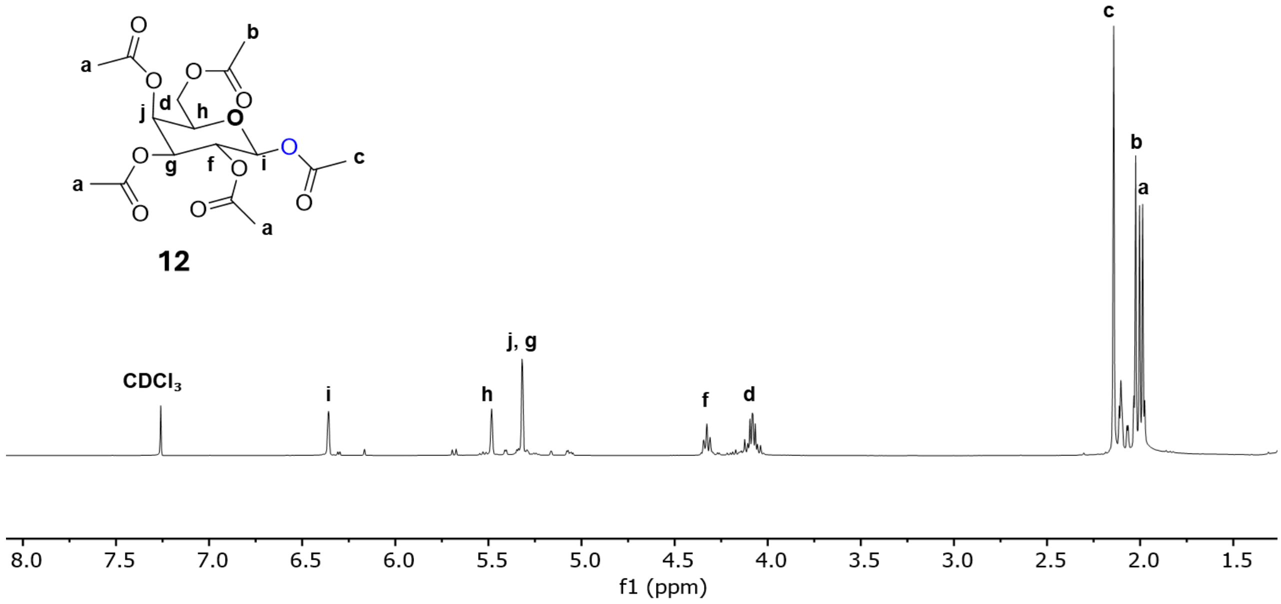

Figure S28. ^{1}H NMR of pentaacetyl-*β*-*D*-galactose (**12**). $CDCl_3$ was used as NMR solvent.

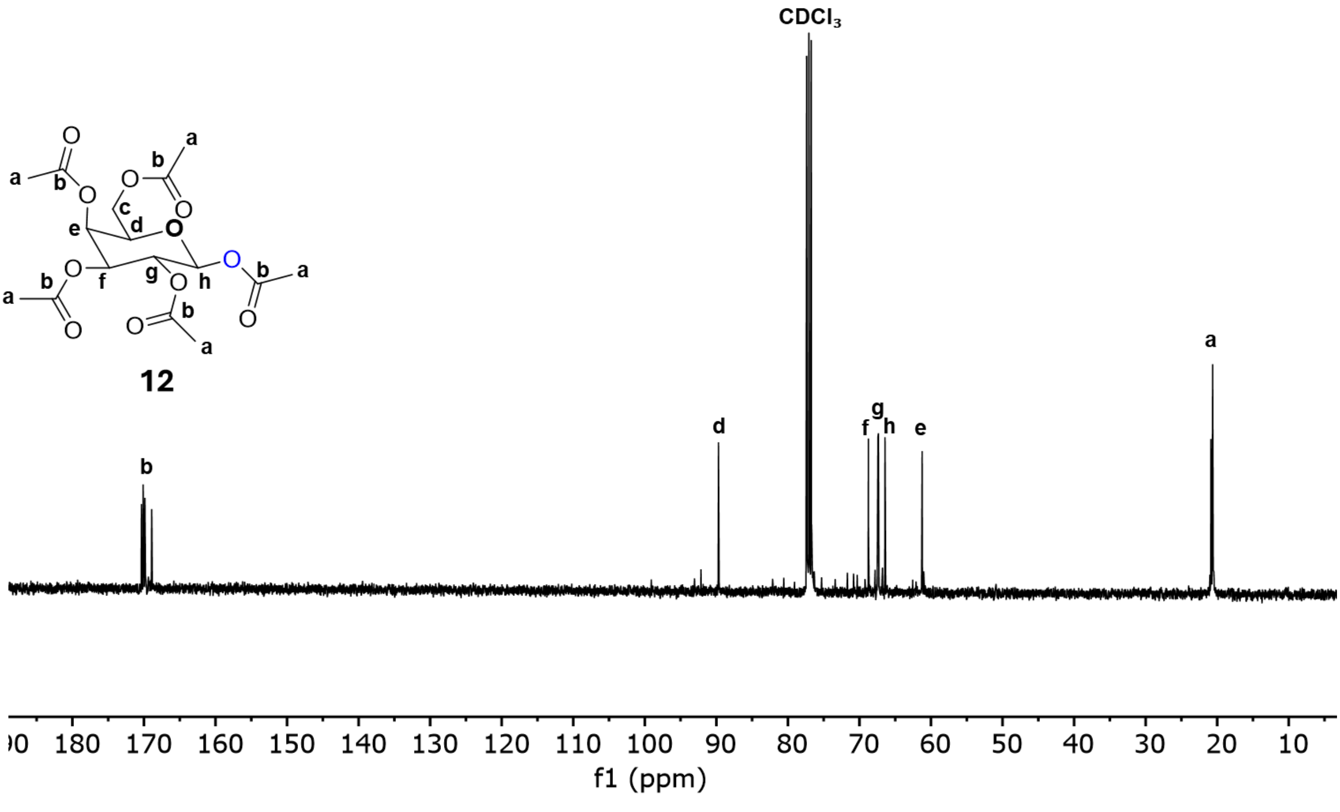

Figure S29. ^{13}C NMR of pentaacetyl-*β*-*D*-galactose (**12**). $CDCl_3$ was used as NMR solvent.

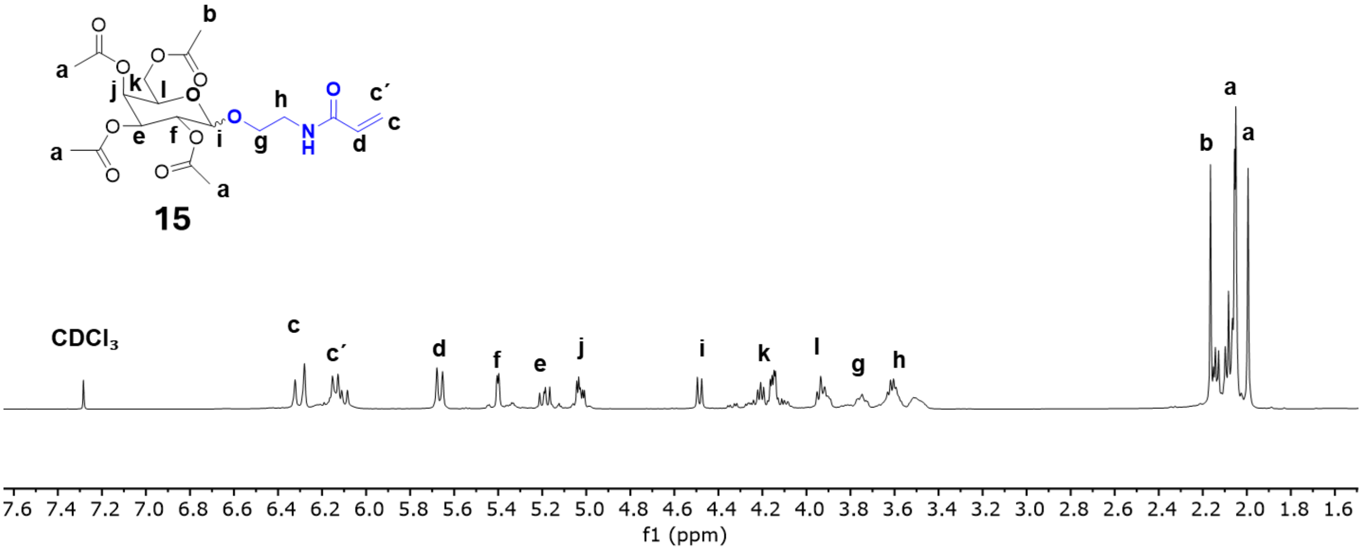

Figure S30. ^{1}H NMR of GalactosEAm (**15, M4**). $CDCl_3$ was used as NMR solvent.

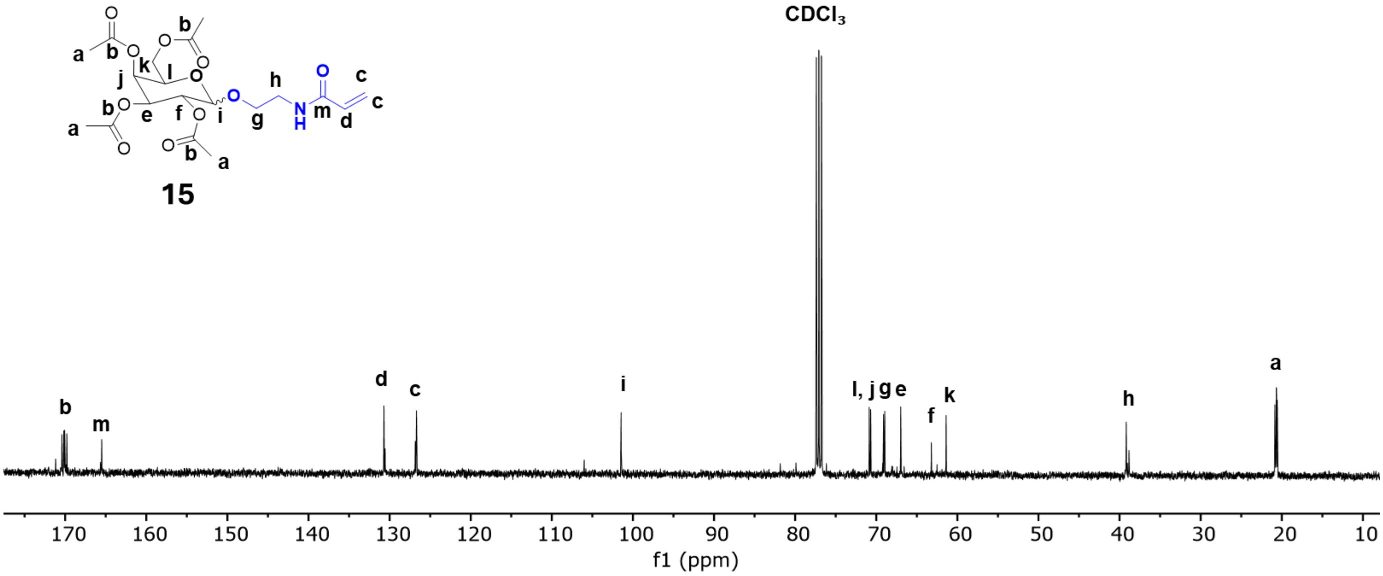

Figure S31. ^{13}C NMR of GalactosEAm (**15, M4**). $CDCl_3$ was used as NMR solvent.

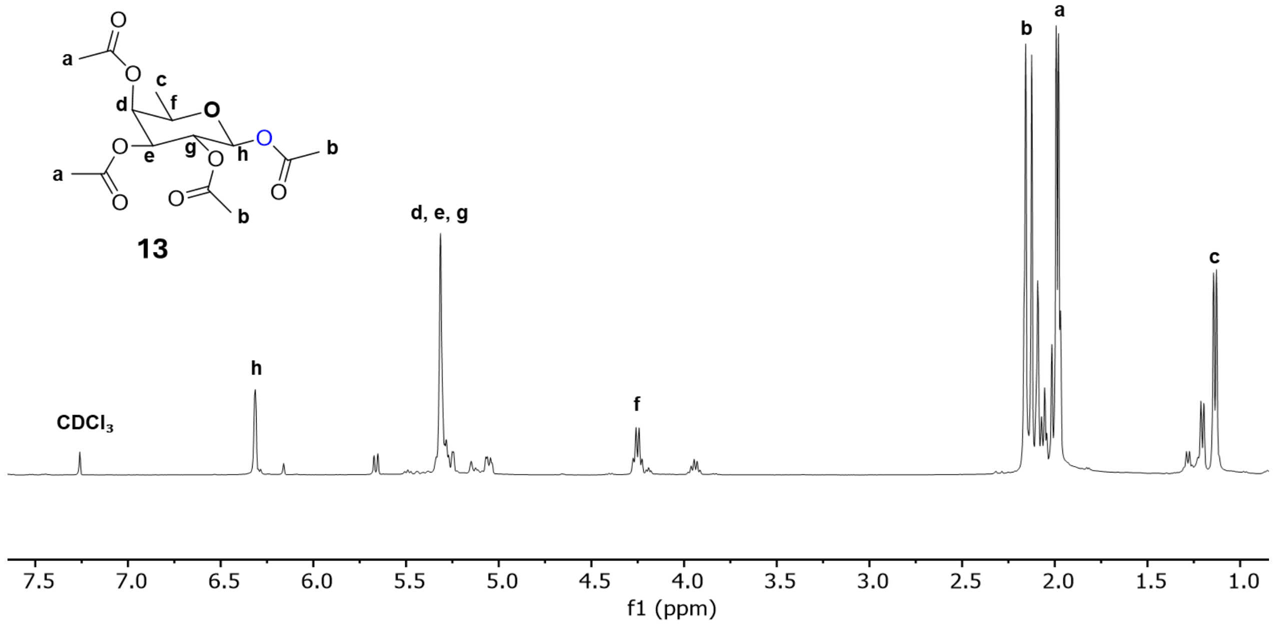

Figure S32. ^{1}H NMR of tetraacetyl-*α*-*L*-fucose (**13**). $CDCl_3$ was used as NMR solvent.

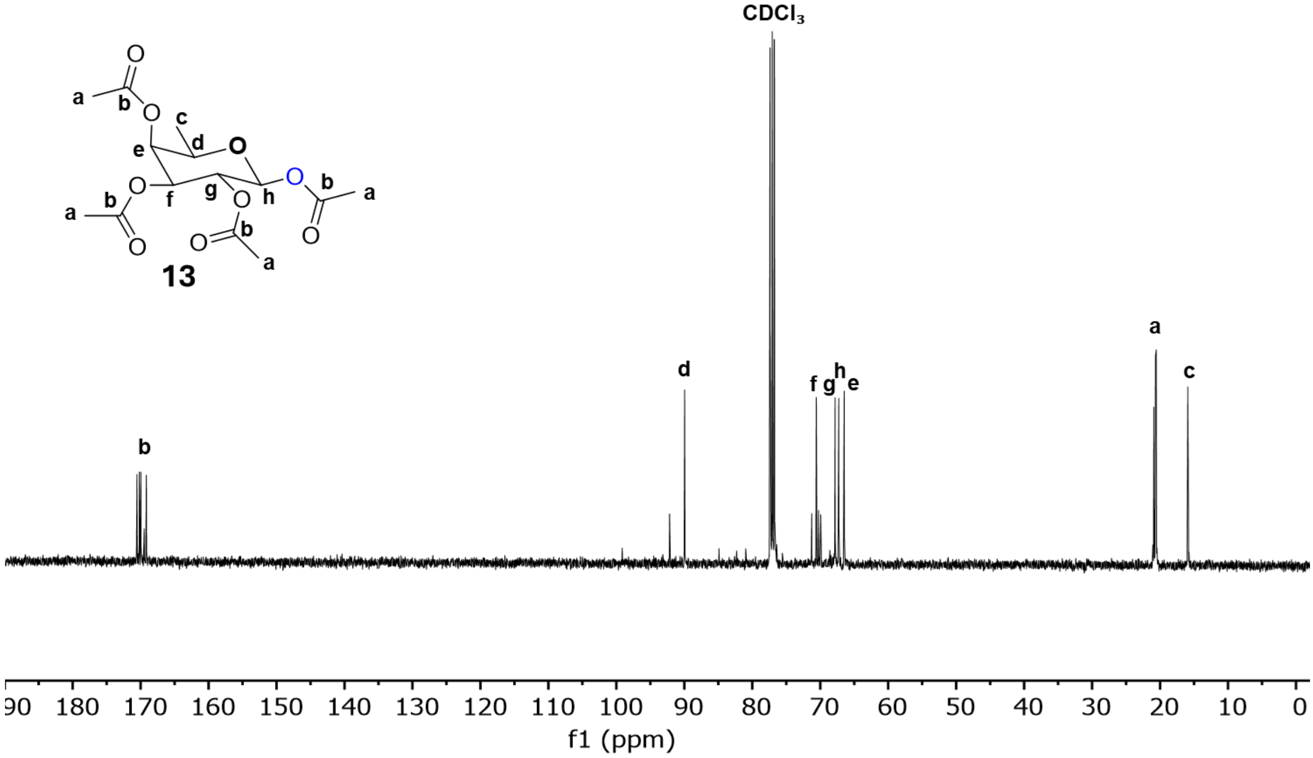

Figure S33. ^{13}C NMR of tetraacetyl-*α*-*L*-fucose (**13**). $CDCl_3$ was used as NMR solvent.

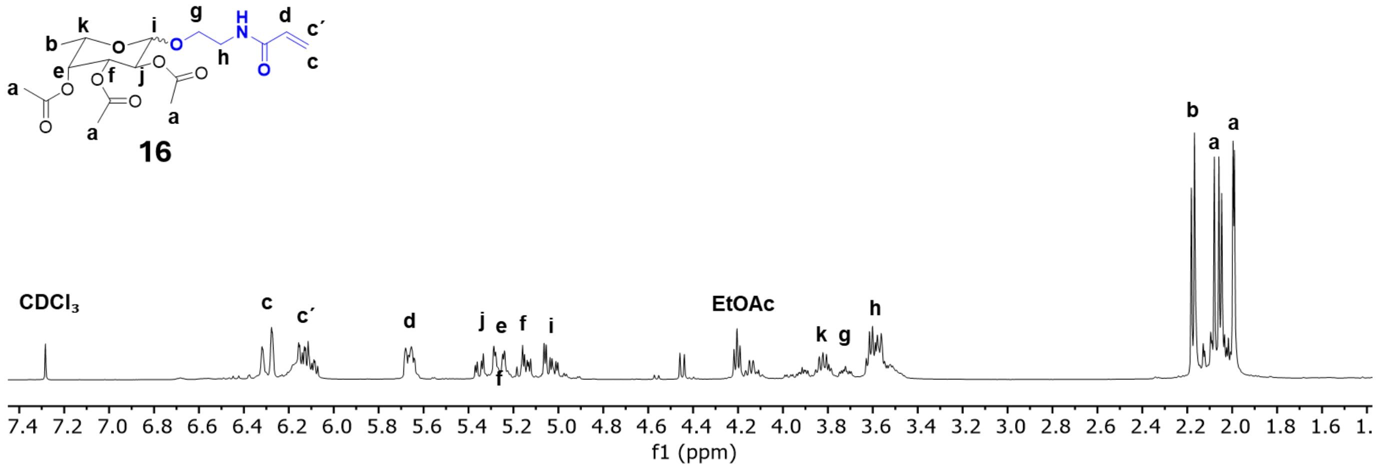

Figure S34. ^{1}H NMR of FucosEAm (**16, M5**). CDCl$_3$ was used as NMR solvent.

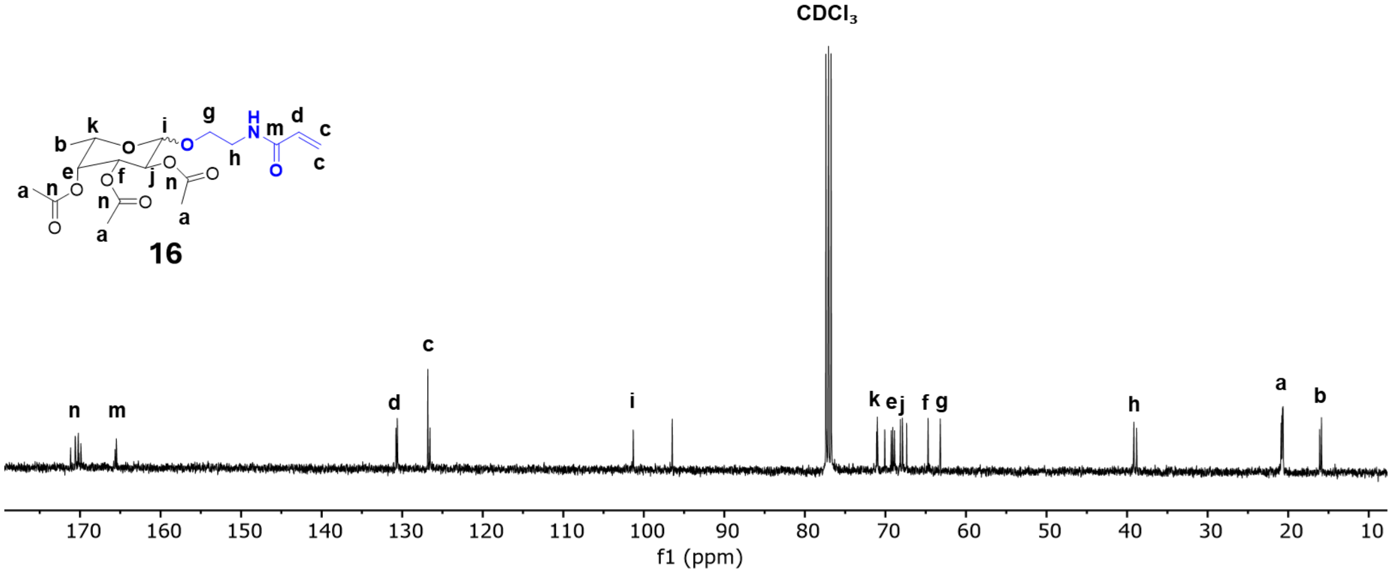

Figure S35. ^{13}C NMR of FucosEAm (**16, M5**). CDCl$_3$ was used as NMR solvent.

3.3.5 Crystallographic Data:

Table S1. Crystal data and details of structure refinement.

Compound	**DIF-N_3 (6)**	**DIFMAm (10, M2)**	**DIS-OTf (3)**	**DIS-N_3 (5)**
CCDC number	2393133	2393131	2393174	2393305
Empirical formula	$C_{12}H_{19}N_3O_5$	$C_{16}H_{25}NO_6$	$C_{13}H_{19}F_3O_8S$	$C_{12}H_{19}N_3O_5$
Formula weight	285.30	327.37	392.34	285.30
Temperature [K]	293	293	150	240
Crystal system	monoclinic	orthorhombic	orthorhombic	tetragonal
Space group (number)	$C2$ (5)	$P2_12_12_1$ (19)	$P2_12_12_1$ (19)	$P4_1$ (76)
a [Å]	20.642(4)	9.7416(19)	10.756(2)	11.0819(16)
b [Å]	7.1090(14)	9.861(2)	15.709(3)	11.0819(16)
c [Å]	12.234(2)	17.762(4)	20.719(4)	11.624(2)
α [°]	90	90	90	90
β [°]	124.83(3)	90	90	90
γ [°]	90	90	90	90
Volume [Å^3]	1473.7(7)	1706.3(6)	3500.8(12)	1427.5(5)
Z	4	4	8	4
ρ_{calc} [gcm^{-3}]	1.286	1.274	1.489	1.328
μ [mm^{-1}]	0.101	0.097	0.250	0.103
F(000)	608	704	1632	608
Crystal size [mm^3]	0.100×0.233×0.300	0.100×0.133×0.200	0.025×0.158×0.400	0.025×0.158×0.400
Crystal colour	colorless	colorless	colorless	colorless
Crystal shape	block	plate	plate	plate
Radiation	MoK_α (λ=0.71073 Å)	MoK_α (λ=0.71073 Å)	MoK_α (λ=0.71073 Å)	MoK_α (λ=0.71073 Å)
2θ range [°]	6.66 to 63.92 (0.67 Å)	7.46 to 64.69 (0.66 Å)	6.42 to 55.00 (0.77 Å)	7.35 to 49.99 (0.84 Å)
Index ranges	$-29 \le h \le 29$<br>$-10 \le k \le 8$<br>$-17 \le l \le 17$	$-14 \le h \le 13$<br>$-14 \le k \le 14$<br>$-24 \le l \le 26$	$-13 \le h \le 13$<br>$-20 \le k \le 19$<br>$-26 \le l \le 26$	$-13 \le h \le 13$<br>$-13 \le k \le 13$<br>$-13 \le l \le 13$
Reflections collected	10047	24040	45763	11894
Independent reflections	3787<br>R_{int} = 0.0464<br>R_{sigma} = 0.0684	5746<br>R_{int} = 0.0565<br>R_{sigma} = 0.0774	8018<br>R_{int} = 0.0353<br>R_{sigma} = 0.0232	2512<br>R_{int} = 0.0541<br>R_{sigma} = 0.0571
Completeness to θ = 25°	99.2 %	99.6 %	99.4 %	99.7 %
Data / Restraints / Parameters	3787 / 1 / 185	5746 / 0 / 216	8018 / 705 / 533	2512 / 1 / 185
Goodness-of-fit on F^2	0.929	0.849	1.047	0.942
Final R indexes [I≥2σ(I)]	R_1 = 0.0680<br>wR_2 = 0.1722	R_1 = 0.0459<br>wR_2 = 0.0832	R_1 = 0.0590<br>wR_2 = 0.1755	R_1 = 0.0436<br>wR_2 = 0.0875
Final R indexes [all data]	R_1 = 0.1398<br>wR_2 = 0.2045	R_1 = 0.1096<br>wR_2 = 0.0968	R_1 = 0.0650<br>wR_2 = 0.1809	R_1 = 0.0775<br>wR_2 = 0.0967
Largest peak/hole [eÅ^{-3}]	0.26/−0.24	0.14/−0.18	0.84/−0.50	0.14/−0.15

3.3.6 Crystal structures of Intermediate compounds of ketose monomers:

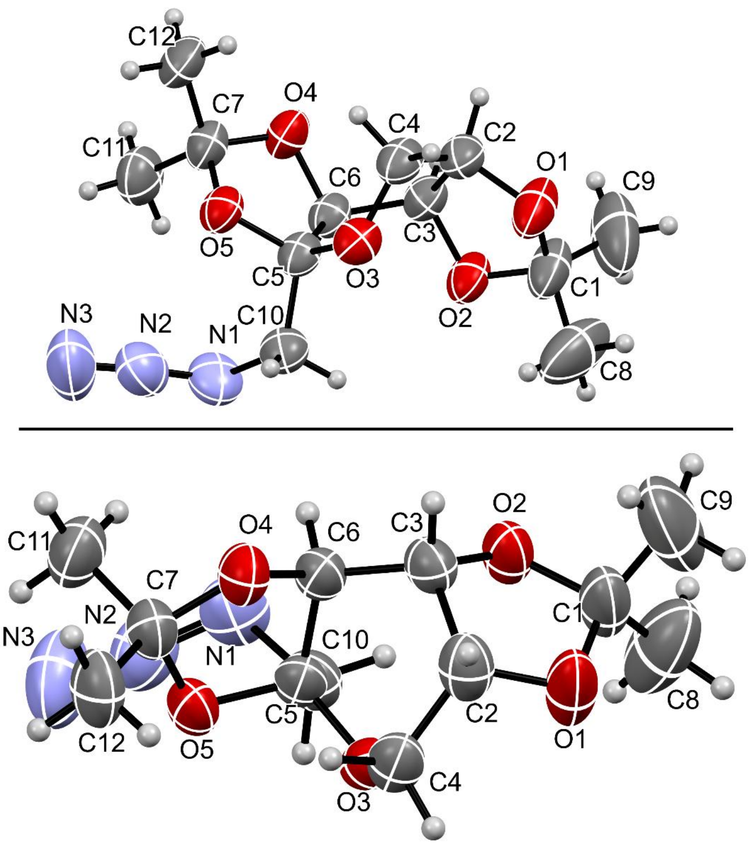

Figure S36. Molecular structure of DIF-N_3 (**6**) with atomic labels in two different viewing positions. Displacement ellipsoids are shown at the 50% probability level.

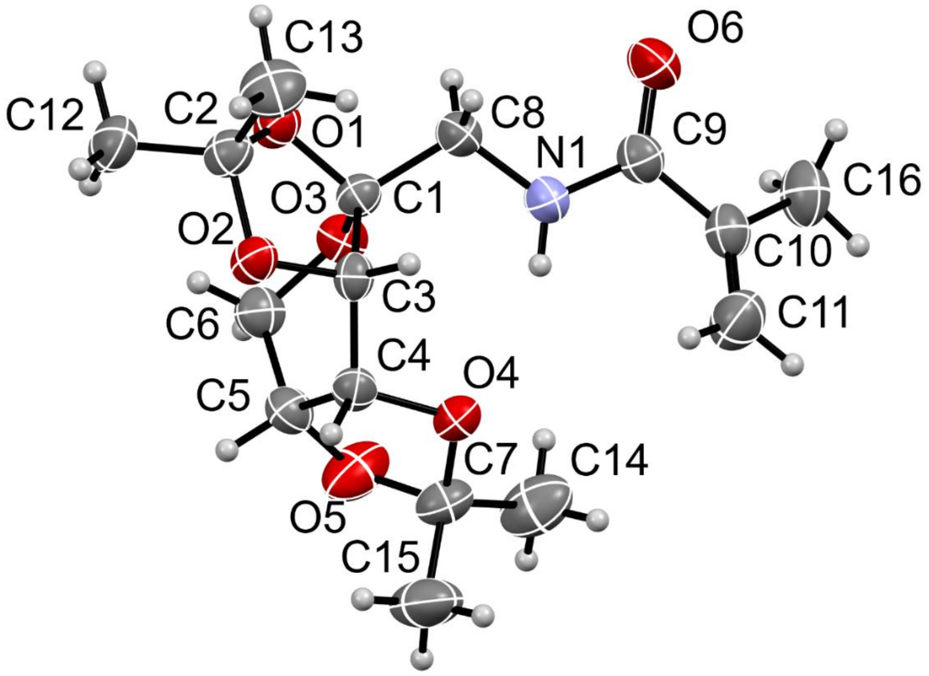

Figure S37. Molecular structure of DIFMAm (**10, M2**) with atomic labels of both symmetry-independent molecules in the asymmetric unit. Displacement ellipsoids are shown at the 50% probability level. This structure is also shown in **Figure 3**.

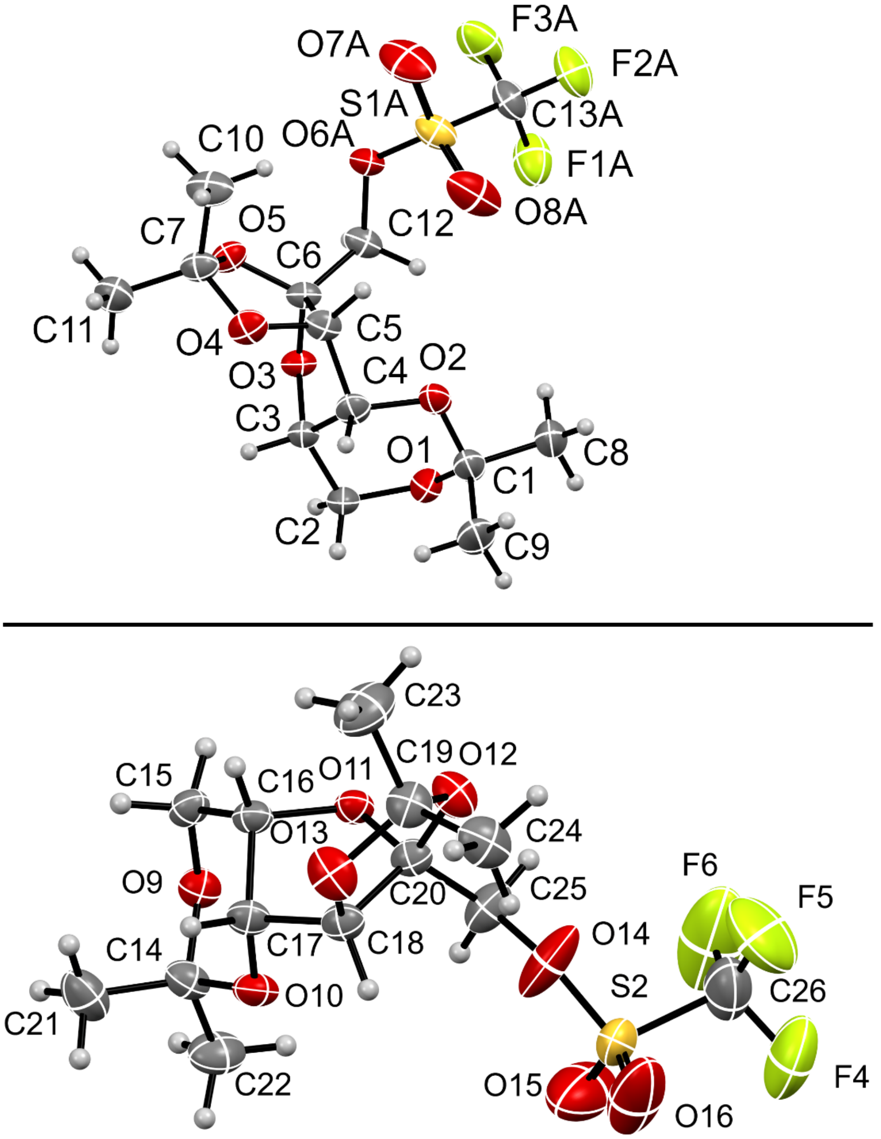

Figure S38. Molecular structure of DIS-OTf (**3**) with atomic labels of both symmetry-independent molecules in the asymmetric unit. Displacement ellipsoids are shown at the 50% probability level.

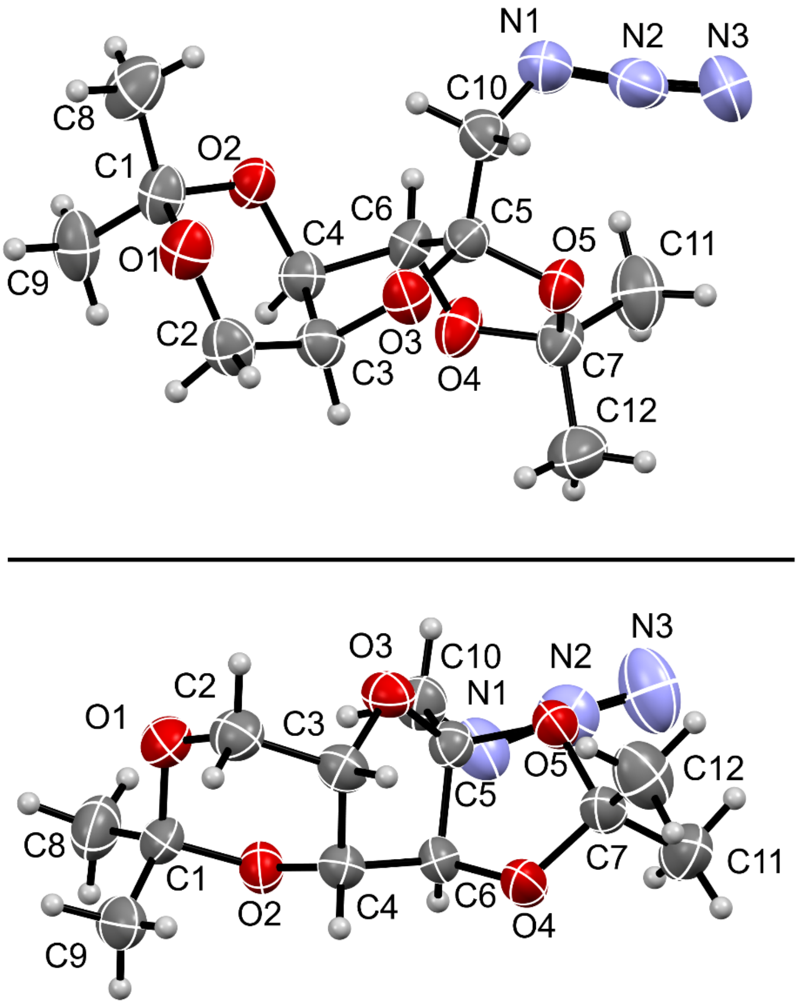

Figure S39. Molecular structure of DIS-N_3 (**5**) with atomic labels in two different viewing positions. Displacement ellipsoids are shown at the 50% probability level (also shown in **Figure 3**).

3.3.7 Mass Spectra of the synthesized intermediates and final ketose and aldose monomers:

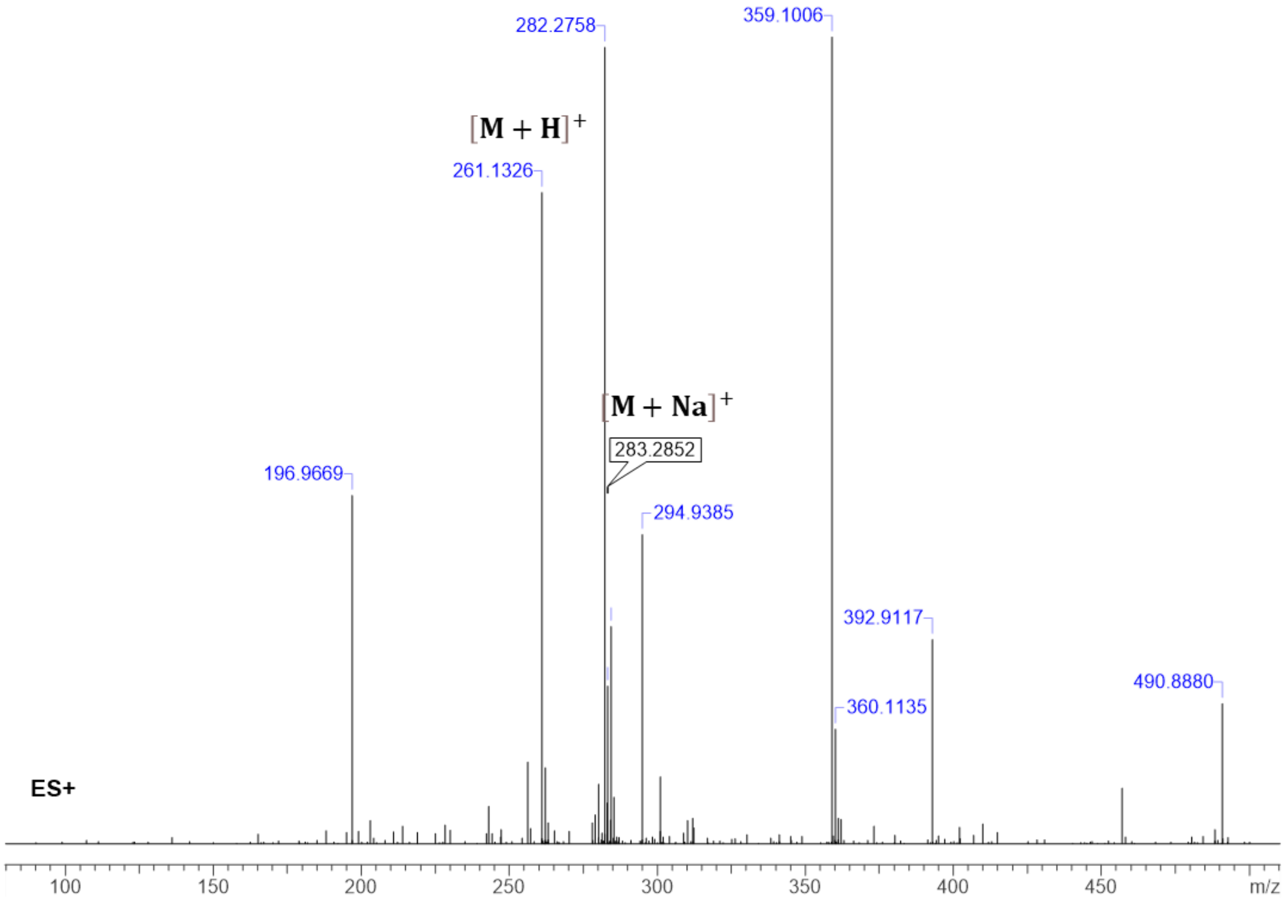

MS (ESI): calculated for $C_{12}H_{20}O_6H$ $[M + H]^+$, 261.1372; found 261.1326; calculated for $C_{12}H_{20}O_6Na$ $[M + Na]^+$, 283.1192; found 282.2758, 283.2852; calculated for ($2\times C_6H_{12}O_6$), 360.1267; found 359.1006, 360.1135

Figure S40. Mass spectra of DIS (**1**) analyzed in CH_3CN.

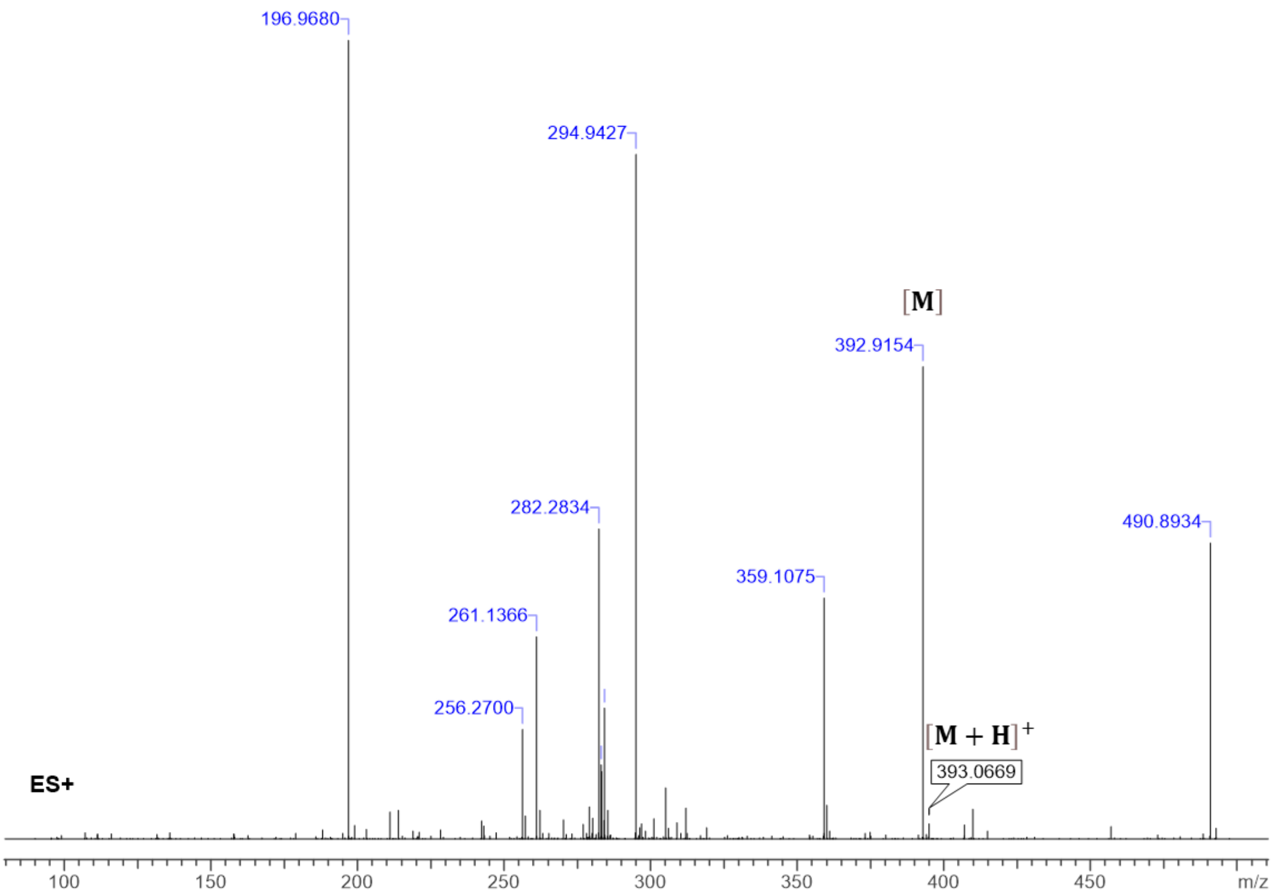

MS (ESI): calculated for $C_{13}H_{19}F_3O_8SH$ [M], 392.0752, found 392.9154; calculated for $C_{13}H_{19}F_3O_8SH$ $[M + H]^+$, 393.0831 ; found 393.0669; 392.9154, found 490.8934, calculated for ($2{\times}C_6H_{12}O_6$), 360.1267; found 359.1075

Figure S41. Mass spectra of DIS-OTf (**3**) analyzed in CH_3CN.

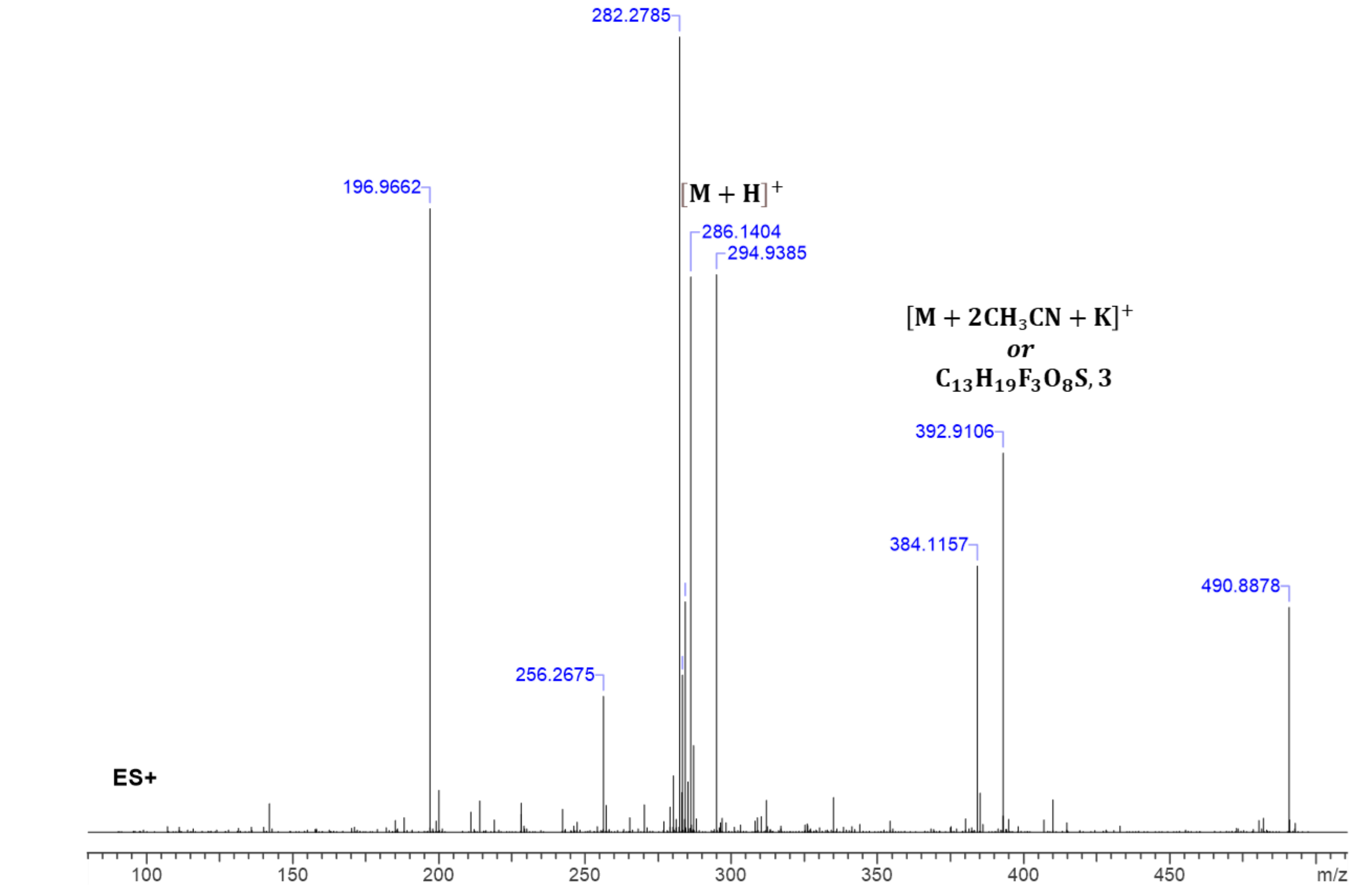

MS (ESI): calculated for $C_{12}H_{19}N_3O_5H$ [M + H]$^+$, 286.1403; 286.1404; found 282.2785; found 294.9385; calculated for $C_{13}H_{19}F_3O_8S$, 392.0753; calculated for $C_{16}H_{25}N_4O_5K$ [M + 2CH$_3$CN + K]$^+$, 392.1462 ; found 392.9106

Figure S42. Mass spectra of DIS-N$_3$ (**5**) analyzed in CH_3CN.

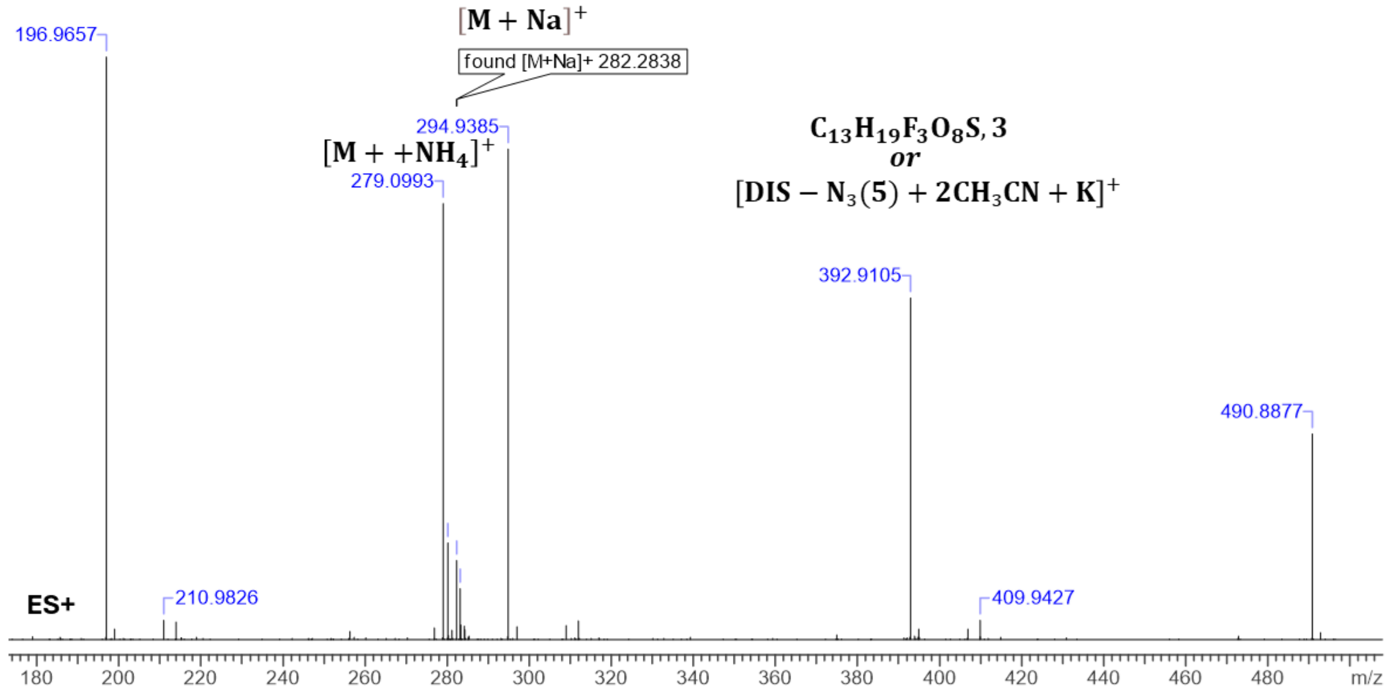

MS (ESI): calculated for $C_{12}H_{21}NO_5Na$ [M + Na]$^+$, 282.1317; found 282.2838; calculated for $C_{12}H_{21}NO_5NH_4$ [M + NH_4]$^+$, 277.1763; found 279.0993; found 294.9385; calculated for $C_{13}H_{19}F_3O_8S$, 392.0753; $C_{16}H_{25}N_4O_5K$ [**DIS-N$_3$** + 2CH_3CN + K]$^+$, 392.1462; found 392.9105

Figure S43. Mass spectra of DIS-NH$_2$ (**7**) analyzed in CH_3CN.

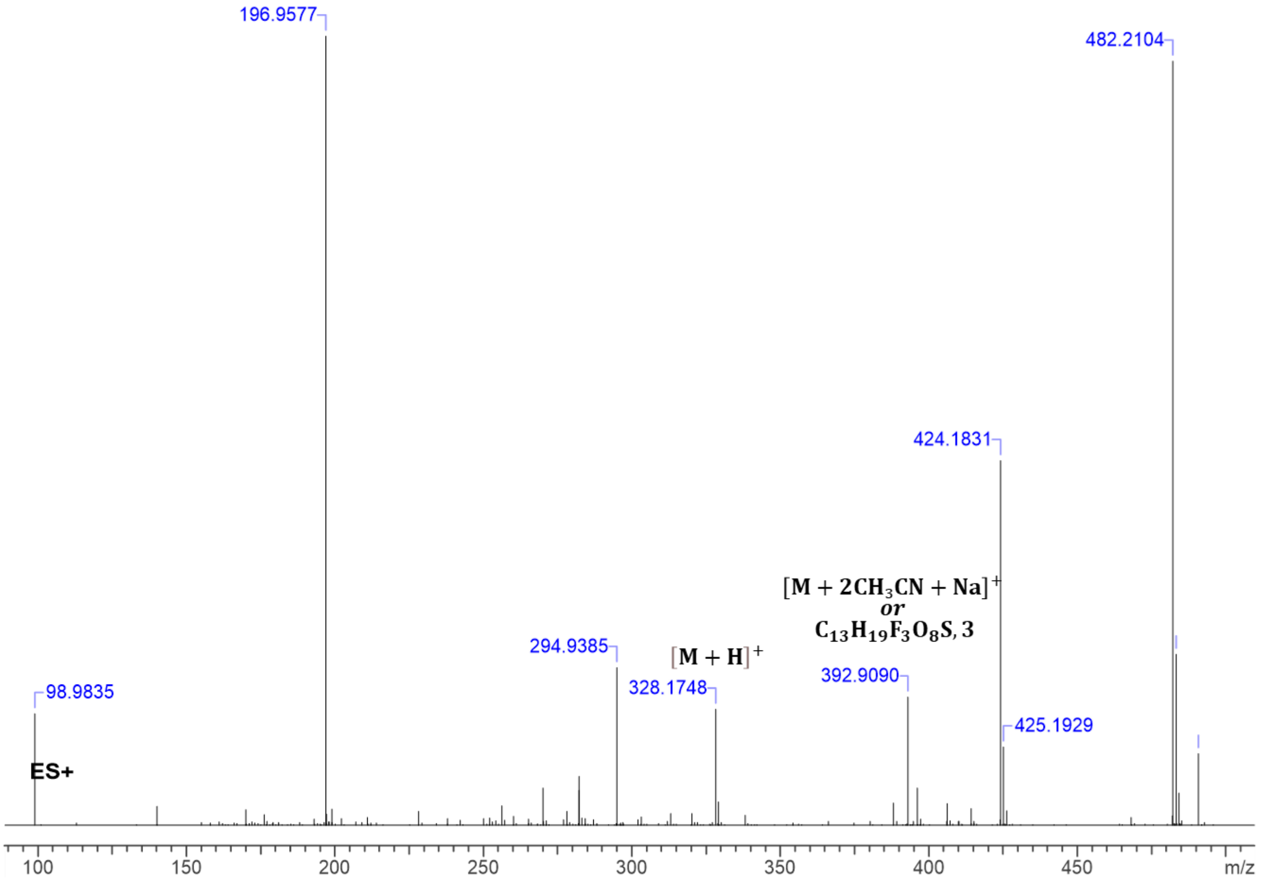

MS (ESI): calculated for $C_{16}H_{25}NO_6H$ [M + H]$^+$, 328.1772; found 328.1748; calculated for $C_{18}H_{28}N_2O_6Na$ [M + CH_3CN + Na]$^+$, 391.1845; calculated for $C_{13}H_{19}F_3O_8S$, 392.0753; found 392.9109; also found 424.1831; and 482.2104.

Figure S44. Mass spectra of DISMAm (**9, M1**) analyzed in CH_3CN. [M + H]$^+$

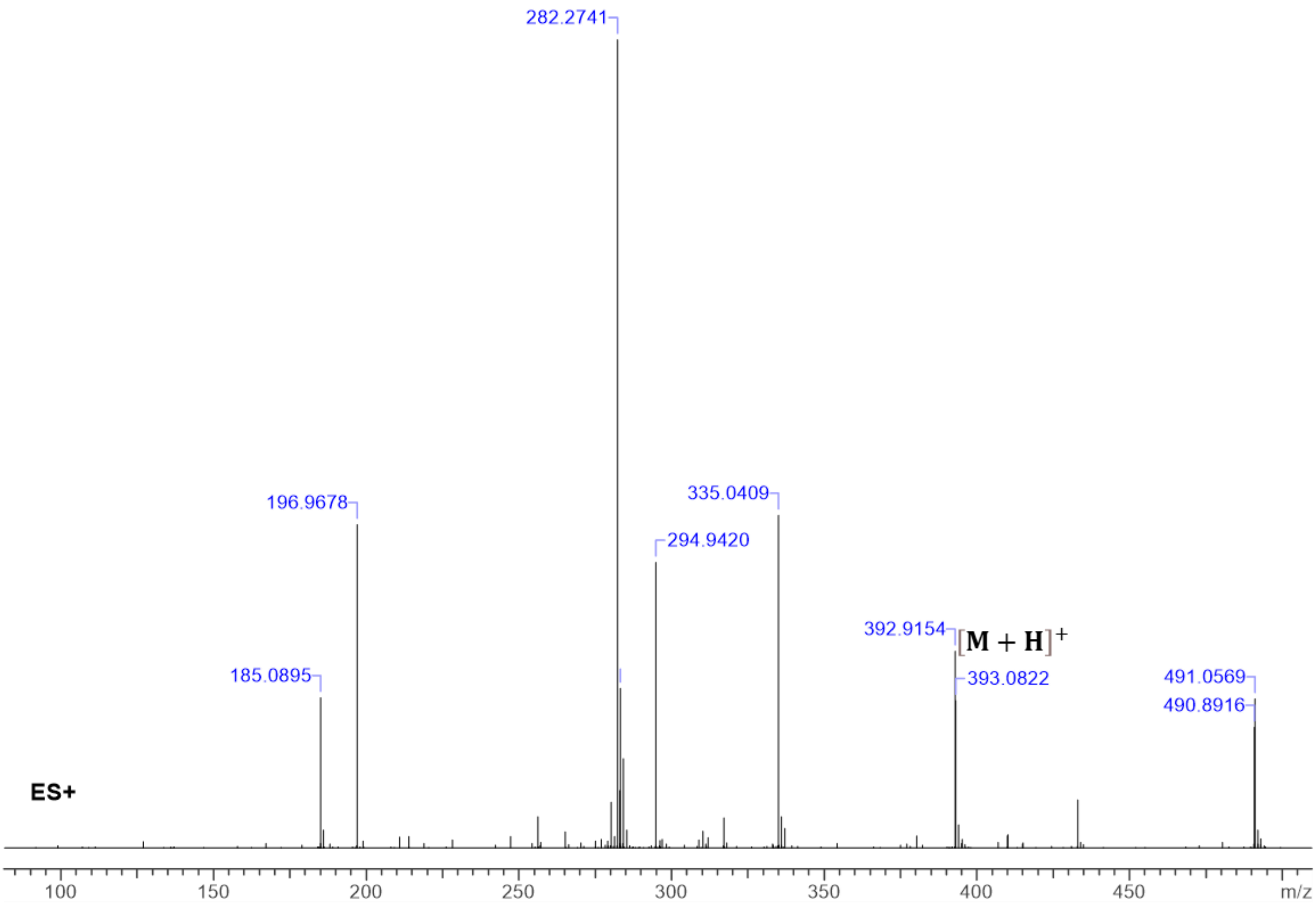

MS (ESI): calculated for $C_{13}H_{19}F_3O_8SH$ $[M + H]^+$ 393.0831; found 393.0822; found 392.0752; found 392.9154; also found 490.8916; and 491.0569

Figure S45. Mass spectra of DIF-OTf (**4**) analyzed in CH_3CN.

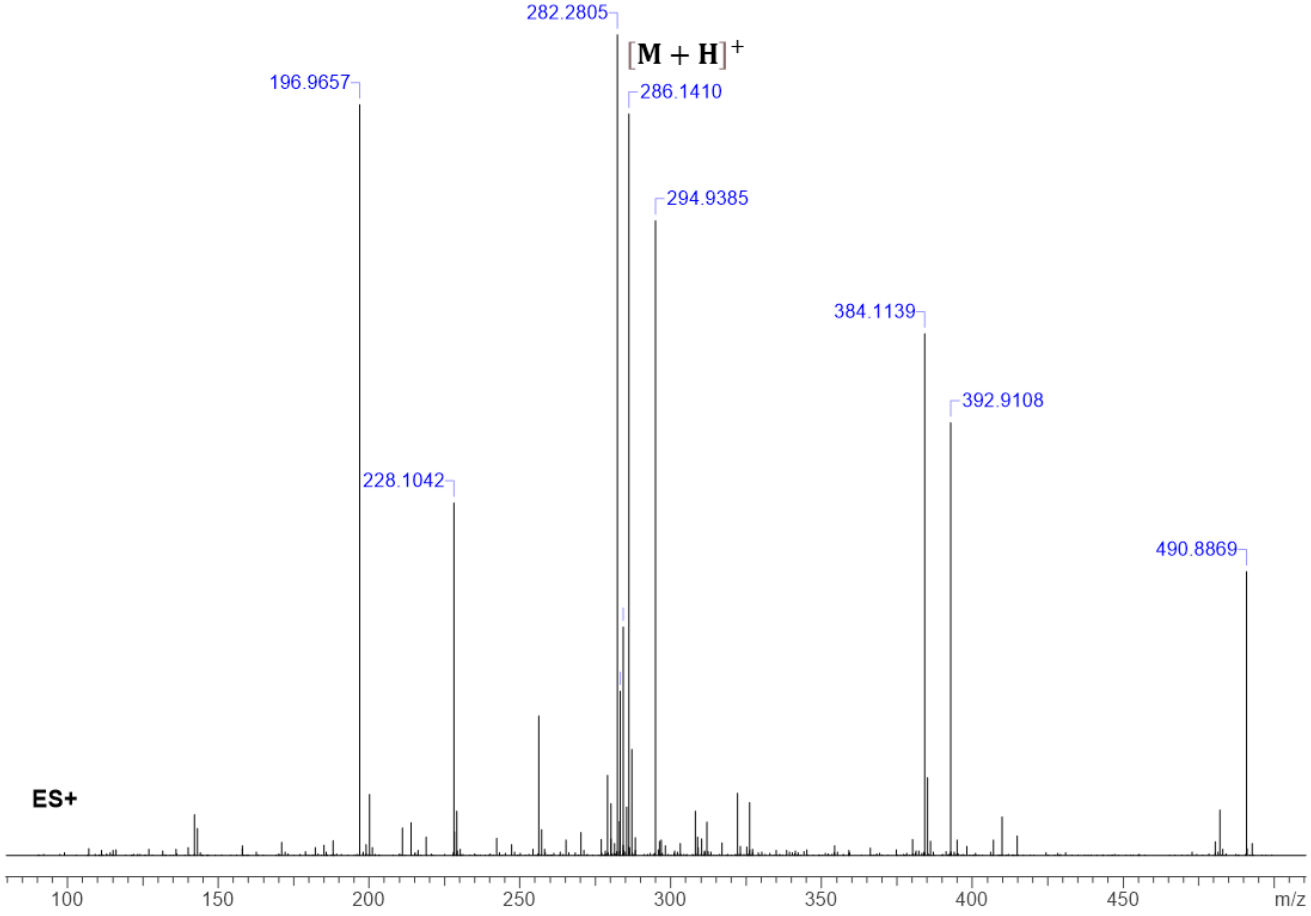

MS (ESI): calculated for $C_{12}H_{19}N_3O_5H$ $[M + H]^+$, 286.1403; found 286.1410; found 282.2805; found 294.9385; calculated for $C_{13}H_{19}F_3O_8S$, 392.0753; found 384.1139; calculated for $C_{16}H_{25}N_4O_5K$ $[M + 2CH_3CN + K]^+$, 392.1462; found 392.9108

Figure S46. Mass spectra of DIF-N_3 (**6**) analyzed in CH_3CN.

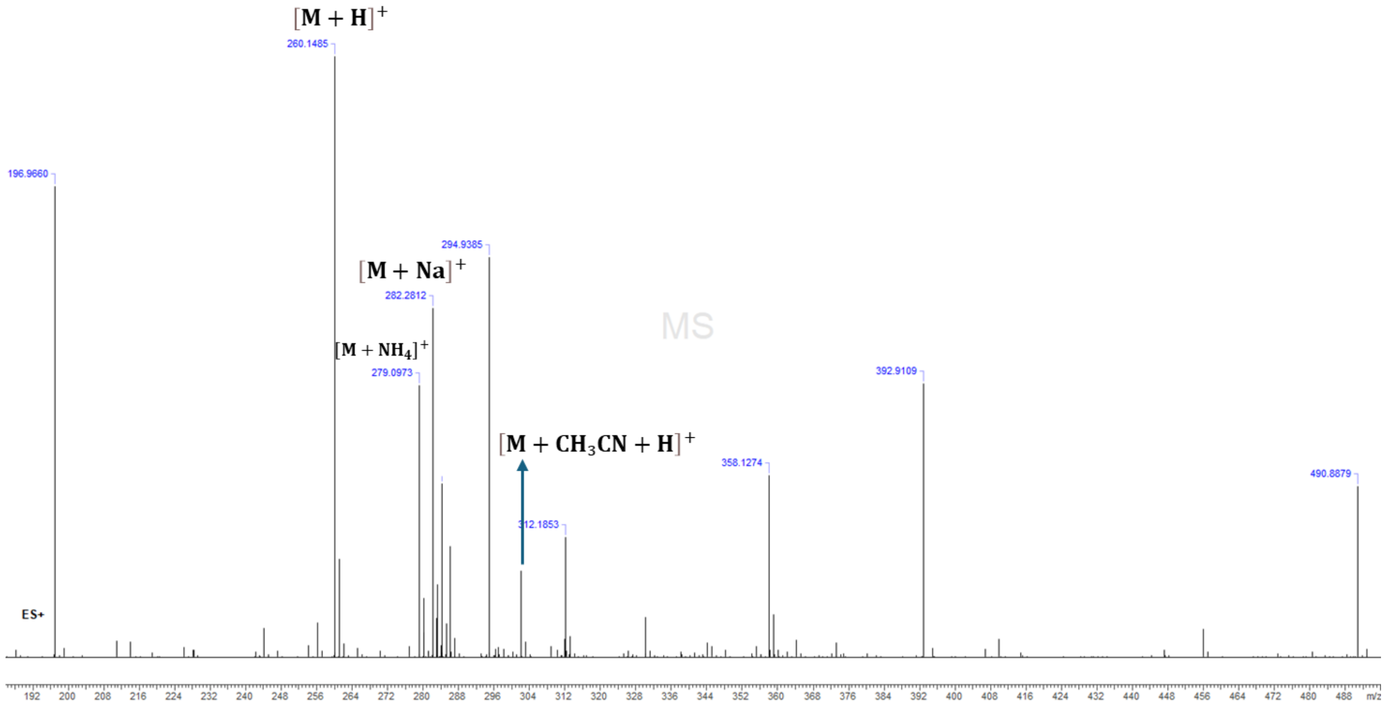

MS (ESI): calculated for $C_{12}H_{21}NO_5H$ [M + H]$^+$, 260.1498; found 260.1485, calculated for $C_{12}H_{21}NO_5Na$ [M + Na]$^+$, 282.1317; found 282.2812; calculated for $C_{12}H_{21}NO_5NH_4$ [M + NH_4]$^+$, 277.1763; found 279.0973; calculated for $C_{14}H_{24}N_2O_5$ [M + CH_3CN + H]$^+$, 301.1438; found 302.1670; calculated for $C_{13}H_{19}F_3O_8S$, 392.0753; and $C_{16}H_{25}N_4O_5K$ [**DIS-N$_3$** + 2CH_3CN + K]$^+$, 392.1462; found 392.9109

Figure S47. Mass spectra of DIF-NH_2 (**8**) analyzed in CH_3CN.

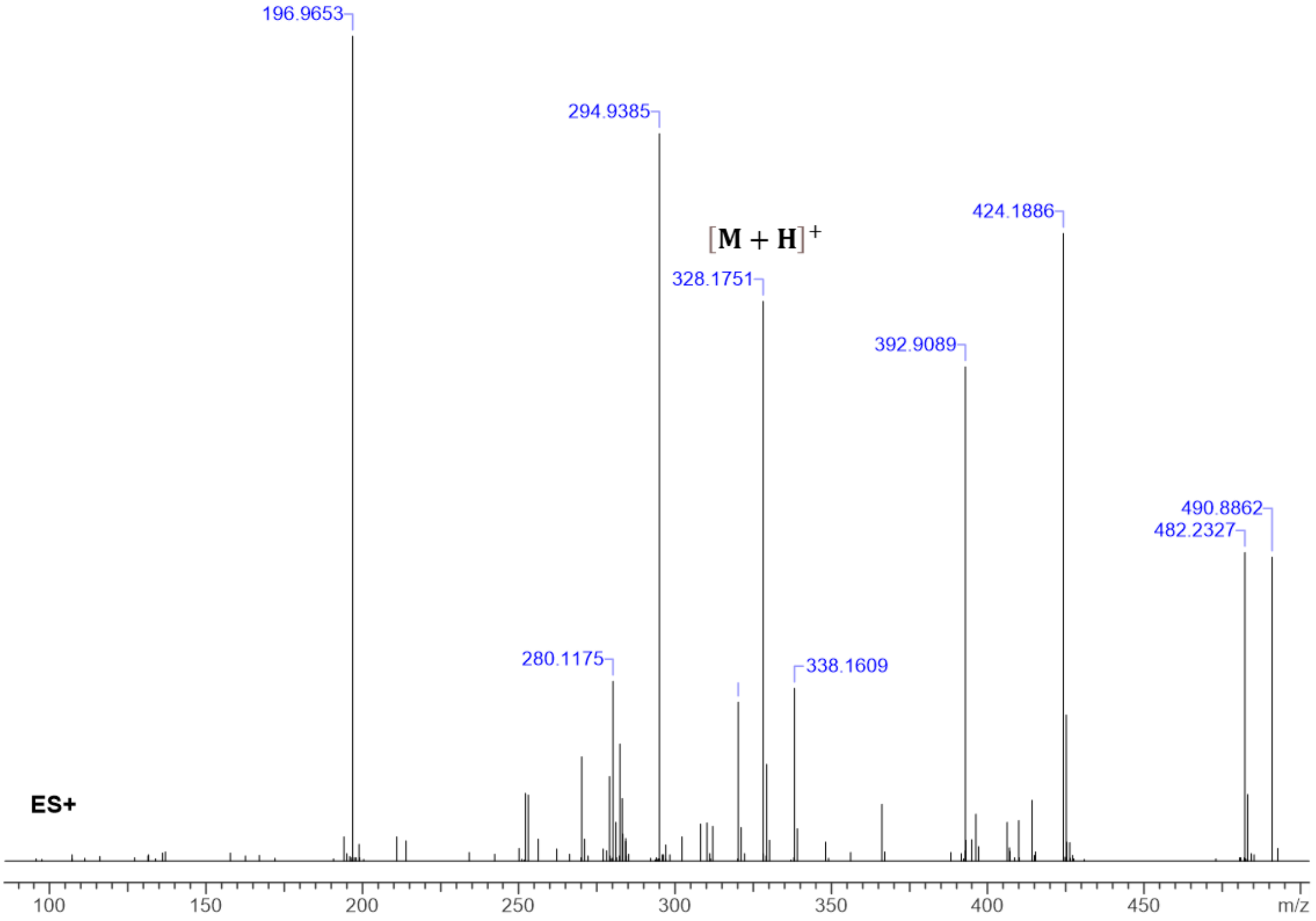

MS (ESI): calculated for $C_{16}H_{25}NO_6H$ [M + H]$^+$, 328.1772; found 328.1751; calculated for $C_{18}H_{28}N_2O_6Na$ [M + CH_3CN + Na]$^+$, 391.1845; calculated for $C_{13}H_{19}F_3O_8S$, 392.0753; found 392.9089, also found 424.1886; 482.2327; and 490.8862

Figure S48. Mass spectra of DIFMAm (**10, M2**) analyzed in CH_3CN.

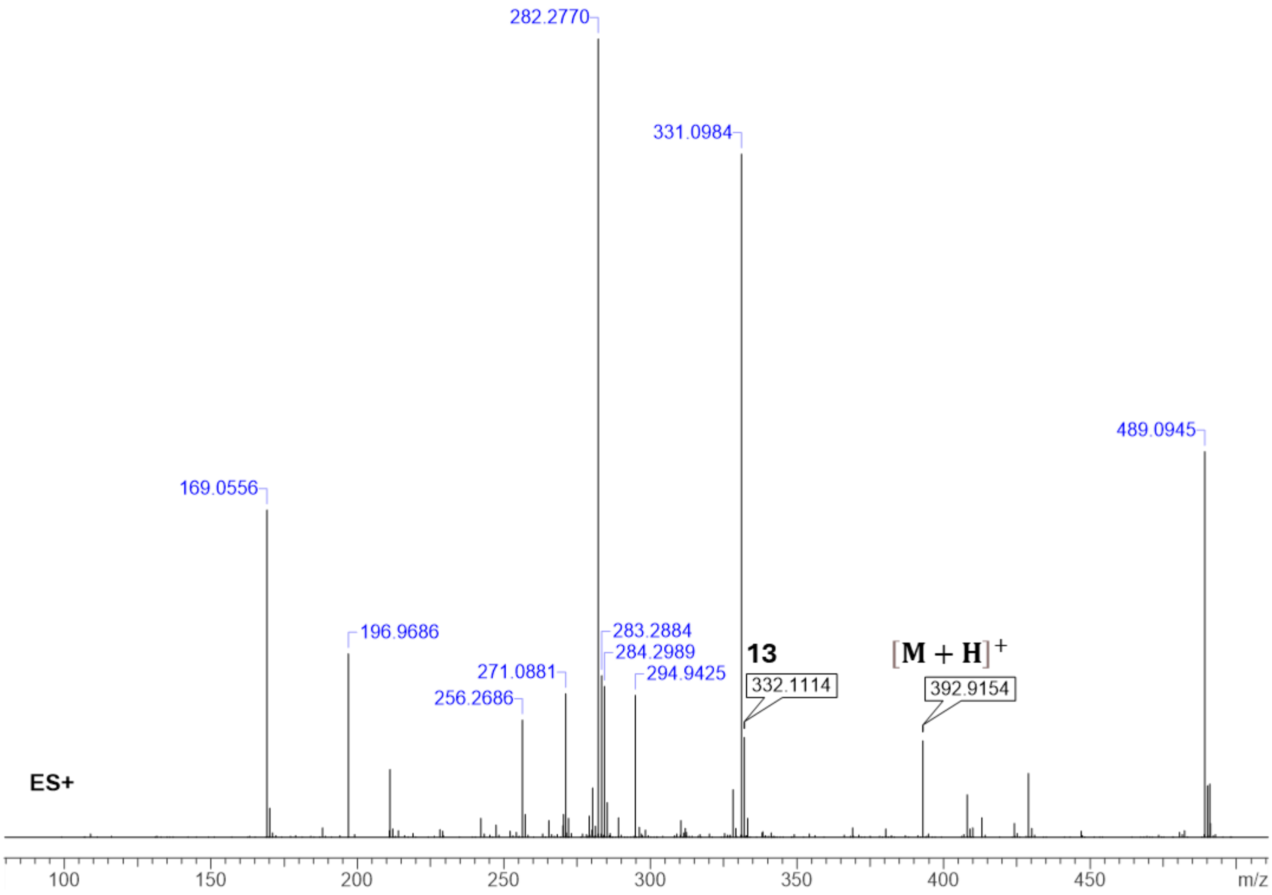

MS (ESI): calculated for $C_{16}H_{22}O_{11}H$ [M + H]$^{+}$, 391.1240; found 392.9154; calculated for $C_{14}H_{20}O_{9}$ (FucoseOAc, **13**), 332.1107; found 332.1114, 331.0984

Figure S49. Mass spectra of pentaacetyl-*β*-*D*-glucose (**11**) analyzed in CH_3CN.

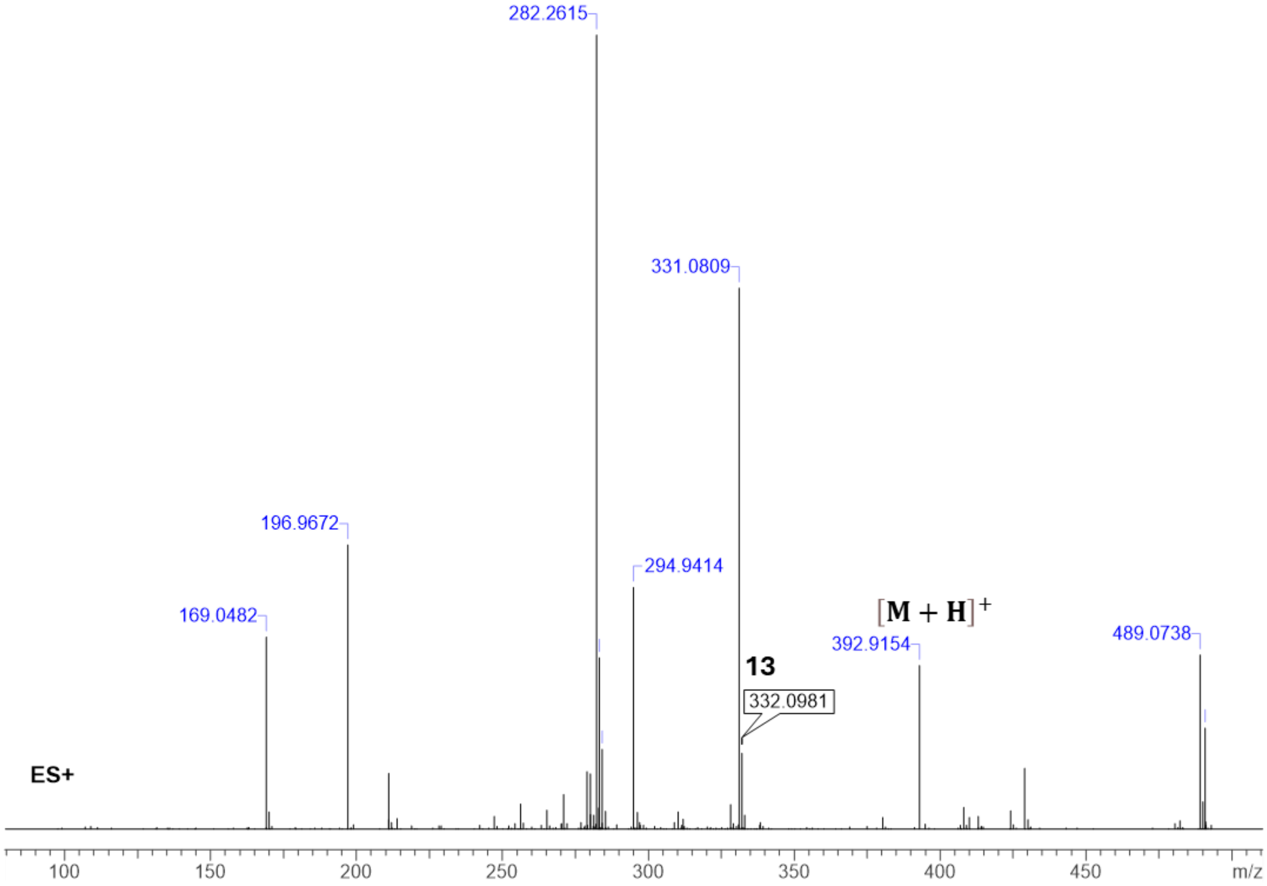

MS (ESI): calculated for $C_{16}H_{22}O_{11}H$ [M + H]$^+$, 391.1240; found 392.9154; calculated for $C_{14}H_{20}O_9$ (FucoseOAc, **13**), 332.1107; found 331.0809; found 332.0981.

Figure S50. Mass spectra of pentaacetyl-*β*-*D*-galactose (**12**) analyzed in CH_3CN.

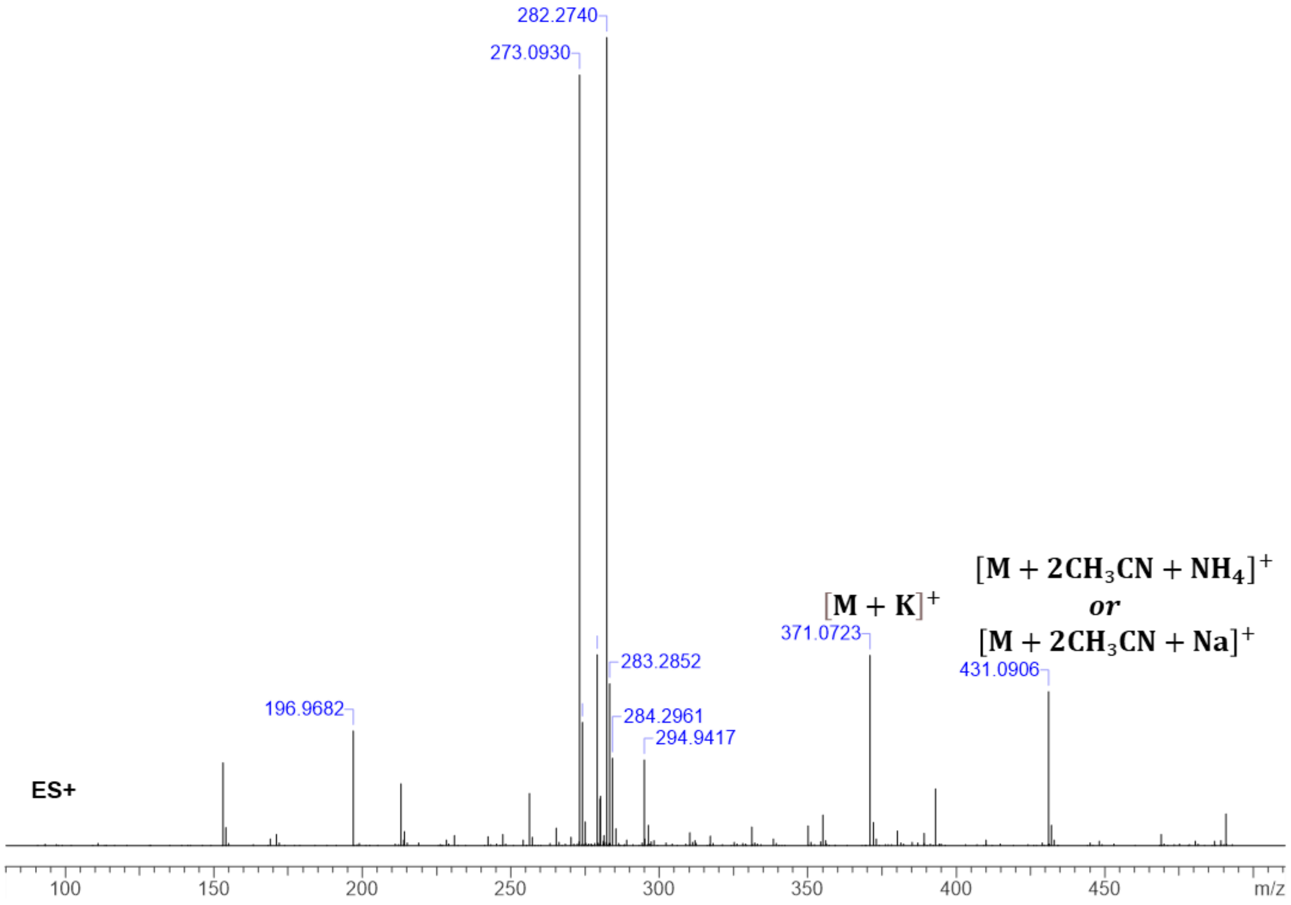

MS (ESI): calculated for $C_{14}H_{20}O_9K$ $[M + K]^+$, 371.0744; found 371.0723; calculated for $C_{18}H_{26}N_2O_9Na$ $[M + 2CH_3CN + Na]^+$, 437.1536; $C_{18}H_{26}N_2O_9Na$ $[M + 2CH_3CN + NH_4]^+$, 432.1982; found 431.0906.

Figure S51. Mass spectra of tetraacetyl-*α*-*L*-fucose (**13**) analyzed in CH_3CN.

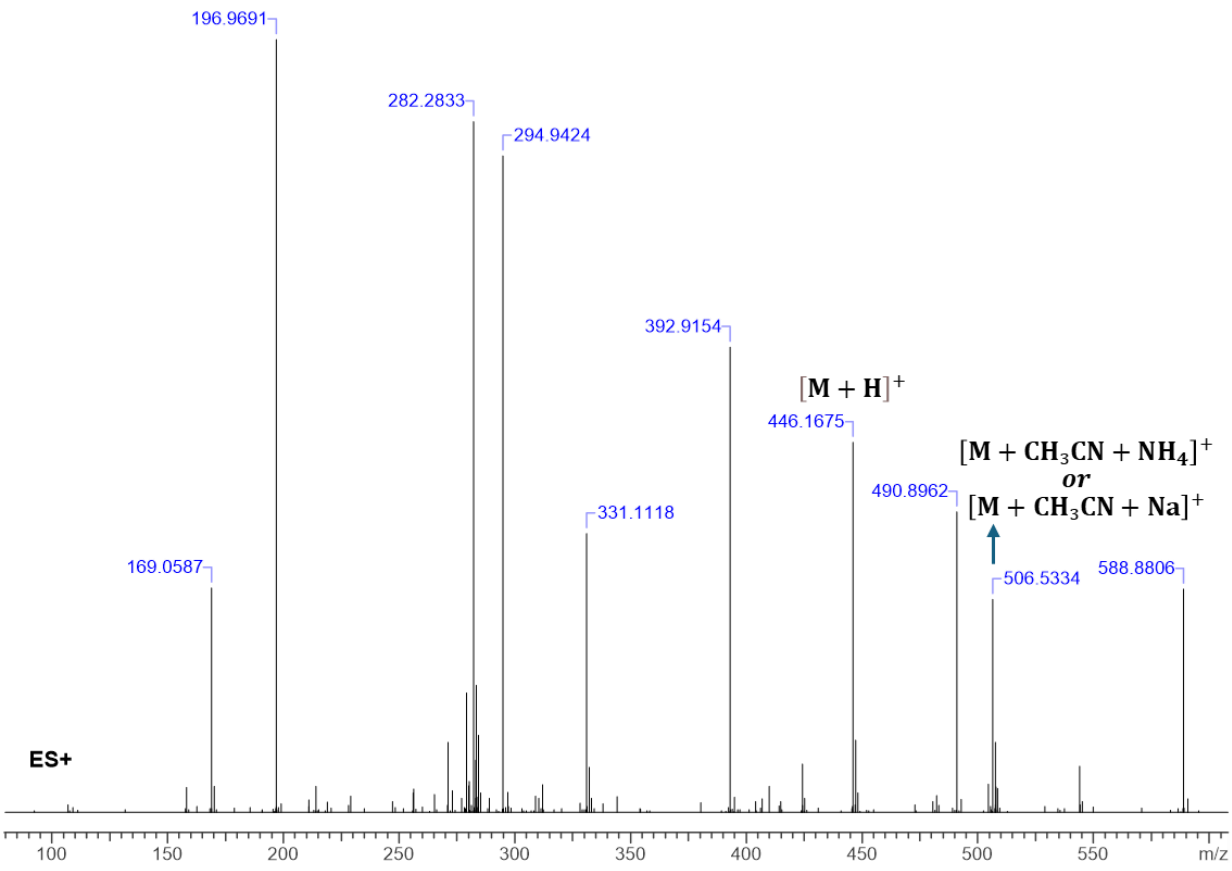

MS (ESI): calculated for $C_{19}H_{27}NO_{11}H$ [M + H]$^+$, 446.1662; found 446.1675; calculated for $C_{21}H_{30}N_2O_{11}Na$ [M + CH_3CN + Na]$^+$, 509.1747; calculated for [M + CH_3CN + NH_4]$^+$, 504.2193; found 506.5334

Figure S52. Mass spectra of GlucosEAm (**14, M3**) analyzed in CH_3CN.

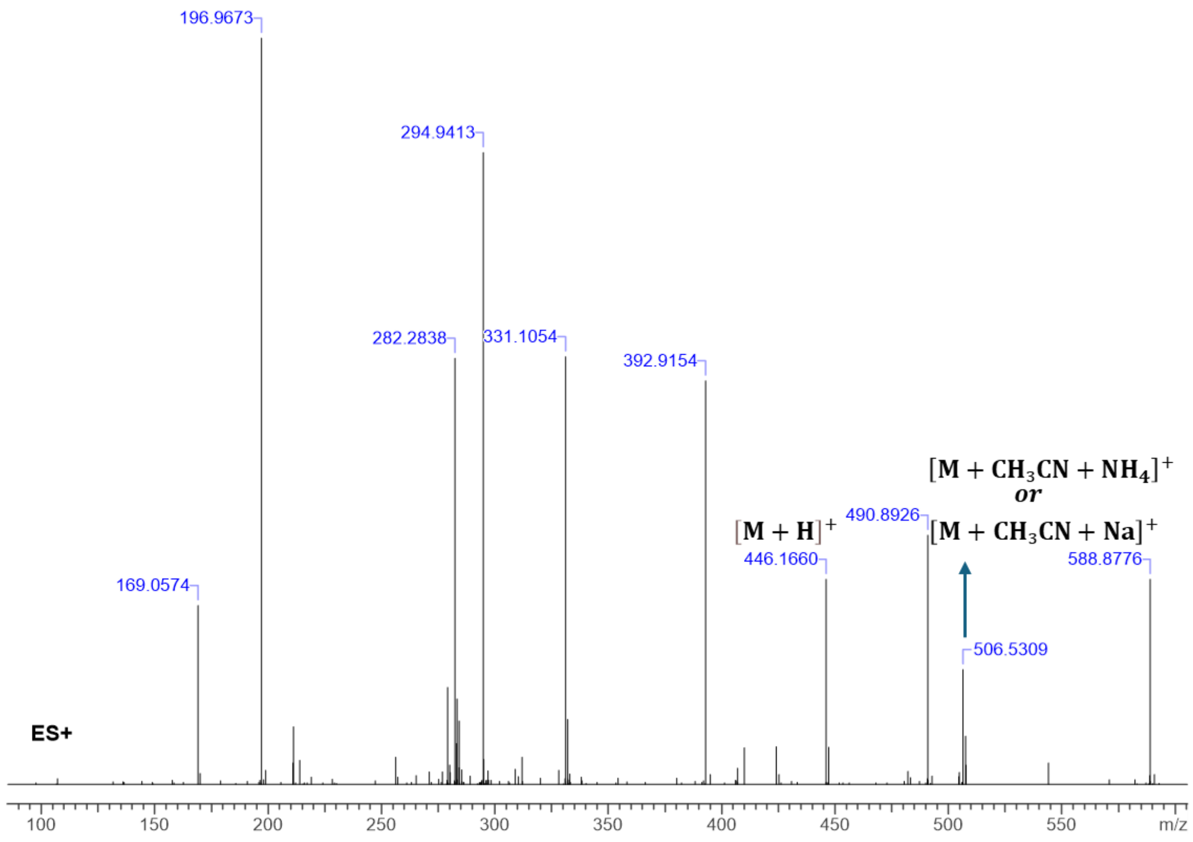

MS (ESI): calculated for $C_{19}H_{27}NO_{11}H$ [M + H]$^+$, 446.1662; found 446.1660; calculated for $C_{21}H_{30}N_2O_{11}Na$ [M + CH_3CN + Na]$^+$, 509.1747; found 506.5309; calculated for [M + CH_3CN + NH_4]$^+$, 504.2193; found 506.5309; also found 588.8776.

Figure S53. Mass spectra of GalactosEAm (**15, M4**) analyzed in CH_3CN.

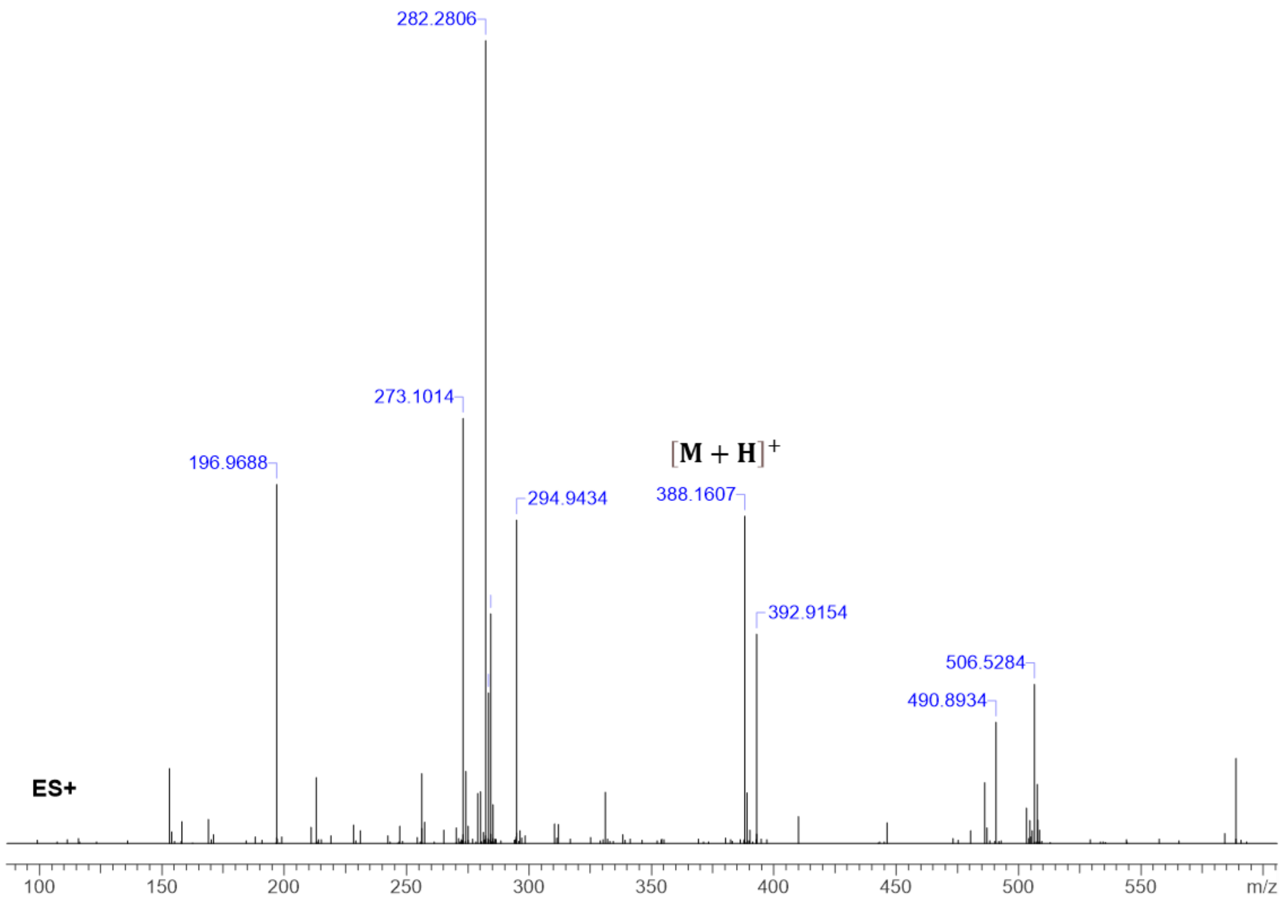

MS (ESI): calculated for $C_{17}H_{25}NO_9H$ $[M + H]^+$, 388.1601; found 388.1607

Figure S54. Mass spectra of FucosEAm (**16, M5**) analyzed in CH_3CN.

3.4 ^{1}H NMR spectra of representative sugar polymers in solution:

3.4.1 Ketose polymer (fructose):

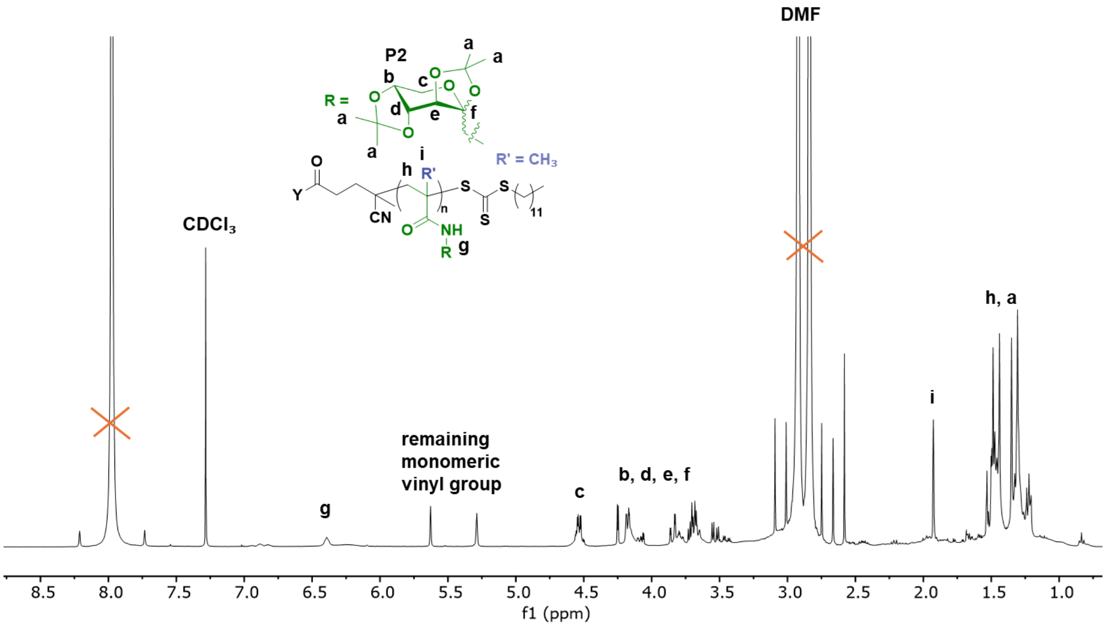

Figure S55. ^{1}H NMR (in $CDCl_3$) of poly(2,3:4,5-Di-*O*-isopropylidene-*β*-*D*-fructopyranose methacrylamide) (**P2**, PDIFMAm) in solution. Polymerization was carried out in DMF.

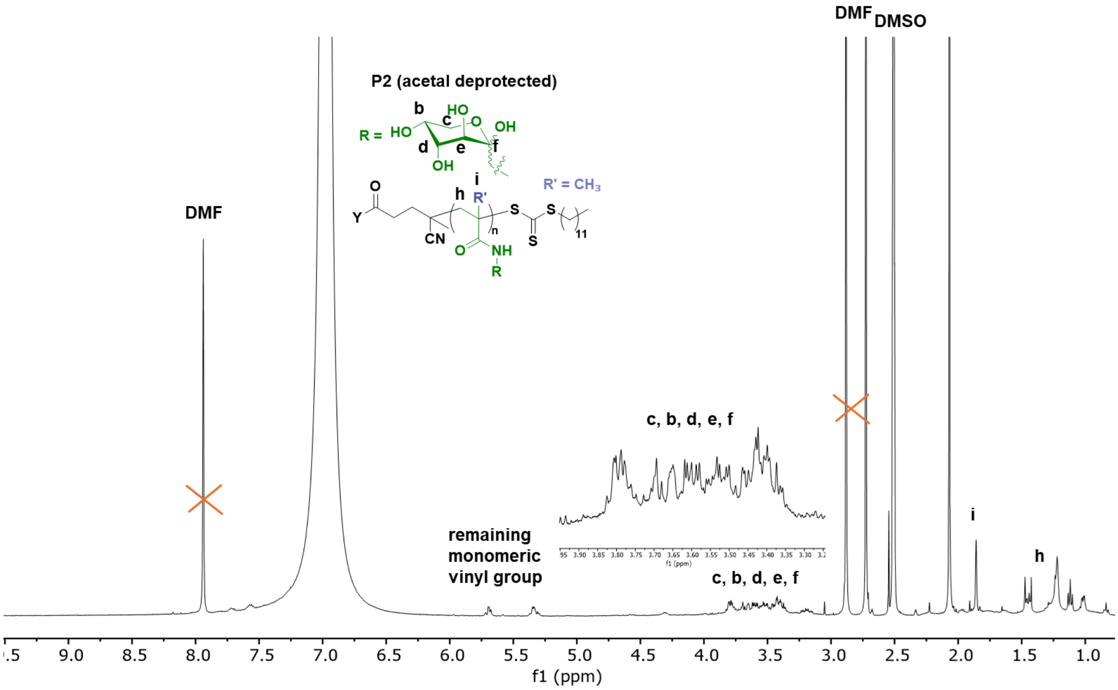

Figure S56. ^{1}H NMR (in DMSO, d_6) of deprotected (**P2**) polymer in solution. Deprotection was done with TFA:water (9:1) overnight. Zoomed in region (ppm 3.35 – 3.85) represent protons in the sugar region without any distinct separation as they are in the solution. Isopropylidene signals (shown in **Figure S54** at ~ppm 1 – 1.55) is diminished after deprotection. Polymerization was performed in DMF that was not entirely removed prior to deprotection.

3.4.2 Aldose polymers:

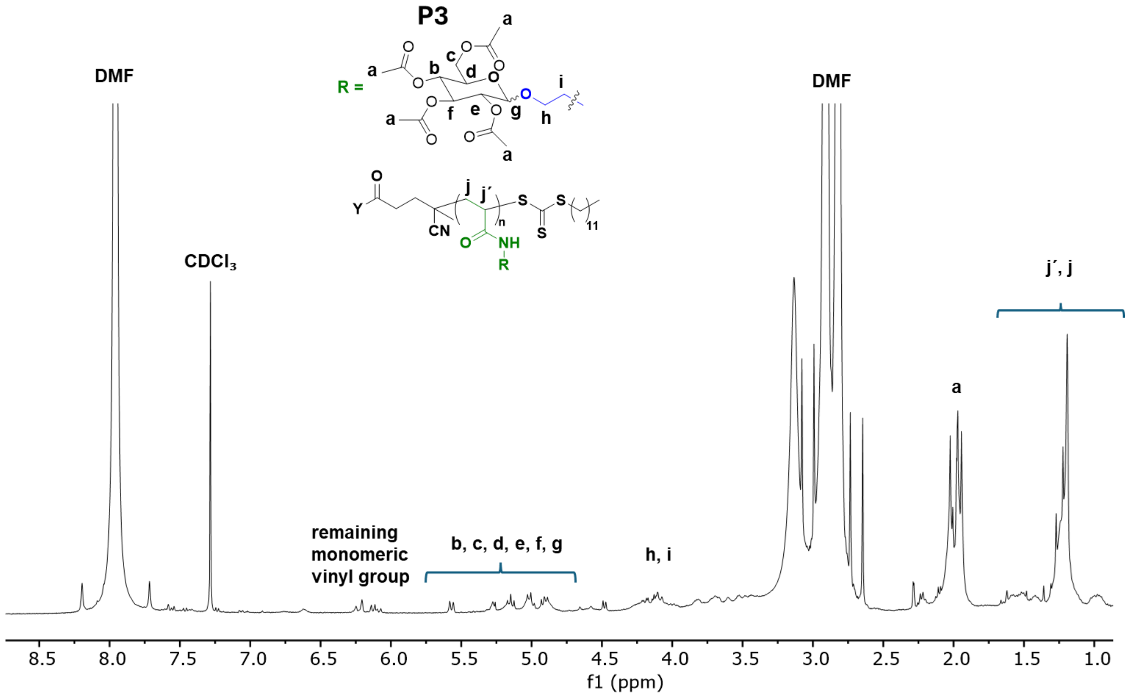

Figure S57. ^{1}H NMR (in $CDCl_3$) of poly(GlucosEAm), **P3** in solution. Polymers formed in the solution represent the characteristics of grafted polymers on wafer surface.

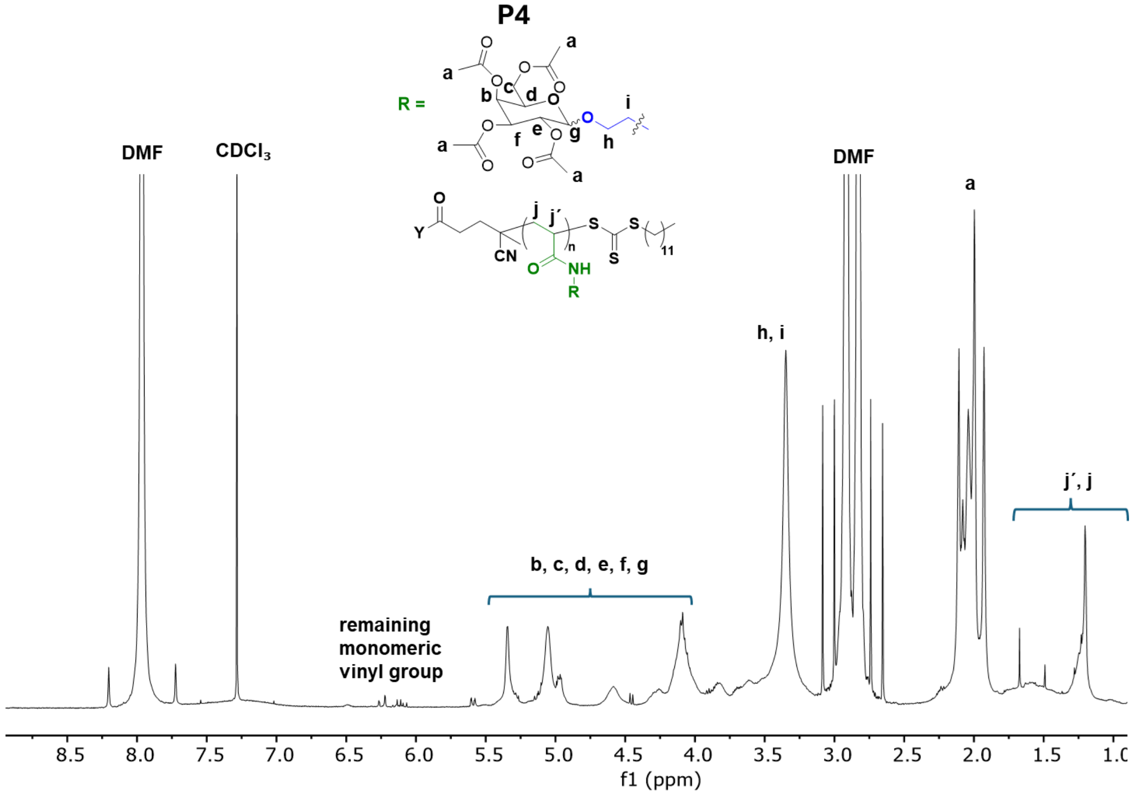

Figure S58. ^{1}H NMR (in $CDCl_3$) of poly(GalactosEAm), **P4** in solution. Polymers formed in the solution represent the characteristics of grafted polymers on wafer surface.

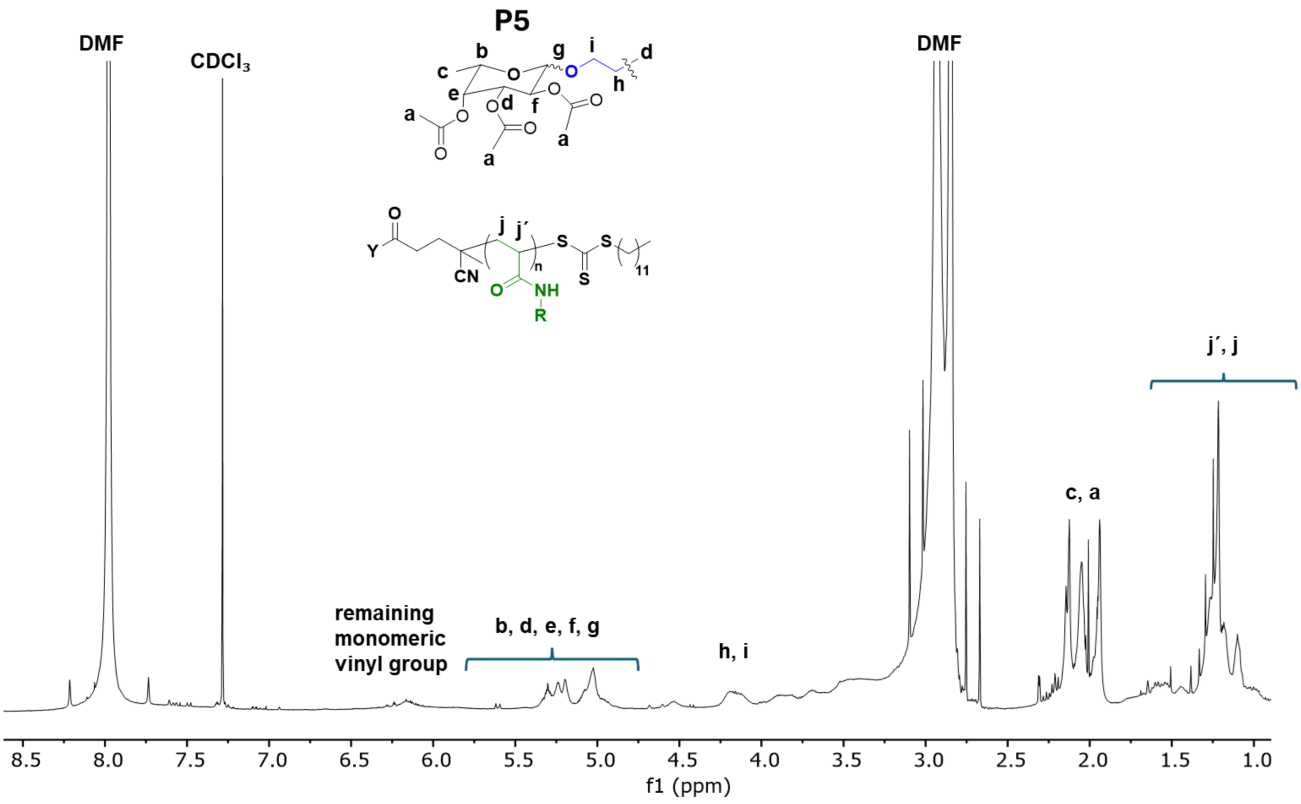

Figure S59. ^{1}H NMR (in $CDCl_3$) of poly(FucosEAm) **P5** in solution. Polymers formed in the solution represent the characteristics of grafted polymers on wafer surface.

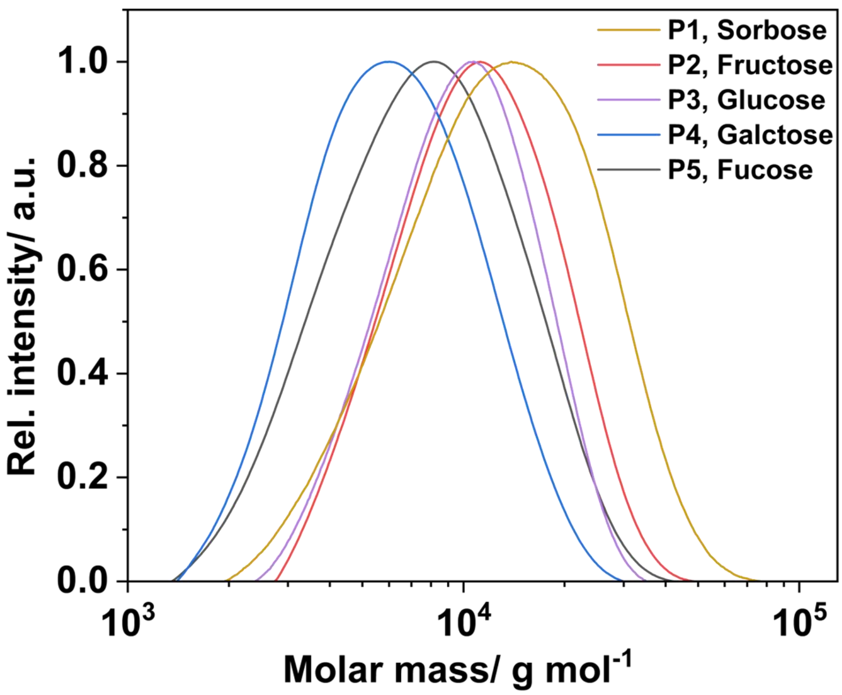

Figure S60. SEC profiles of thepolymers formed in solution. THF was used as an eluent (PSS standard). The solution was directly collected from the reaction vial and dissolved in the SEC eleuent for analysis.

Table S2. Number average molecular weight and dispersity index of the measured sugar polymers in solution. SEC data was measured in THF as an eluent (PSS standard).

Polymer sample	M_n / kg mol^{-1}	Đ
P1	9.5	1.30
P2	10	1.50
P3	8.5	1.26
P4	5.3	1.36
P5	6.2	1.45

3.4.3 Kinetic study of saccharide polymerization in solution:

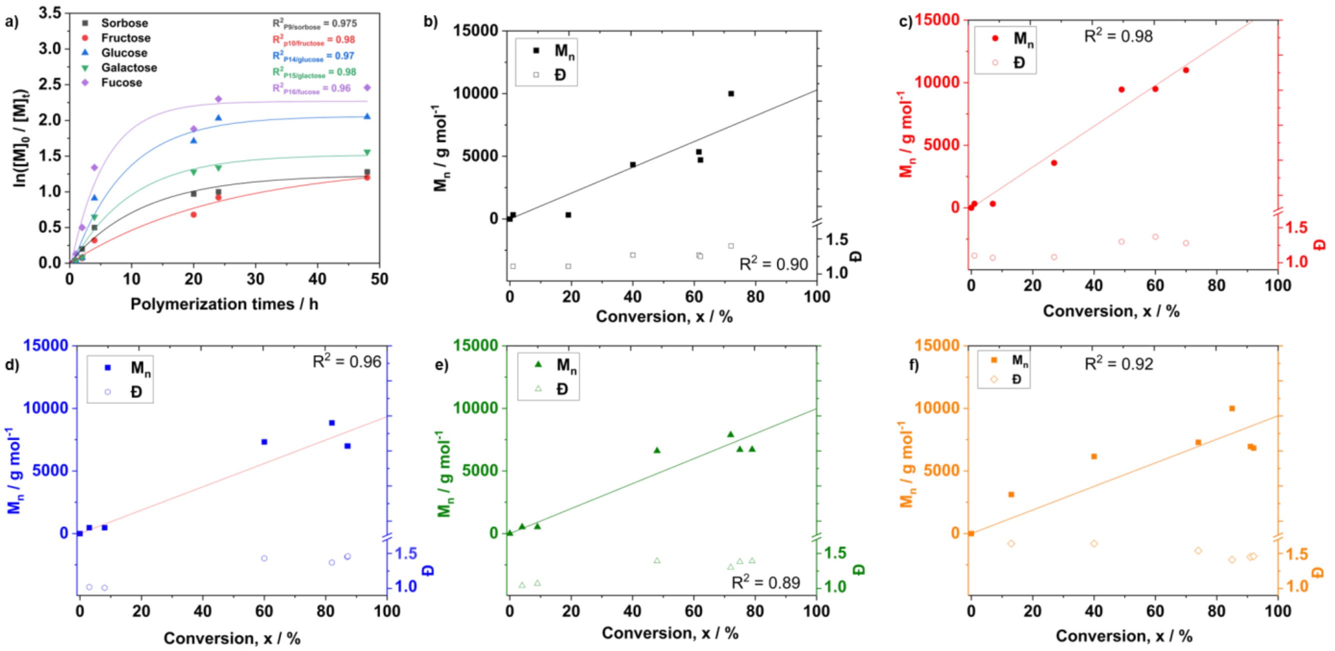

Figure S61. a) ln([M]$_0$ /[M]$_t$) vs polymerization time of saccharide monomers. The curves were fitted on OriginPro in with exponential function (Levenberg Marquardt algorithm). The reaction was run for 48 h and samples were collected at different intervals (0, 1, 2, 4, 20, 24, 48 h). After 24 h significant conversion was noticed (sorbose = 62%, fructose = 60%, glucose = 87%, galactose = 75%, fucose = 91%). b - f) Number average molecular weights (M_n) measured and dispersity (Đ) (measured *via* SEC, PS standard, THF as eluent) are plotted against observed monomers conversion. 7 data points were collected which show molecular weight of the formed polymer increase gradually with higher conversion as depicted with a linear fit model. For aldose monomers a small decrease in molecular weight is seen.

3.4.4 Water contact angle measurements (sugar modified surfaces):

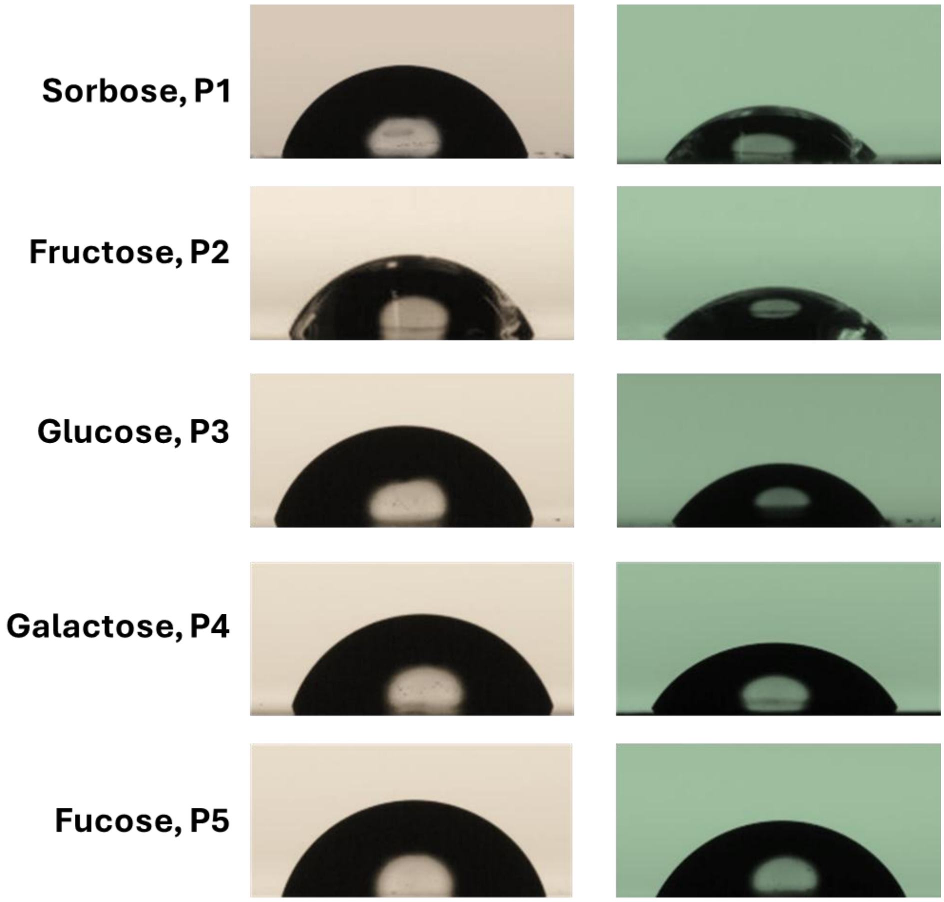

Figure S62. Water contact angles (CAs) of the Si wafer before (left) and after (right) deprotecting the protecting units (hydroxy groups of ketose monomers were protected with di-isopropylidene moieties and aldoses with penta-acetate groups) of the grafted polymers from the wafer-surface.

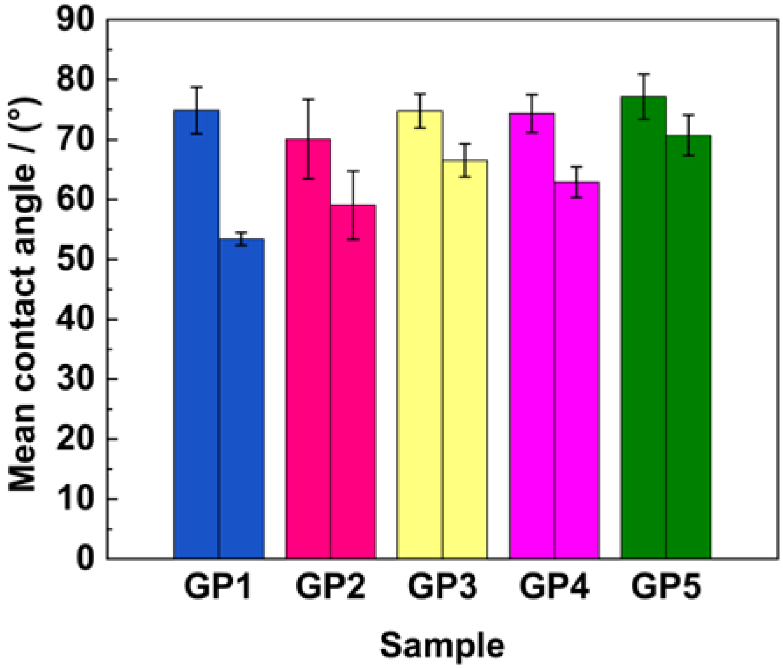

Figure S63. Mean water contact angles (CAs) of protected (ketose monomers with di-isopropylidene moieties and aldoses with penta-acetate groups) and deprotected grafted polymers on the wafer surfaces. The left and right bars of a combined bar indicate before and after glycosidic deprotection of the grafted sugar polymer. On average 5 drops were applied to obtain the mean values. The standard deviation values are shown in the bracket represent the errors.

3.5 Printing on Sugar substrates

3.5.1 Printing on Sugar Substrate (printing for different duration):

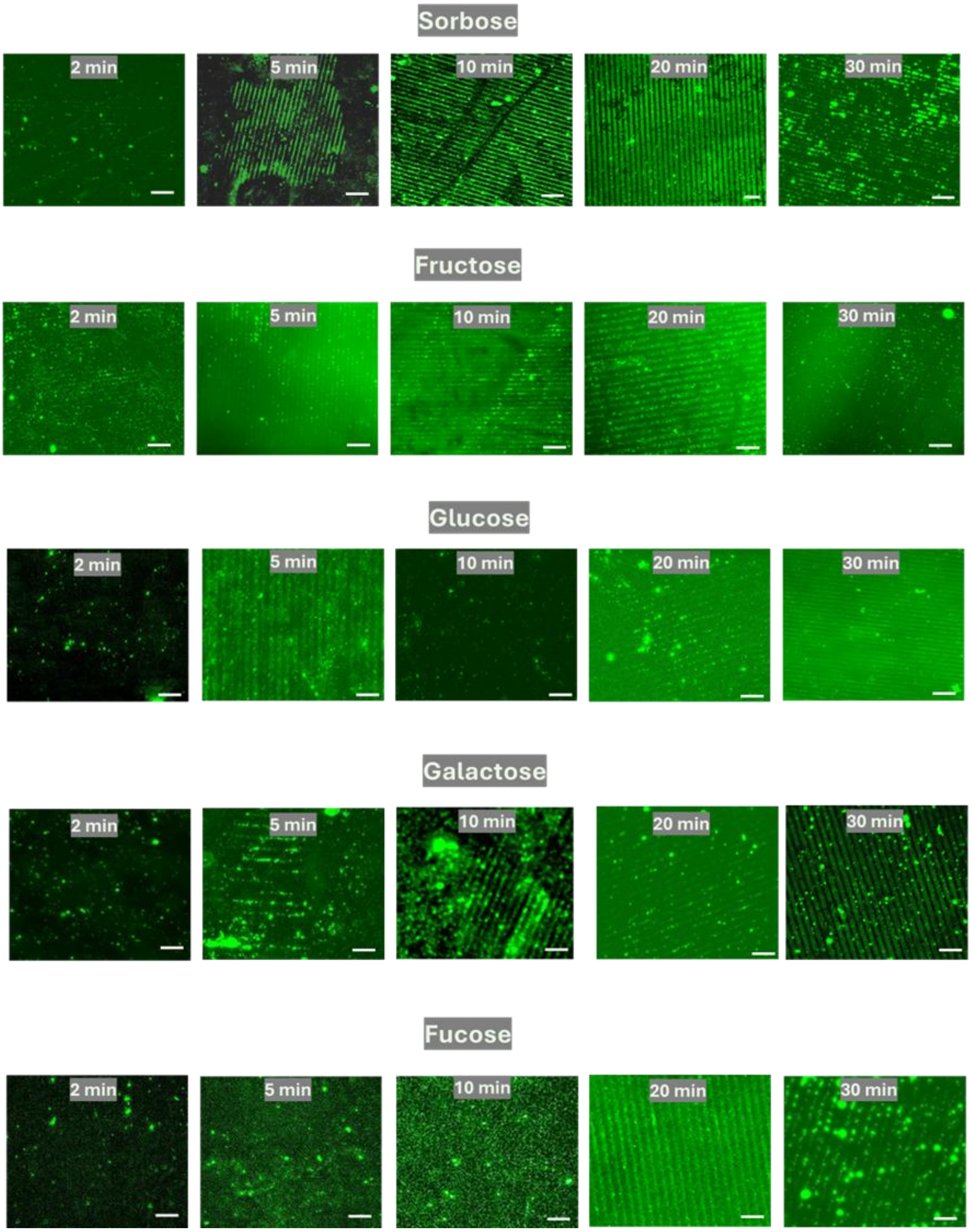

Figure S64. Printing ABOB on sugar modified surfaces for different printing duration (2 – 30 min). Ketose sugars demonstrate accurate patterns after 10 min and the accuracy decreases when printed for 30 min. However, printed aldose sugar substrates show almost no pattern at low timescale (2-10 min) and more accurate patterns when printed for 30 min. The scale bars are 20 μm.

3.5.2 Printing on Sugar Substrate (multiple printing):

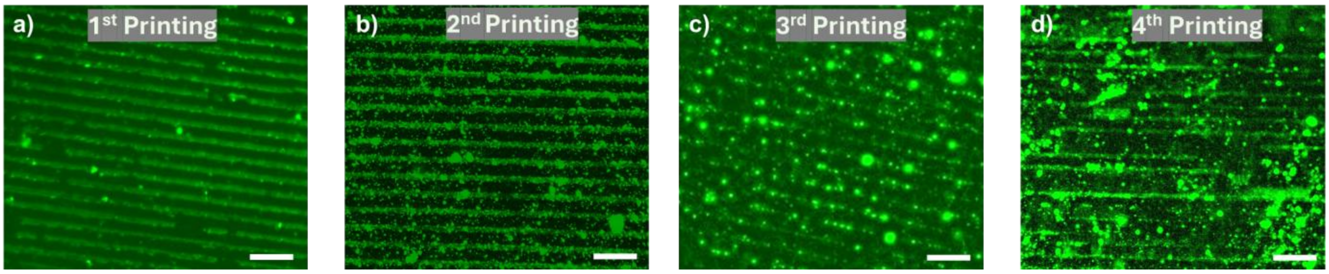

Figure S65. Printing on sorbose modified surfaces 4 times with a similar stamp after reinking the stamp each time after printing. 3rd and 4th printing have less resolution compared to the first two printing. The scale bars are 20 µm.

3.5.3 Printing on Sugar Substrate (negative control, pH 4 buffer wash):

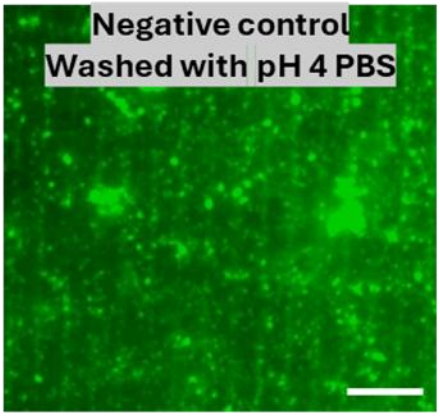

Figure S66. Removed stripe patterns from printed fructose surface. After printing the printed surface was washed with pH 4 PBS buffer to remove the patterns. The scale bar is 20 µm.

3.6 Binding assay:

3.6.1 competitive binding assay (BOB-ARS and BOB-sugar complexes):

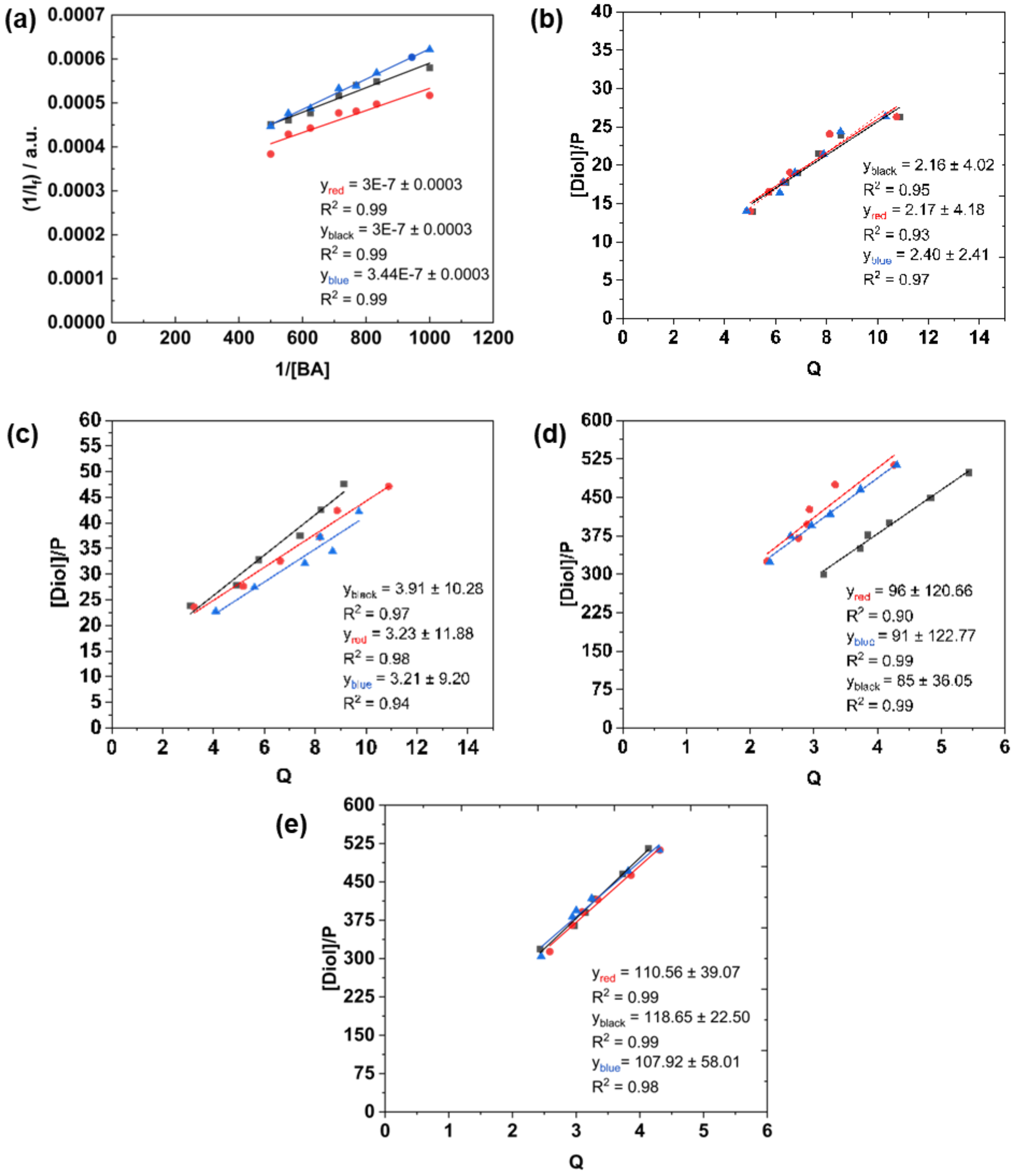

Figure S67. a) Titration curves of benzoxaborole (BOB) with alizarin red S (ARS) at pH 7.4 determined from the derived equation from previous literature.[13] Determining BOB-ARS is crucial for determining the binding constant of BOB-sugar complexes. Titration curves BOB at pH 7.4 with b) *L*-sorbose, c) *D*-fructose, d) *D*-galactose, and e) *D*-glucose. Three readings were recorded for each sugar.

To determine the K_{ars}, $(\frac{1/l_f}{1/[BA]})$ graph was plotted. The values were determined according to Sumerlin and co-workers.[13]

To determine the K_{eq}, $(\frac{[diol]/P}{Q})$ graph was plotted. P and Q value were determined according to Sumerlin and co-workers.[13]

3.6.2 Binding assay (BOB-fructose and BOB-sorbose complexes):

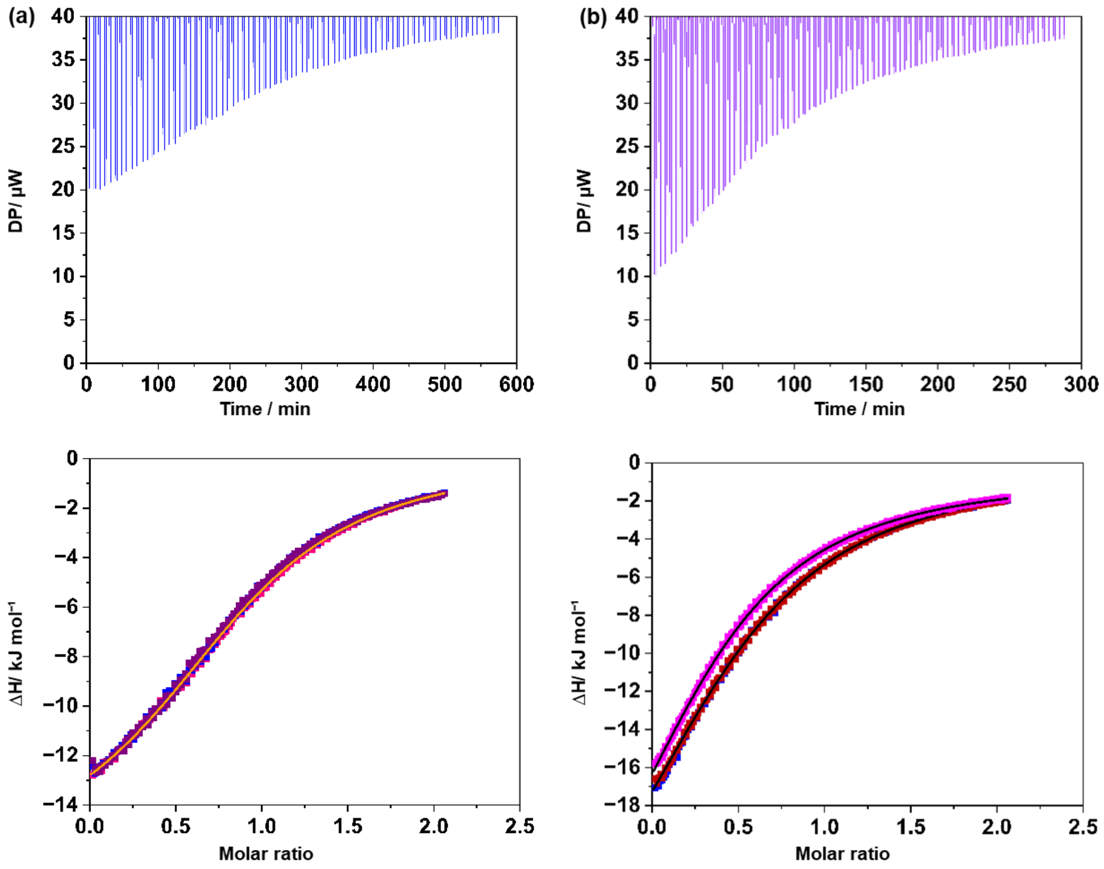

Figure S68. Isothermal calorimetry (ITC) plots to determine the binding constant of a) *L*-sorbose and b) *D*-fructose with benzoxaborole (BOB). Representative sample injection curves are shown on top along with triplicate data of the binding assays. 100 mM sugar in 100 mM PBS buffer pH 7.4 titrated into a solution of 10 mM BOB in 100 mM PBS buffer pH 7.4 at 298 K.

The isotherms were analyzed using the built-in MicroCal PEAQ-ITC analysis software, employing its fitting routine designed for a single set of identical binding sites. This approach incorporates key parameters, including the binding constant (K), free concentration of ligand in the active volume ([X]), free concentration of macromolecule in the active volume ([M]), fraction of sites occupied by ligand (Θ), the number of binding sites (n), and the molar heat of binding (ΔH). The following equations are therefore necessary.

$$K = \frac{\Theta}{(1-\Theta)[X]} \quad \text{(eq. 1)}$$

$$X_t = [X] + n\Theta M_t \quad \text{(eq. 2)}$$

By combining eq. 1 and 2, eq. 3 can be derived,

$$\Theta^2 - \Theta\,[1 + \frac{X_t}{nM_t} + \frac{1}{nKM_t}] + \frac{X_t}{nM_t} = 0 \quad \text{(eq. 3)}$$

The total heat content Q of the solution contained in V_0 (determined relative to zero for the unliganded species) at fractional saturation Θ is,

$$Q = n\Theta M_t\,\Delta H V_0 \quad \text{(eq. 4)}$$

Θ can be solved from eq. 3 to substitute its value in eq. 4 to give,

$$Q = \frac{nM_t\,\Delta H V_0}{2}\left[1 + \frac{X_t}{nM_t} + \frac{1}{nKM_t} - \sqrt{\left(1 + \frac{X_t}{nM_t} + \frac{1}{nKM_t}\right)^2 - \frac{4X_t}{nM_t}}\,\right] \quad \text{(eq. 5)}$$

The value of Q above can be calculated (for any designated values of n, K, and ΔH) at the end of the ith injection and designated Q (i). The expression for Q in eq. 5 only applies to the liquid contained in volume V_0. The heat released during the ith injection, ΔQ (i), accounts for the change in heat content between the completion of the (i − 1)th injection and the (i)th injection. The liquid injected during this process (ΔV_i) displaces some of the liquid originally in the working volume (V_0). While the displaced liquid passes out of the volume V_0, it still contributes to the overall heat effect (about 50% as much as the liquid that remains in V_0). A correction is therefore applied to include this contribution, ensuring accurate calculation of the heat released, assuming that reactions and mixing occur quickly.

$$\Delta Q\ (i) = Q(i) + \frac{dV_i}{V_0}\,[\frac{Q(i) + Q(i-1)}{2}] - Q(i-1) \quad \text{(eq. 6)}$$

Fitting experimental data involves using initial guesses for n, K, and ΔH (provided by MicroCal PEAQ-ITC software) to calculate ΔQ (i) and compare it with experimental data. These values are refined iteratively using Marquardt methods until the fit no longer improves.

3.7 Printing on microgel particles:

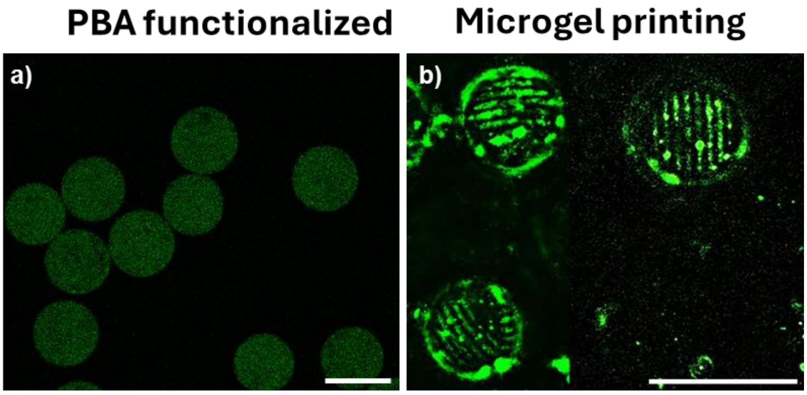

Figure S69. a) confocal microscopy images of dextran attached BOB-functionalized particles. b) Fluorescence microscopy images of Printed 4 μm stripe patterns on microgels. The scale bars are 100 μm.

3.8 Printing on cell membrane:

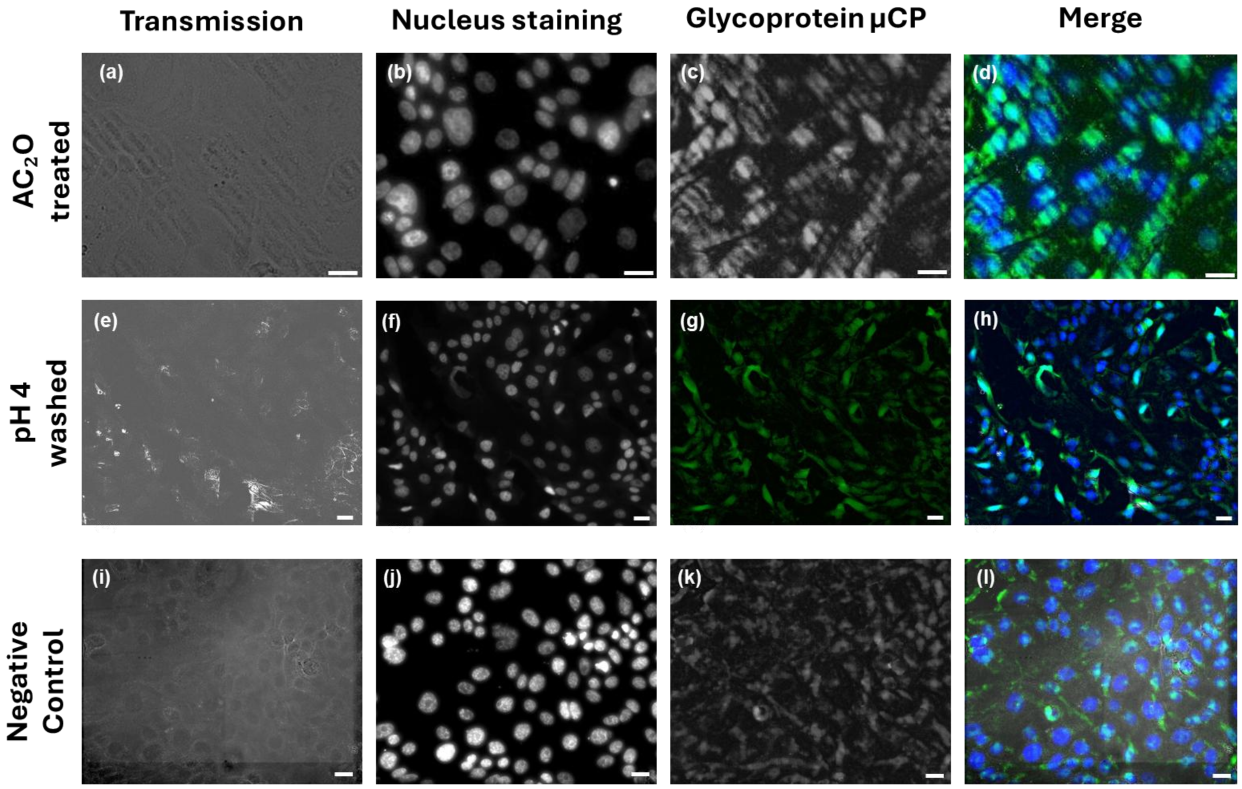

Figure S70. Printing on epithelial cell surfaces. a - d) acetic anhydride treated cells to protect any amino groups present on the cell membrane. e- h) Printed cell membrane after washed with pH 4 buffer solution. i – l) Printed with a bare stamp (no grafted polymer from the stamp surface, negative control). The scale bars are 20 µm.

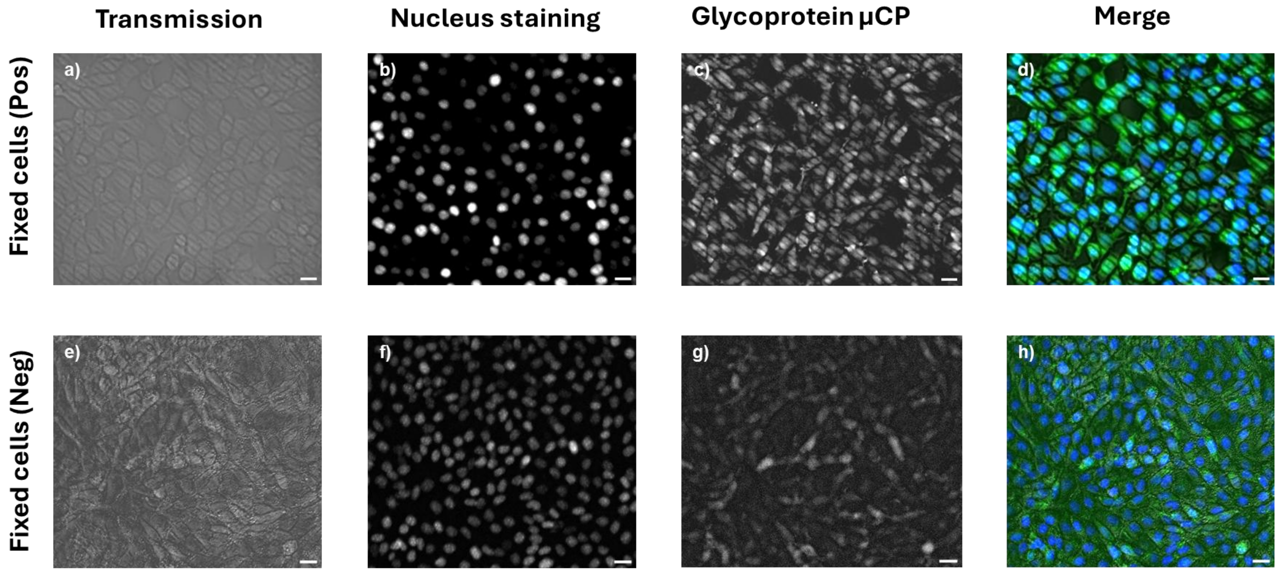

Figure S71. Printing on epithelial cell surfaces (cell line L929). a - d) Positive control. e – h) Printed with a bare stamp (no grafted polymer from the stamp surface, negative control). The scale bars are 20 µm.

4. References

[1] G. M. Sheldrick, *Acta Crystallogr. C Struct. Chem.* **2015**, *71*, 3–8.

[2] G. M. Sheldrick, *Acta Crystallogr. A. Found. Crystallogr.* **2008**, *64*, 112–122.

[3] C. F. Macrae, I. Sovago, S. J. Cottrell, P. T. A. Galek, P. McCabe, E. Pidcock, M. Platings, G. P. Shields, J. S. Stevens, M. Towler, P. A. Wood, *J. Appl. Crystallogr.* **2020**, *53*, 226–235.

[4] Crystal structures data (DIF-N3: CCCD 2393133; DIFAm: CCCD 2393131; DISOTf: CCCD 2393174; DIS-N3: CCCD 2393305) can be obtained free of charge from The Cambridge Crystallographic Data Centre, http://www.ccdc.cam.ac.uk.

[5] N. Pallab, S. Reinicke, J. Gurke, R. Rihm, S. Kogikoski, M. Hartlieb, M. Reifarth, *Polym. Chem.* **2024**, *15*, 853–867.

[6] P. Glass, H. Chung, N. R. Washburn, M. Sitti, *Langmuir* **2009**, *25*, 6607–6612.

[7] J. Yang, I. Bos, W. Pranger, A. Stuiver, A. H. Velders, M. A. Cohen Stuart, M. Kamperman, *J. Mater. Chem. A* **2016**, *4*, 6868–6877.

[8] S. Yu, Y. Liu, C. Shang, Y. Du, J. Liu, *Tetrahedron. Lett.* **2021**, *72*, 153072.

[9] B. E. Maryanoff, D. F. McComsey, M. J. Costanzo, C. Hochman, V. Smith-Swintosky, R. P. Shank, *J. Med. Chem.* **2005**, *48*, 1941–1947.

[10] Y. Toyoshima, A. Kawamura, Y. Takashima, T. Miyata, *J. Mater. Chem. B* **2022**, *10*, 6644–6654.

[11] M. Calosso, D. Charpentier, M. Vaillancourt, M. Bencheqroun, G. St-Pierre, B. C. Wilkes, Y. Guindon, *ACS Med. Chem. Lett.* **2012**, *3*, 1045–1049.

[12] R. De Coen, N. Vanparijs, M. D. P. Risseeuw, L. Lybaert, B. Louage, S. De Koker, V. Kumar, J. Grooten, L. Taylor, N. Ayres, S. Van Calenbergh, L. Nuhn, B. G. De Geest, *Biomacromolecules* **2016**, *17*, 2479–2488.

[13] W. L. A. Brooks, C. C. Deng, B. S. Sumerlin, *ACS Omega* **2018**, *3*, 17863–17870.

[14] D. Vrbata, V. Ďorďovič, J. Seitsonen, J. Ruokolainen, O. Janoušková, M. Uchman, P. Matějíček, *Chem. Commun.* **2019**, *55*, 2900–2903.

[15] M. G. López, D. W. Gruenwedel, *Carbohydr. Res.* **1991**, *212*, 37–45.
